# Trabecular Bone Ontogeny of the Human Distal Tibia

**DOI:** 10.1002/ajpa.25043

**Published:** 2024-12-08

**Authors:** Rebecca A. G. Reid, Catriona Davies, Craig Cunningham

**Affiliations:** ^1^ Centre for Anatomy and Human Identification, School of Science and Engineering University of Dundee Dundee Scotland

**Keywords:** bipedalism, microcomputed tomography, ontogeny, tibia, trabecular analysis

## Abstract

**Objectives:**

There is an increasing understanding of how trabecular bone adapts to biomechanical changes during ontogeny. However, limited research exists regarding the distal tibia, which is important in weight‐bearing locomotion as part of the ankle joint. This study aims to document the ontogenetic trabecular patterns of the distal tibia, in addition to changes in its structural heterogeneity.

**Materials and Methods:**

Thirty‐eight distal tibiae, ranging in age from 28 intrauterine weeks to 8 postnatal years, from the Scheuer juvenile skeletal collection were examined. Trabecular bone was analyzed using a quantitative volume of interest approach and qualitative whole bone mapping following microcomputed tomography.

**Results:**

Fetal and perinatal tibia lack mature organization and are associated with high bone volume fraction. During the first year of life, there is a decrease in bone volume fraction and an indication of early re‐organization of trabecular struts in the distal tibia. After one year of age, the distal tibia exhibits increased trabecular structural heterogeneity.

**Discussion:**

The trabecular architecture of the fetal and perinatal distal tibia lacks mature organization and instead reflects ossification patterns. At these stages, there is a rapid accumulation of bone mass associated with gestational overproduction, hypothesized to be in preparation for subsequent postnatal changes. During the first year of life there is a decrease in volume fraction, associated with constructive regression. It is postulated this is related to changing biomechanical forces associated with the bipedal gait, in addition to growth demands. After one year of age, the distal tibia exhibits structural heterogeneity with trabecular adaption to accommodate specific bipedal stresses.

## 1 Introduction

An understanding of the development of the human trabecular skeleton and its adaptation to biomechanical influences has both clinical and forensic applications. This includes the treatment and analysis of juvenile trauma and pathology. Previous research has investigated the juvenile femur (Milovanovic et al. 2017; Reissis and Abel 2012; Ryan and Krovitz 2006; Ryan, Raichlen, and Gosman 2017), humerus (Perchalski et al. 2018; Reissis and Abel 2012; Ryan, Raichlen, and Gosman 2017), radius (Colombo et al. 2019), vertebrae (Acquaah et al. 2015; Nuzzo et al. 2003), pelvic complex (Cunningham and Black 2009; Maclean, Black, and Cunningham 2014), and pedal bones (Figus et al. 2022; Figus, Sorrentino, et al. 2023; Figus, Stephens, et al. 2023; Saers, Ryan, and Stock 2019). However, while research on the proximal tibia exists (Goliath et al. 2022; Gosman and Ketcham 2009), there is limited research on the juvenile distal tibia (Raichlen et al. 2015).

The ankle, or talocrural joint, is a hinge‐type synovial joint involving the distal ends of the tibia and fibula which form the malleolar mortise into which the trochlea of the talus articulates. The talocrural joint is stabilized by the medial (deltoid) ligament and the lateral ligament (Golanó et al. 2010). The deltoid ligament is comprised of four portions, the tibionavicular, tibiocalcaneal, and anterior and posterior tibiotalar ligaments which originate from the medial malleolus and insert on the navicular, calcaneus, and talus respectively (Golanó et al. 2010). The tibiotalar joint is the load‐bearing aspect of the talocrural joint (Brockett and Chapman 2016). The movements of the talocrural joint are dorsiflexion and plantarflexion. The tibia also articulates distally with the fibula at the inferior tibiofibular joint. The inferior tibiofibular joint has a vital function in talocrural joint stability as it maintains close contact between the lateral malleolus and lateral talus. This stability is maintained by the interosseus membrane, and the anterior and posterior tibiofibular ligaments holding the tibia and fibula together distally. The distal tibia therefore has an important role in weight‐bearing and in the production of movements required for bipedal gait.

Previous research on the distal tibia examined the extent to which trabeculae are aligned, known as the degree of anisotropy (DA), between 1 and 9 years of age (Raichlen et al. 2015). Inter‐individual variation in DA was higher in younger individuals in comparison to more mature individuals. It was hypothesized that this greater extent of variation in DA between individuals at a younger age was related to the idiosyncratic nature of the process of learning to walk. Once the bipedal gait was refined, it was postulated that the variation in DA decreased. Our previous radiographic research into the distal tibiae indicated that the distal tibia lacks organization during the fetal and perinatal period, before radiodensity decreased during the first two years of life, and subsequently became organized to accommodate bipedal locomotion (Reid, Davies, and Cunningham 2023).

While previous research indicates that the development of the bipedal gait does influence trabecular ontogeny of the distal tibia, it does not provide insight into how other trabecular parameters such as bone volume fraction (BV/TV), trabecular separation (Tb.Sp), trabecular thickness (Tb.Th), and trabecular number (Tb.N) change throughout development. Additionally, the trabecular architecture of the distal tibial epiphysis lacks investigation. Given the weight‐bearing role of the distal tibia as part of the ankle joint, further research is necessary to understand exactly how this bone adapts to changing biomechanical influences during development.

### 1.1 Development of Human Locomotion

The juvenile skeleton experiences changing biomechanical influences during the attainment of different motor milestones. Biomechanical influences are present from the early intrauterine period. At approximately 7 weeks gestation, spontaneous muscle contractions begin (Rose and Gamble 1994). Regular intrauterine movements occur from 15 weeks, with activity levels increasing throughout the last few months of the gestational period (de Vries, Visser, and Prechtl 1982; Verbruggen et al. 2018). Movement of the fetus against the uterus wall accompanied by resistance of the amniotic fluid, in addition to skeletal loading by fetal muscle contractions, are various forms of intrauterine loading (Miller 2003). Early ‘new‐born stepping’ may occur between 32 weeks gestation and 2 postnatal months (Rose and Gamble 1994). The onset of crawling occurs at approximately 4–7 months of age, however, the mode of crawling attained is variable between individuals (Adolph, Vereijken, and Denny 1998).

Unassisted walking occurs at approximately 12 months of age (Keen 1993). Early walking is typically described as having a wide stance with simultaneous flexion of the knee and hip, an absence of complete knee and hip extension, and an absence of heel strike (Cowgill et al. 2010; Hallemans et al. 2005; Johnston, Eastwood, and Jacobs 2014; Sutherland, Olshen, and Biden 1988; Sutherland 1997). The initial gait pattern associated with early walking is extremely irregular and is associated with variable walking strategies, stride lengths and widths, and foot rotation (Adolph, Vereijken, and Shrout 2003; Bisi and Stagni 2015). Muscle activity of the lower limbs is also highly variable during early walking (Chang et al. 2006), with gross activation of muscles that lack a standard pattern of muscle activity (Okamoto, Okamoto, and Andrew 2003). Around 18–24 months heel‐strike evolves, with ankle plantarflexion during gait development (Sutherland, Olshen, and Biden 1988; Zeininger et al. 2018). This is linked to the development of the longitudinal arch of the foot, which begins to appear when the foot is loaded during bipedal gait but does not mature until 6 years of age (Bosch, Gerss, and Rosenbaum 2007). During early walking, the plantar fat pad defends the foot against overloading, dispersing the plantar pressure throughout the foot and the entire foot is in contact with the ground (Bosch, Gerss, and Rosenbaum 2007). As a result, peak plantar pressures within the midfoot are greater in infants in comparison to adults (Hallemans et al. 2003). During the development of the longitudinal arch, the fat pad shifts toward the heel and forefoot while the morphology of the hindfoot bones matures, and therefore, there is a gradual shift in load from the midfoot to the heel and forefoot (Bertsch et al. 2004; Bosch, Gerss, and Rosenbaum 2007). In a mature gait, after heel striking, the foot will roll forward to encounter the ground. When rolling forward of the foot is absent initially during immature gait, the medial side of the foot has greater loading (Bertsch et al. 2004; Hallemans et al. 2003). With the progression of unassisted walking, this load gradually shifts to the lateral side of the foot (Hallemans et al. 2003).

With experience, the bipedal gait becomes more standardized, which has been observed beginning two months after the onset of unassisted walking (Bisi and Stagni 2015). There is substantial variation within the literature regarding the maturation of gait, however, it is commonly stated to mature between 7 and 9 years (Keen 1993).

### 1.2 Development and Biomechanics of the Distal Tibia

Ossification of the tibia begins at the midshaft at approximately 53 days of intrauterine development and advances toward the distal and proximal ends of the bone (Baumgart et al. 2019; Gardner, Gray, and O'rahilly 1959). Vascular invasion is evident at the end of the embryonic period in the tibia diaphysis (Cunningham, Scheuer, and Black 2016; Gardner, Gray, and O'rahilly 1959). During the perinatal period, the tibia is represented as a shaft and a proximal epiphysis (Cunningham, Scheuer, and Black 2016). Activation of muscles such as the tibialis anterior to produce ankle dorsi‐flexion may occur at this stage as part of ‘new‐born stepping’ (Okamoto, Okamoto, and Andrew 2003). ‘New‐born stepping’ occurs when the infant is supported in an upright position, therefore the tibia is unlikely to experience full bodyweight forces at this stage. From 4 months of age, crawling may develop, however, the knee is typically weight‐bearing at this stage rather than the distal tibia within the ankle (Adolph, Vereijken, and Denny 1998; Righetti et al. 2015). The secondary ossification center of the distal tibia ossifies during the first year of life (Cunningham, Scheuer, and Black 2016; Love, Ganey, and Ogden 1990). This occurs around the same time as the attainment of unassisted walking (Keen 1993). As confidence in the bipedal gait develops, bodyweight forces through the tibia may increase as unassisted walking progresses (Keen 1993). Additionally, shearing forces may occur within the anterior and posterior distal tibia as heel strike develops at 18–24 months (Sutherland, Olshen, and Biden 1988; Zeininger et al. 2018). Between 3 and 4 years, the medial malleolus begins to appear (Baker, Tocheri, and Dupras 2005; Love, Ganey, and Ogden 1990) and becomes recognizable at 4–5 years of age (Cunningham, Scheuer, and Black 2016). Fusion of the distal tibial epiphysis occurs in males at 16–20 years and 14–18 years in females (Cunningham, Scheuer, and Black 2016).

The mature distal tibia is therefore subjected to compressive forces from body weight, in addition to tensile forces in the medial malleolus due to the ankle collateral ligaments (Tillmann, Bartz, and Schleicher 1985). The distal articular surface of the adult tibia has been described as containing thick, vertically aligned trabeculae (Takechi et al. 1982) with high BV/TV levels (Tsegai et al. 2017), which are thought to be adapted to facilitate the transmission of high compressive bodyweight forces through to the talus (Procter and Paul 1982; Stauffer, Chao, and Brewster 1977). The trabeculae nearest the talar facet within the adult medial malleolus were observed to be transversely orientated (Takechi et al. 1982) and are hypothesized to facilitate the dispersal of tensile forces associated with the collateral ligaments. Within the adult tibial metaphysis, both Du et al. (2019) and Sode et al. (2010) observed high BV/TV and Tb.Th with plate‐like structured trabeculae within the posterior and medial regions. Additionally, the trabecular architecture in the inner region displayed lower BV/TV, Tb.Th, and Tb.N with higher Tb.Sp than the outer region (Sode et al. 2010). Both Du et al. (2019) and Sode et al. (2010) attribute the high BV/TV levels in the medial and posterior regions of the adult distal tibia to large compressive and shearing forces applied in these areas. However, the changes in the trabecular architecture of the juvenile distal tibia remain elusive.

Changes in the trabecular architecture of the juvenile distal tibia will be investigated and related to the changing biomechanical environment during ontogeny to provide insight into the development of the trabecular skeleton. A detailed understanding of the regional trabecular changes within the juvenile distal tibia may be useful in the therapeutic intervention of pathological conditions associated with the ankle joint, such as juvenile idiopathic arthritis. Finally, the normal juvenile trabecular arrangement may be informative for the interpretation of juvenile skeletal trauma, such as classic metaphyseal lesions (Love, Derrick, and Wiersema 2011).

### 1.3 Trabecular Development

Patterns in the development of the trabecular skeleton have emerged with increased research.

The fetal period is defined by an accumulation of bone mass, described by Acquaah et al. (2015) as ‘gestational overproduction’. This is demonstrated by the increasing BV/TV values during in utero development (Acquaah et al. 2015; Nuzzo et al. 2003; Salle et al. 2002). While BV/TV increases, changes in Tb.Th and Tb.N vary throughout the fetal skeleton at this stage (Acquaah et al. 2015; Nuzzo et al. 2003; Salle et al. 2002). In addition to changes in trabecular quantity, Acquaah et al. (2015) observed a shift from rod‐shaped to plate‐like trabeculae, while DA also decreased in the vertebrae during the gestational period. The high BV/TV levels reported intrauterine in addition to plate‐like trabeculae may be linked to calcium storage, presenting a large surface area for mineral release, required for subsequent postnatal changes (Acquaah et al. 2015). While the vertebrae and femur exhibited a lack of structural organization intrauterine, an organized structure consistent with adult form was observed in the ilium, sacrum, and scapula (Cunningham and Black 2009; O'Malley 2013; Yusof 2013), in addition to fetal nonhuman species (Skedros, Hunt, and Bloebaum 2004; Skedros et al. 2007). This prenatal organization was hypothesized to be due to a pre‐determined genetic template and/or the influence of vascularization (Cunningham and Black 2010, 2013).

As a result of the increase in bone mass during gestational development, high BV/TV values have been observed in the perinatal skeleton (Acquaah et al. 2015; Beresheim, Pfeiffer, and Grynpas 2020; Colombo et al. 2019; Figus et al. 2022; Figus, Stephens, et al. 2023; Gosman and Ketcham 2009; Milovanovic et al. 2017; Ryan and Krovitz 2006; Ryan, Raichlen, and Gosman 2017; Saers, Ryan, and Stock 2019). At this stage, the trabecular architecture of bone has been regularly described as typically dense and isotropic with numerous thin trabeculae (Djuric et al. 2012; Gosman and Ketcham 2009; Saers, Ryan, and Stock 2019).

The first year of life marks a distinct shift in trabecular quantity and organization, defined as ‘constructive regression’ (Acquaah et al. 2015). During this stage, a decrease in BV/TV has been observed throughout the weight‐bearing bones of the skeleton (Acquaah et al. 2015; Figus, Stephens, et al. 2023; Gosman and Ketcham 2009; Milovanovic et al. 2017; Ryan and Krovitz 2006; Ryan, Raichlen, and Gosman 2017; Saers, Ryan, and Stock 2019). The juvenile ribs, however, did not exhibit this constructive regression, while the distal radius experienced this change earlier than reported in bones associated with bipedal locomotion (Beresheim, Pfeiffer, and Grynpas 2020; Colombo et al. 2019). It has been hypothesized that this constructive regression reflects bone (re)modeling to accommodate for the development of the bipedal gait, with this occurring earlier in the radius due to weight‐bearing during crawling (Acquaah et al. 2015; Colombo et al. 2019).

Changes in trabecular quantity are also accompanied by changes in the trabecular architecture during the first year of life. A shift from rod‐shaped to plate‐like trabeculae has been observed (Acquaah et al. 2015; Milovanovic et al. 2017; Ryan and Krovitz 2006), while DA decreases in the femur (Milovanovic et al. 2017; Ryan and Krovitz 2006) and the tibia (Gosman and Ketcham 2009). In comparison, DA increased in the calcaneus and talus (Figus et al. 2022; Saers, Ryan, and Stock 2019). These conflicting trends in DA are hypothesized to be related to different ossification patterns of irregularity in comparison to weight‐bearing bones.

Early trabecular form therefore comprises gestational overproduction and ossification‐dictated organization of early bone followed by bone resorption during constructive regression (Acquaah et al. 2015; Saers et al. 2022b). This has been described as the ‘pre‐locomotor’ phase by Saers et al. (2022a, 2022b).

After 1 year, the trabecular skeleton experiences ‘refinement’ (Acquaah et al. 2015). Overall, there is an increase in BV/TV during this age range, however, the exact timings and rates of this change vary depending on the skeletal element (Figus et al. 2022; Figus, Sorrentino, et al. 2023; Goliath et al. 2022; Milovanovic et al. 2017; Ryan and Krovitz 2006; Ryan, Raichlen, and Gosman 2017; Saers, Ryan, and Stock 2019). Additionally, DA increases and diverges in values dependent on location within a bone as the skeleton becomes more organized to specific loads (Acquaah et al. 2015; Gosman and Ketcham 2009; Milovanovic et al. 2017; Ryan and Krovitz 2006; Saers, Ryan, and Stock 2019). This is consistent with the ‘neuromaturation phase’ proposed by Saers et al. (2022a, 2022b) occurring between the onset of locomotion and the achievement of adult gait, whereby gait matures as neurodevelopment advances resulting in adaptation of the trabecular skeleton. Typically, adult trabecular quantity and structure are achieved at 8 years of age, as observed in the tibia (Gosman and Ketcham 2009), calcaneus (Saers, Ryan, and Stock 2019), and vertebrae (Roschger et al. 2001), however, changes have also been reported during adolescence (Figus, Sorrentino, et al. 2023; Goliath et al. 2022). This has been described as the ‘mature locomotion’ phase occurring between the achievement of an adult‐like gait and adulthood (Saers et al. 2022a).

### 1.4 Aims and Hypotheses

Given the limited research examining the development of trabecular bone with the distal tibia, this study aims to document the trabecular development of the distal tibia between 28 intrauterine weeks until 8 postnatal years. This study will test whether the progression of endochondral ossification, growth, and changes in biomechanical loading are reflected in the trabecular architecture of the distal tibia. This will be examined by comparing trabecular parameters DA, BV/TV, and Tb.Sp, Tb.Th, and Tb.N between different stages of development (inter‐developmental group analysis). Additionally, regional changes in these trabecular parameters will be investigated during each developmental stage by comparing their values between multiple different sites within the distal tibia (intra‐developmental group analysis). This will provide insight into whether trabecular ontogeny within the distal tibia follows the developmental patterns identified within other weight‐bearing bones, and if so, exactly how this occurs.

It is hypothesized that the distal tibia will experience high BV/TV during the fetal and perinatal period because of gestational overproduction (Acquaah et al. 2015). It is predicted that the pre‐loading tibia will lack a mature organization, and instead reflect the progression of endochondral ossification (Saers et al. 2022a, 2022b). During the first year of life, it is expected that a decrease in BV/TV associated with constructive regression will be observed (Acquaah et al. 2015). After 1 year of life, it is postulated that structural heterogeneity of the distal tibiae will increase and become refined in association with the acquisition and maturation of the bipedal gait (Acquaah et al. 2015; Saers et al. 2022a, 2022b). Specifically, it is predicted that by 8 years of age, the center of the tibia metaphysis trabecular will have lower BV/TV, Tb.Th, and Tb.N with higher Tb.Sp than the peripheries of the bone (Sode et al. 2010), with higher BV/TV within the posterior and medial regions (Du et al. 2019; Sode et al. 2010). The distal epiphysis is hypothesized to contain vertically aligned trabeculae, with trabeculae also orientated in the transverse direction within the medial malleolus (Takechi et al. 1982).

## 2 Materials and Methods

### 2.1 Sample

The distal end of 38 human tibiae, ranging from 28 intrauterine weeks to 8 years of age, were available for examination from the Scheuer Collection, Center for Anatomy and Human Identification, University of Dundee (Table 1). The left‐sided tibia was selected, and if not available or it had sustained considerable post‐mortem damage, the right side was utilized (Table 1). For the inter‐developmental group analysis, only individuals without post‐mortem damage that restricted all volumes of interest (VOI) being placed were used, reducing the total number of individuals for this analysis to 29 tibiae (Table 1). Individuals were scanned up to 8 years of age, as this is purportedly when the most significant changes in trabecular bone occur (Saers, Ryan, and Stock 2019). The Scheuer Collection is a juvenile skeletal collection from European archaeological and historical anatomical sources. Age‐at‐death is estimated for most of the sample.

**TABLE 1:** Sample composition for each developmental cohort.

Developmental group	Age	Side	Post‐mortem damage
Fetal 28–38 weeks *n* = 4	28 weeks in utero	L	
	28 weeks in utero	R	
	32 weeks in utero	R	x
	38 weeks	R	
Perinatal *n* = 17	Perinate	L	x
	Perinate	L	
	Perinate	L	x
	Perinate	L	
	Perinate	L	
	Perinate	R	x
	Perinate	R	
	Perinate	R	
	Perinate	R	x
	Perinate	R	
	Perinate	L	
	Perinate	L	
	Perinate	L	
	Perinate	L	x
	Perinate	L	
	Perinate	L	
	Perinate	L	
Infant and early toddler 0–≤ 2 years *n* = 7	0–6 months	L	
	4–6 months	L	x
	5 months	L	
	5 months	L	
	1 year	L	
	1–2 years	R	
	2 years	L	
Early childhood > 2–≤ 8 years *n* = 10	2–5 years	R	x
	3 years 4 months	L	
	3–6 years	L	
	4 years	L	
	4–5 years	L	
	6 years	L	
	6–10 years	L	
	6–8 years	R	
	7 years	L	
	8 years	L	x

*Note*: All specimens were included for the intra‐group analysis. Individuals with post‐mortem damage were excluded for the inter‐group analysis.

Individuals in the sample were divided into developmental groups based upon their estimated age‐at‐death. The fetal group was selected to investigate if and how ‘gestational overproduction’ progressed within the talus and distal tibia, while the perinatal group was chosen to provide a snapshot of the results of gestational overproduction (Acquaah et al. 2015). The infant group (0–1 year) was selected to investigate ‘constructive regression’ within the pre‐loading talus. This cohort was extended to include early toddlers (0–2 years) due to age‐at‐death being estimated for most of the sample. This means that as there were individuals estimated at 1–2 years, it was unclear whether these individuals would have started unassisted walking, which occurs on average between 12 and 14 months (Keen 1993). Given that some of these individuals appeared radiographically like younger individuals in terms of radiolucency, and thus were placed within radiographic groups consistent with constructive regression (Reid, Davies, and Cunningham 2023), it was decided to extend the infant group to include these individuals. The early and late childhood groups were divided at 8 years due to this being the time at which the bipedal gait typically matures (Saers, Ryan, and Stock 2019; Sutherland 1997). This approach of subdividing an ontogenetic sample into different groups based upon age‐at‐death has been previously adopted by Acquaah et al. (2015), Figus et al. (2022); Figus, Sorrentino, et al. (2023); Figus, Stephens, et al. (2023); Goliath et al. (2022), and Milovanovic et al. (2017).

### 2.2 Microcomputed Tomography Imaging

All tibiae were scanned within the School of Science and Engineering, University of Dundee using a Nikon XT H 225ST μCT scanner. Tibiae were mounted within Oasis Ideal Max Life Wet Floral Foam with parafilm stretched over the surface of the foam to prevent any foam particles from entering bone with cortical erosion or via the vascular foramina. Tibiae were scanned at 90–145 kV and 125–165 μA dependent on individual size, with an exposure time of 1000 ms using a 0.125 cm copper filter. The data were collected with 3141 projections. Filtered back projection reconstruction creating three‐dimensional (3D) volumes, ranging in voxel size from 13.55–44.90μm^3^, was performed using Volume Graphics VG STUDIO MAX 3.3. Two‐dimensional 16‐bit. TIFF image stacks were exported for further analysis.

### 2.3 Trabecular Analysis

Trabecular analysis was conducted via two approaches: a spherical VOI approach and whole bone trabecular mapping.

### 2.4 The volume of Interest Approach

**FIGURE 1:**
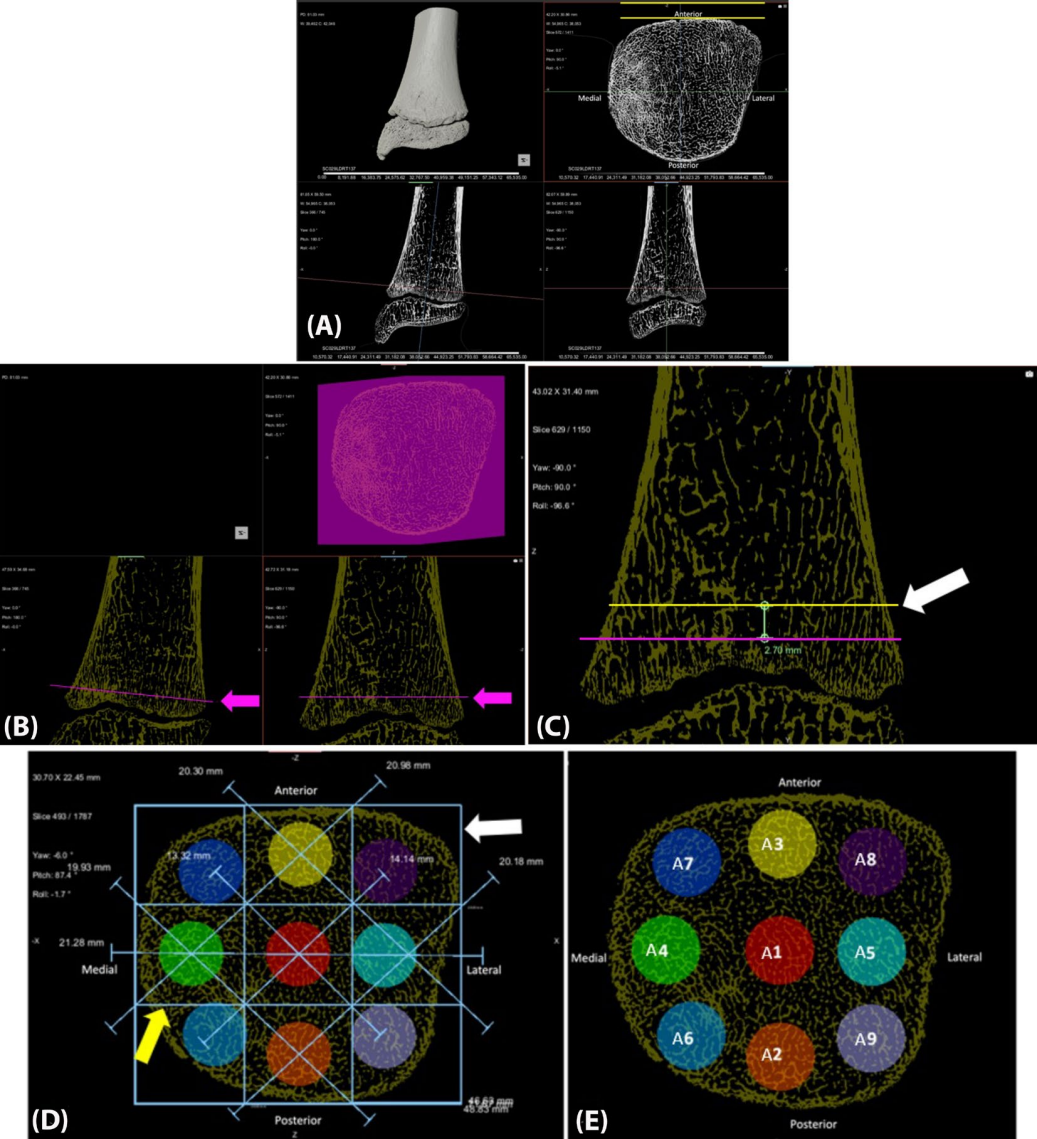
Orientation of the distal tibia and placement of metaphyseal volumes of interest. (A) Tibiae were placed in a standardized anatomical position (B) Demarcation of distal‐most slice with complete trabeculae. (C) Measurement of 0.5*VOI diameter superior (yellow line, white arrow) to highlighted pink slice. (D) A bounding box was placed on the extremities of the bone within the located slice, and then subdivided into a 9 × 9 grid (E) VOIs A1–9 were placed within the grid.

Dragonfly 2020.2 (Object Research Systems) was utilized for VOI trabecular analysis. The two‐dimensional 16‐bit. TIFF stacks for each tibia were imported into the Dragonfly software. Tibiae were placed in a standardized anatomical position so that the anterior surface of the tibia in the distal axial slice was parallel to the superior border of the viewing window within the axial view (Figure 1A, yellow lines). Tibiae were binarized using an automatic Otsu threshold algorithm to separate the image into mineralized and nonmineralized material (Otsu 1979).

**FIGURE 2:**
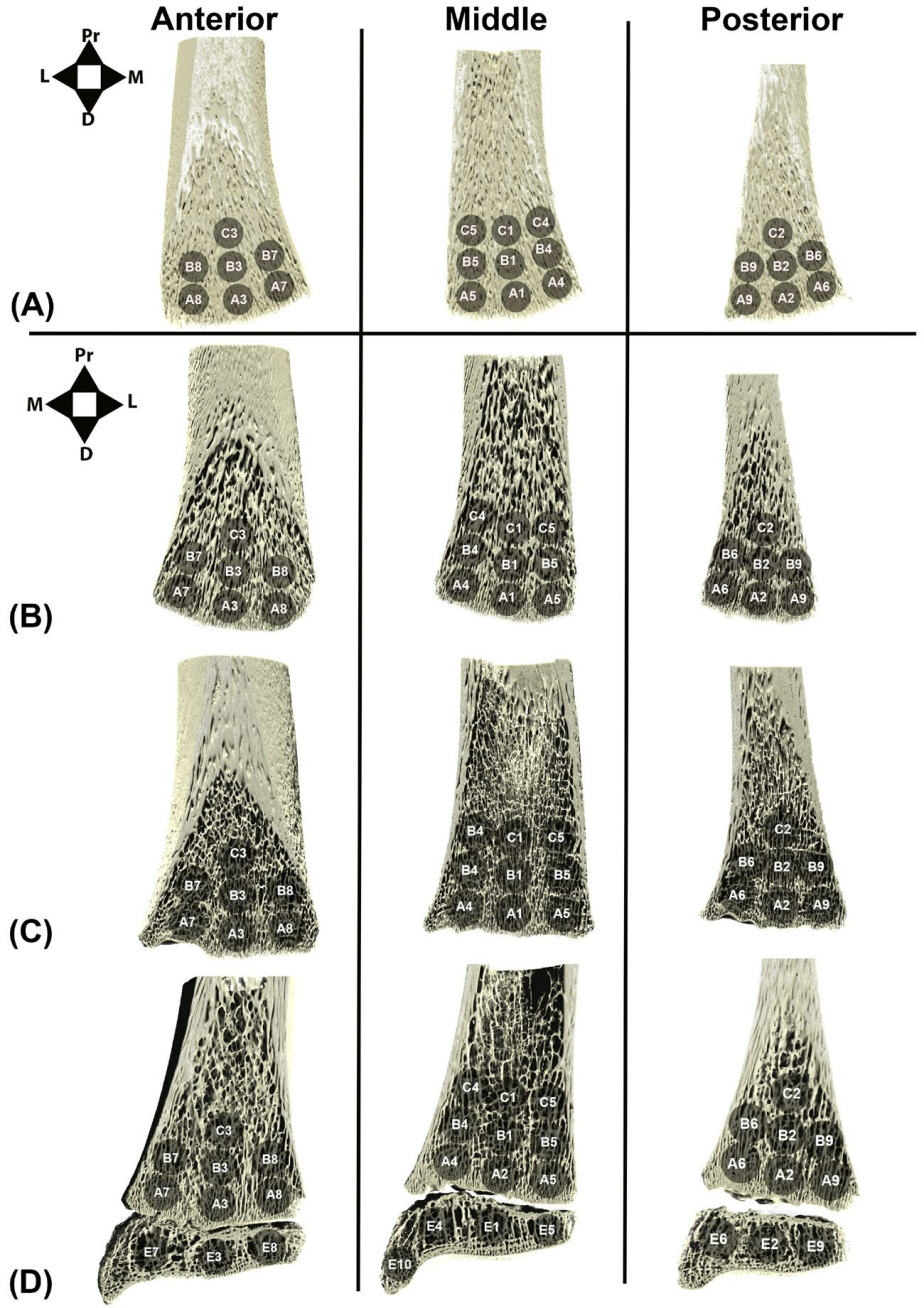
Representation of volume of interest placement in 3D coronal sections of (A) Fetal group, 28–38 weeks, (B) Perinatal group, (C) Infant and Early Toddler group, 0–2 years, and (D) Early Childhood group, > 2–8 years.

Twenty‐three spherical VOIs were placed in the distal tibial metaphysis and 10 VOIs were placed in the distal tibial epiphysis in a standardized method, scaled at 20% maximum anteroposterior width of the distal tibial metaphysis (Figure 2A–E). See Videos S1 and S2 for a three‐dimensional visualization of the VOI placement.

The following metaphyseal VOI placement procedure was utilized. The most‐distal slice with complete, uninterrupted trabeculae was visually selected and demarcated using the 2D region of interest painter (Figure 1B). To ensure that each VOI contained only trabeculae and did not extend into the cortical bone, 0.5*VOI diameter was measured superior to this highlighted pink slice to ensure the VOI did not extend below into an area without trabeculae (Figure 1C). The axial slice located at the superior aspect of this measurement was used to place the most‐distal aspect of VOIs (layer A) (Figure 1C). This point is located at the center of the row A VOIs (Figure 2). A bounding box was placed on the extremities of the bone within the located slice (Figure 1D). The width and breadth of the bounding box were measured using the ruler tool in Dragonfly, and then subdivided into a 9 × 9 grid (Figure 1D). For VOIs A1‐4, the center of the VOI was located by placing intersecting lines from the corners of each ‘grid square’ creating an ‘X' (Figure 1D,E). For VOI A5, this was repeated but an additional line was used to find the point at which the center of the grid square met the medial grid boundary to accommodate for the shape changes of the fibular notch throughout ontogeny (Figure 1D,E). The remaining VOIs A6–9 were placed in corners of grid squares (Figure 1D,E). Two additional rows of VOIs (B1–B9 and C1–C5) were placed superior to VOIs A1–A9 (Figure 2). The axial slice located immediately superior to VOIs A1–A9 containing no VOIs was located, and then 0.5*VOI diameter was measured superiorly to locate the center of VOIs B1–B9 were located. The same placement process for VOIs A1–A9 was utilized for VOIs B1–B9. The process of measuring 0.5*diameter superior to row B VOIs was repeated for VOIs C1–C5, however, the corner VOIs (VOIs 6–9) were not placed due to the more square‐like morphology of the tibia as it progressed superiorly. Additionally, VOI C5 was placed in the center of the ‘grid square’ due to the lack of the fibular notch superiorly within the tibia.

**FIGURE 3:**
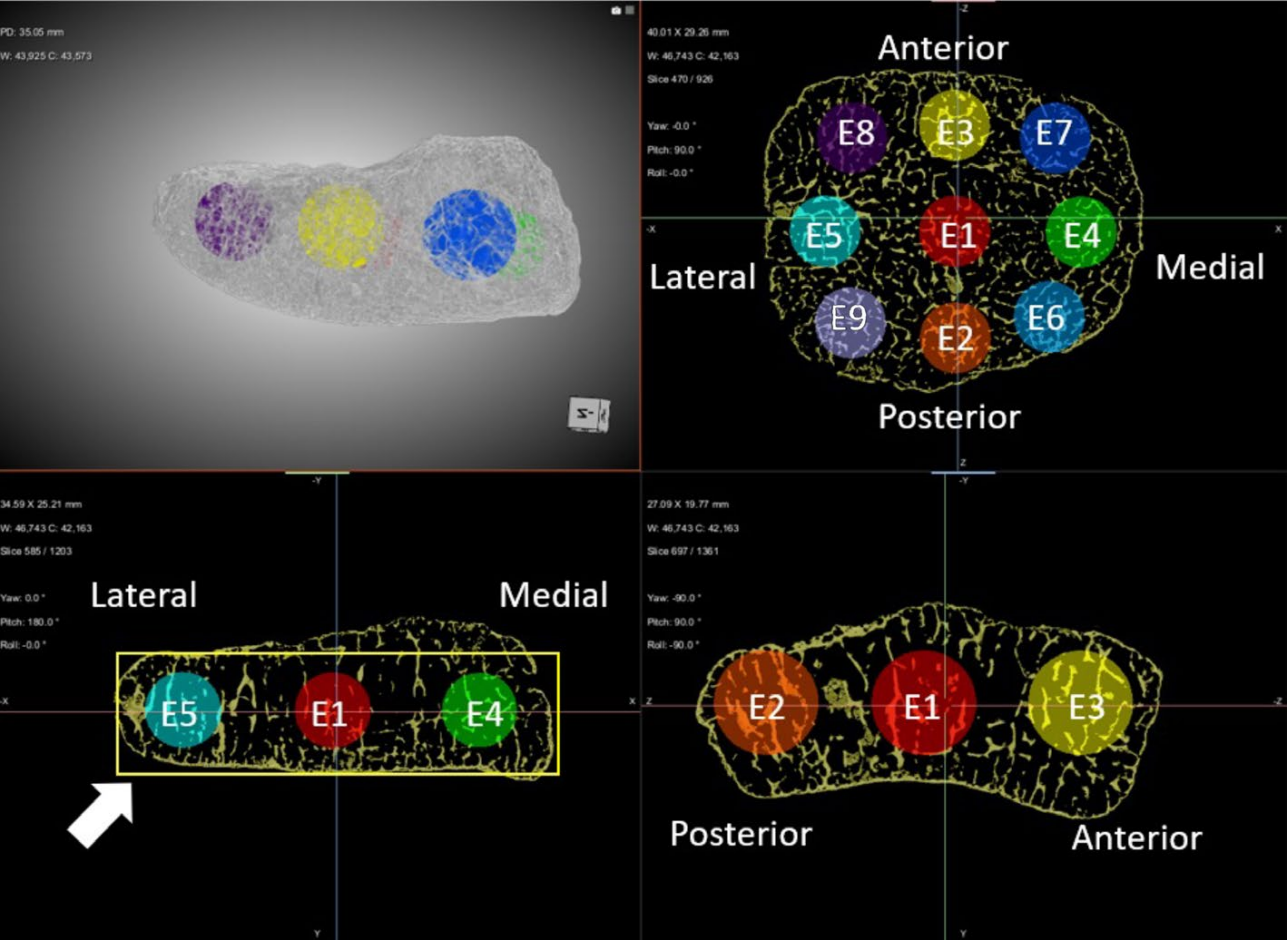
Placement of VOIs within the distal epiphysis. A bounding box was placed around the minimum height of the epiphysis within the coronal view (yellow box, white line) and the center of VOIs E1–E9 placed at mid‐height of the box.

To place VOIs within the distal epiphysis, a bounding box was placed around the minimum height of the epiphysis within the coronal view (Figure 3). The height of this box was measured, and then the center was located by measuring 0.5*bounding box height. This was used to locate the slice where the center of the epiphyseal VOIs was placed. The same strategy used for the placement of the 9 VOIs within the tibial metaphysis was used to place 9 VOIs within the center of the epiphysis (E1–E9) (Figure 3). In older individuals where the medial malleolus was more mature and greater in volume, an additional VOI (E10) was placed within the tip of the medial malleolus (Figure 2). In some individuals, this resulted in an overlap of VOIs E4 and E10. This was taken into consideration during statistical analysis.

DA (star volume distribution method), bone volume fraction, trabecular thickness, and trabecular separation were quantified within each VOI for each individual following Bouxsein et al. (2010). Spherical VOIs were exported into Dragonfly 2022.2 to quantify trabecular numbers.

The appropriateness of the VOI scaling and repeatability of the VOI placement method was tested in a sub‐sample consisting of a fetal, perinatal, 2‐year‐old, 4‐year‐old, 6‐year‐old, and 8‐year‐old individual, resulting in a total of 6 tibiae. The repeatability of the VOI placement method was conducted by a single investigator repeating the placement of VOIs A1–A9 on three separate occasions. DA, BV/TV, Tb.Sp, Tb.Th, and Tb.N were quantified within each VOI. A Friedman test (*α*  = 0.01) was used to assess whether statistically significant differences existed within trabecular parameters between repeats. No statistically significant differences were observed between the 3 repeats for all trabecular parameters (*p* ≥ 0.278).

The appropriateness of VOI scaling was assessed by calculating diametric strut quantity [DSQ (VOI diameter*Tb.N)] and using a minimum DSQ threshold of 4 as proposed by Benjavongkulchai, Davies, and Cunningham (2022). A VOI diameter of 20% anteroposterior distal width of the tibia resulted in a mean DSQ ranging from 4.97–9.08 and thus was deemed an appropriate VOI size for future analysis based upon the > 4 threshold from Benjavongkulchai, Davies, and Cunningham (2022). Volumes of interest were also visually assessed to check at least 3–5 intertrabecular lengths were captured within the VOI as recommended by Harrigan et al. (1988).

### 2.5 Statistical Analysis of VOI Data

Statistical analysis was conducted in SPSS version 28. The following statistical comparisons were conducted for the VOI data:

1. Inter‐developmental group analysis: Trabecular parameters (DA, BV/TV, Tb.Th, Tb.Sp, and Tb.N) for all metaphyseal VOIs were pooled for each developmental group (see Table 1). This data was then compared between developmental groups to give a general overview of trabecular ontogeny.
2. Intra‐developmental group analysis: Within each developmental group, trabecular parameters (DA, BV/TV, Tb.Th, Tb.Sp, and Tb.N) were compared between different VOIs to give an assessment of regional differences during each developmental stage.

Due to the sample size, nonparametric tests were adopted. The distribution of trabecular parameters was visually inspected using boxplots. Some VOIs were absent due to post‐mortem damage to the tibiae, resulting in unequal distributions between VOIs. Therefore, the Kruskal‐Wallis H Test was conducted to compare mean ranks of trabecular parameters (*α*  = 0.01), and if necessary, this was followed by post hoc comparisons using Dunn's (1964) procedure with an SPSS Bonferroni correction for multiple comparisons (*α* = 0.01). SPSS conducts a reverse Bonferroni correction, whereby the p‐value is adjusted by multiplying by the number of comparisons so that the p‐value is comparable to a standard alpha. Alpha was set at 0.01 due to the small sample size.

### 2.6 Whole Bone Mapping

Whole bone trabecular mapping was conducted using Dragonfly 2022.2 (Object Research Systems) following Reznikov et al. (2022). The binarized bones produced during the spherical VOI procedure were exported into Dragonfly 2022.2. Any holes within the segmented bone, such as cortical porosity, were then filled during the first step of the automatic Dragonfly Bone Analysis workflow. Cortical and trabecular bones were then automatically segmented using the Buie et al. (2007) method. A bounding box was then created surrounding the whole distal tibial epiphysis, and the distal 15% of the intermetaphyseal length of the tibiae. Volume fraction and vector fields of anisotropy (magnitude and direction) were then mapped within the bounding boxes. Bone mapping separates the bone within a bounding box into discrete sub‐volumes via a grid. The grid spacing is defined by the step size, whereas the volume of trabeculae to be measured at each grid node is defined by the radius of influence. The average Tb.Th and Tb.Sp data for each development group from the volume of interest results were used to calculate the optimal step size and radius of influence for each developmental group for the tibia and talus respectively (Table 2), whereby:

Sampling=2*Tb.Th

$$\text{Radius of influence}=\left(\mathrm{Tb}.\mathrm{Th}+\mathrm{Tb}.\mathrm{Sp}\right)*2$$

**TABLE 2:** Sampling and radius of influence for each tibial and talar developmental group.

Developmental group	Sampling (mm)	Radius of influence (mm)
Fetal	0.2	0.6
Perinatal	0.3	0.7
Infant and early toddler	0.3	1.1
Early childhood	0.4	1.4

*Note*: Values rounded up to the nearest 0.1 mm.

The radius of influence and sampling sizes were tested for the whole mapping within the distal tibia using the youngest and oldest individuals within the sample. The custom setting was based upon Dragonfly recommendations whereby sampling should be an order of 2*Tb.Th, and the radius of influence was set following Reznikov et al. (2022) who recommend that the radius of influence should not be below the modal size of an average trabecula plus a trabecular pore*1.5. The settings of 5 mm VOI diameter and 2.5 mm step size used by Gross et al. (2014) and parameters used by Reznikov et al. (2022) upon adult calcanei were tested, as was the custom approach tailored by the VOI approach. The method following Gross et al. (2014) resulted in poor detail of the whole bone maps for all individuals, resulting in homogenous areas of BV/TV. Meanwhile, the approach used by Reznikov et al. (2022) resulted in improved detail for the older individuals, but not for the fetal individuals. This was likely due to these methods being designed for adult individuals, and thus when a VOI diameter of 5 mm was placed in fetal and perinatal individuals ranging in distal anteroposterior metaphyseal width of 5–11 mm, this resulted in homogenous results. Anisotropy vector fields following the sampling of both Gross et al. (2014) and Reznikov et al. (2022) resulted in extremely sparse mapping of vectors. While the fetal anisotropy vector fields for the tibia were also sparse following the custom method, they did provide enough detail to evaluate anisotropy. As a result, the custom approach resulted in the best whole bone detail.

Mid‐coronal and mid‐sagittal views of the distal tibia were visualized using the clipping tool within Dragonfly. Volume fraction was color‐coded using a modified version of the Jet look‐up table which was standardized to range from 0 to 1 BV/TV to allow for comparison between individuals. Red coloration therefore indicates approximately > 0.8 anisotropy and > 0.5 BV/TV. The magnitude anisotropy vector field was also mapped using the Jet look‐up table. Direction vector fields were color‐coded as red for the supero‐inferior direction, green for the mediolateral direction, and blue for the anteroposterior direction. Dragonfly appears to incorrectly include pixels outside the bone in the spheres at the bone edge, thus resulting in the low BV/TV observing surrounding every bone within the whole bone maps. The edges of the bone maps were therefore disregarded during the assessment of the internal architecture of the bone. Therefore, instead, the maps were visually assessed using the volume fraction and vector field scales to identify areas of low and high‐volume fraction or anisotropy. Whole bone maps were qualitatively visually assessed to identify patterns in BV/TV and anisotropy during development. No quantitative analyses were conducted on the whole bone maps due to the issues observed within the edges of the trabecular bone.

## 3 Results

### 3.1 Inter‐Development Group VOI Analysis

**FIGURE 4:**
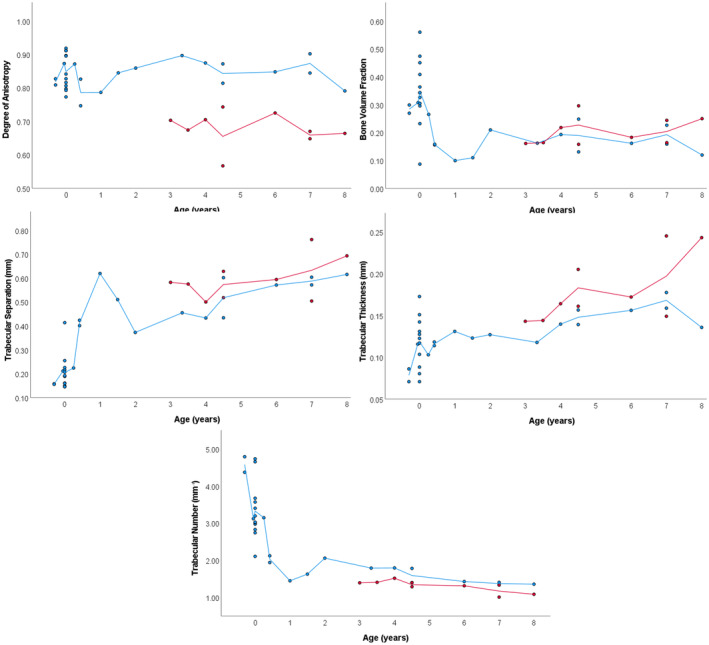
Mean trabecular results from pooled VOI data versus age‐at‐death between 28 weeks intrauterine and 8 postnatal years. Red = distal metaphysis, blue = distal epiphysis. Metaphysis data consists of pooled data from 23 VOIs, distal epiphysis data comprises of pooled data from 10 VOIs.

Age versus mean trabecular parameter scatterplots (Figure 4) indicate dynamic changes in all trabecular parameters during ontogeny. Descriptive statistics and mean ranks for DA, BV/TV, Tb.Sp, Tb.Th, and Tb.N from 23 pooled VOIs within the metaphysis of the fetal, perinatal, and infant and early toddler tibiae are presented in Table 3. Statistically significant differences were observed in the mean ranks of all trabecular parameters (Table 3) between developmental groups following Kruskal‐Wallis tests (Table 4).

**TABLE 3:** Descriptive statistics for the pooled VOI data.

Developmental stage	Statistic	DA	BV/TV	Tb.Sp (mm)	Tb.Th (mm)	Tb.N (mm^−1^)
Fetal (28–38 weeks)	Mean	0.84	0.29	0.17	0.09	4.09
	SD	0.06	0.05	0.04	0.02	0.97
	Mean rank	292.20	417.70	152.75	140.58	554.54
Perinatal	Mean	0.85	0.35	0.20	0.12	3.32
	SD	0.07	0.13	0.08	0.04	0.84
	Mean rank	345.85	470.93	193.83	293.39	461.1
Infant and early toddler (0–2 years)	Mean	0.82	0.16	0.43	0.12	2.06
	SD	0.08	0.07	0.18	0.02	0.66
	Mean rank	278.26	190.33	441.62	301.67	242.92
Early childhood (> 2–8 years)	Mean	0.86	0.18	0.54	0.15	1.54
	SD	0.08	0.07	0.14	0.02	0.33
	Mean rank	373.70	202.98	531.51	491.70	128.93

*Note*: The fetal, perinatal, and infant and early toddler data comprises of pooled data from the 23 VOIs within the metaphysis. The early childhood data comprises of pooled data from 33 VOIs within the metaphysis and the distal epiphysis. Mean ranks were compared between groups using Kruskal‐Wallis test (see results in Table 4).

**TABLE 4:** Kruskal‐wallis and post hoc results comparing mean ranks of pooled VOI data (Table 3) between developmental groups.

Trabecular parameter	Kruskal‐wallis		Dunn's post hoc with SPSS bonferroni adjustment					
	*H*	*p*	Fetal versus perinatal	Fetal versus infant and early toddler	Fetal versus early childhood	Perinatal versus infant and early toddler	Perinatal versus early childhood	Infant and aarly toddler versus early childhood
			*p*	*p*	*p*	*p*	*p*	*p*
DA	23.723	< 0.001*	0.229	1	0.016	< 0.005*	0.770	< 0.0005*
BV/TV	311.823	< 0.0005*	0.240	< 0.0005*	< 0.0005*	< 0.0005*	< 0.0005*	1
Tb.Sp	443.901	< 0.0005*	0.678	< 0.0005*	< 0.0005*	< 0.0005*	< 0.0005*	< 0.0005*
Tb.Th	210.960	< 0.0005*	< 0.0005*	< 0.0005*	< 0.0005*	1	< 0.0005*	< 0.0005*
Tb.N	449.757	< 0.0005*	0.280	< 0.0005*	< 0.0005*	< 0.0005*	< 0.0005*	0.002

*Note*: Fetal = 28–38 weeks, infant and early toddler = 0–2 years, and early childhood = > 2–8 years.

* Statistically significant at *α* = 0.01.

During the fetal period (28–38 weeks intrauterine), the tibia has a high BV/TV and Tb.N, with thin, closely packed trabeculae (Table 3 and Figure 4). There is a statistically significant increase in Tb.Th in the perinatal period in comparison to the fetal period. All other trabecular parameters do not statistically significantly change between these two developmental groups (Tables 3 and 4). During the first year of life, BV/TV, Tb.N, and DA decrease, while Tb.Sp increases, resulting in statistically significant changes in comparison to the perinatal group (Figure 4 and Tables 3 and 4). These parameters then increase after 1 year of age. Trabecular thickness does not have a statistically significant change during this developmental period (Figure 4 and Tables 3 and 4). In the early childhood group (2–8 years), Tb.Sp and Tb.Th increase, while Tb.N decreases, resulting in statistically significant differences in this group in comparison to the infant and early toddler group (Figure 4 and Tables 3 and 4). There was no statistically significant change in DA and BV/TV, in comparison to the infant and early toddler group (Figure 4 and Tables 3 and 4).

### 3.2 Intra‐Developmental Group VOI Analysis

To investigate regional differences in trabecular parameters within the distal tibia, Kruskal‐Wallis tests were used to compare trabecular parameters between multiple VOIs for each developmental group (Table 5). See Supporting Information for mean ranks, and minimum and maximum descriptive statistics for each VOI at each developmental stage (Tables S1–S4).

**TABLE 5:** Kruskal‐wallis results comparing mean ranks of trabecular parameters between VOIs. See Supporting Information for descriptive statistics and means ranks of each VOI.

Developmental stage	Statistic	DA	BV/TV	Tb.Sp	Tb.Th	Tb.N
Fetal	df	22	22	22	22	22
	H	16.545	28.415	17.805	30.389	23.589
	*p*	0.788	0.162	0.717	0.109	0.369
Perinatal	df	22	22	22	22	22
	H	20.920	17.090	29.908	68.710	58.693
	*p*	0.526	0.758	0.121	< 0.001* ^a^	< 0.001* ^a^
Infancy and early toddler	df	22	22	22	22	22
	H	17.147	26.522	46.330	46.205	22
	*p*	0.755	0.230	< 0.002* ^a^	< 0.002* ^a^	< 0.001* ^a^
Early childhood	df	32	32	32	32	32
	H	158.407	105.314	176.133	47.021	168.002
	*p*	< 0.001*	< 0.001*	< 0.001*	0.042	< 0.001*

^a^ Statistically insignificant post hoc comparisons.

* Statistically significant difference.

### 3.3 Fetal (28–38 Weeks Intrauterine)

Volume of interest results indicate a trend that BV/TV, Tb.Th, and Tb.Sp increase in a distal (row A VOIs) to the proximal direction (row C VOIs) in the fetal tibial metaphysis, while Tb.N acts inversely (Table S1). However, no statistically significant differences were observed between VOIs for any trabecular parameters indicating a lack of distinct differences in trabecular structure within different regions of the distal tibia (Table 5). Overall, the fetal distal tibia is anisotropic, with homogeneity of VOIs (Table S1).

### 3.4 Perinatal

Similar to the fetal results, the perinatal tibia displayed a trend for greater BV/TV, Tb.Th, and Tb.Sp within the more proximally located VOIs in comparison to the more distally located VOIs, while Tb.N behaved inversely (Table S2). These trends were not statistically significant (Table 5). DA was also homogenous throughout the distal tibia, with no statistically significant differences being observed between VOIs (Table 5 and Table S2).

### 3.5 Infant and Early Toddler (0–2 Years)

Within the first two years of postnatal life, in the infant and early toddler group, no statistically significant differences were observed for any trabecular parameters between VOIs (Table 5). DA, Tb.Sp, Tb.Th and Tb.N shared the same trends as the fetal and perinatal tibia (Table S3). There was, however, a shift in BV/TV gradient observed in fetal and perinatal tibiae. Bone volume fraction was lower proximally and within the center of the metaphysis compared to the distal‐most aspect of the metaphysis (Table S3).

### 3.6 Early Childhood (2–8 Years)

After 2 years of age, in the early childhood group, the distal tibia exhibits a substantial increase in structural heterogeneity with DA, BV/TV, Tb.Sp, and Tb.N having statistically significant differences between groups (Table 5). The center of the metaphysis has a lower BV/TV, Tb.N, and a higher Tb.Sp than the peripheries of the metaphysis (Table 6 and Table S4). The trabecular number is also higher in the distal‐most aspect of the metaphysis in comparison to the proximal metaphysis and epiphysis (Table 6 and Table S4). The metaphysis is highly anisotropic, while the distal epiphysis, in particular the medial malleolus, is more isotropic (Table 6). Trabecular thickness was relatively homogeneous throughout the metaphasis, with the epiphysis containing thicker trabeculae (Table S4). However, this was not statistically significant (Table 5).

**TABLE 6:** Statistically significant (*p* < 0.01) post hoc dunn's comparisons with a SPSS Bonferroni correction between early childhood distal tibial volumes of interest.

Trabecular separation		Trabecular number		Degree of anisotropy		Bone volume fraction	
Pairwise VOI comparison	Adjusted *p*	Pairwise VOI comparison	Adjusted *p*	Pairwise VOI comparison	Adjusted *p*	Pairwise VOI comparison	Adjusted *p*
A7‐C2	0.007	C1‐A2	0.004	E2‐A2	0.004	C1‐E2	0.009
A7‐C5	0.003	C1‐A3	0.003	E2‐B8	0.002	C1‐B7	0.007
A7‐C3	0.002	C1‐A9	0.001	E2‐A8	0.001	C1‐A2	0.005
A7‐E4	0.001	C1‐A8	< 0.0005	E2‐A1	< 0.0005	C1‐E3	0.003
A7‐E1	< 0.0005	C1‐A5	< 0.0005	E2‐A5	< 0.0005	C1‐A8	0.002
A7‐B1	< 0.0005	C1‐A6	< 0.0005	E10‐B8	0.005	C1‐E5	0.001
A7‐C1	< 0.0005	C1‐A4	< 0.0005	E10‐A8	0.002	C1‐E9	0.001
A4‐C5	0.005	C1‐A7	< 0.0005	E10‐A1	< 0.0005	C1‐E8	< 0.0005
A4‐C3	0.003	E1‐A9	0.004	E10‐A5	< 0.0005	C1‐A6	< 0.0005
A4‐E4	0.002	E1‐A8	0.001	E3‐B8	0.008	C1‐A4	< 0.0005
A4‐E1	0.001	E1‐A5	< 0.0005	E3‐A8	0.004	C1‐A7	< 0.0005
A4‐B1	< 0.0005	E1‐A6	< 0.0005	E3‐A1	0.001	B1‐E8	0.005
A4‐C1	< 0.0005	E1‐A4	< 0.0005	E3‐A5	< 0.0005	B1‐A6	0.003
A6‐C5	0.005	E1‐A7	< 0.0005	E6‐A1	0.004	B1‐A4	0.002
A6‐C3	0.003	B1‐A8	0.004	E6‐A5	< 0.0005	B1‐A7	0.001
A6‐E4	0.002	B1‐A5	0.004	E8‐A1	0.005		
A6‐E1	0.001	B1‐A6	0.003	E8‐A5	< 0.0005		
A6‐B1	< 0.0005	B1‐A4	0.002	E4‐A5	0.002		
A6‐C1	< 0.0005	B1‐A7	0.001	E1‐A5	0.003		
A5‐C5	0.006	C3‐A8	0.01	E5‐A5	0.004		
A5‐C3	0.004	C3‐A5	0.009	E9‐A5	0.009		
A5‐E4	0.003	C3‐A6	0.006				
A5‐E1	0.001	C3‐A4	0.005				
A5‐B1	< 0.0005	C3‐A7	0.003				
A5‐C1	< 0.0005	E4‐A6	0.008				
A8‐C5	0.008	E4‐A4	0.006				
A8‐C3	0.005	E4‐A7	0.003				
A8‐E4	0.004	C2‐A7	0.007				
A8‐E1	0.001	C5‐A7	0.007				
A8‐B1	< 0.0005						
A8‐C1	< 0.0005						
A9‐E1	0.01						
A9‐B1	0.003						
A9‐C1	< 0.0005						
A3‐C1	0.001						
A2‐C1	0.001						

*Note*: See Figure 1 for location of each volume of interest.

### 3.7 Whole Bone Mapping

Whole bone maps for each individual are presented in (Figures S1–S18).

### 3.8 Fetal

**FIGURE 5:**
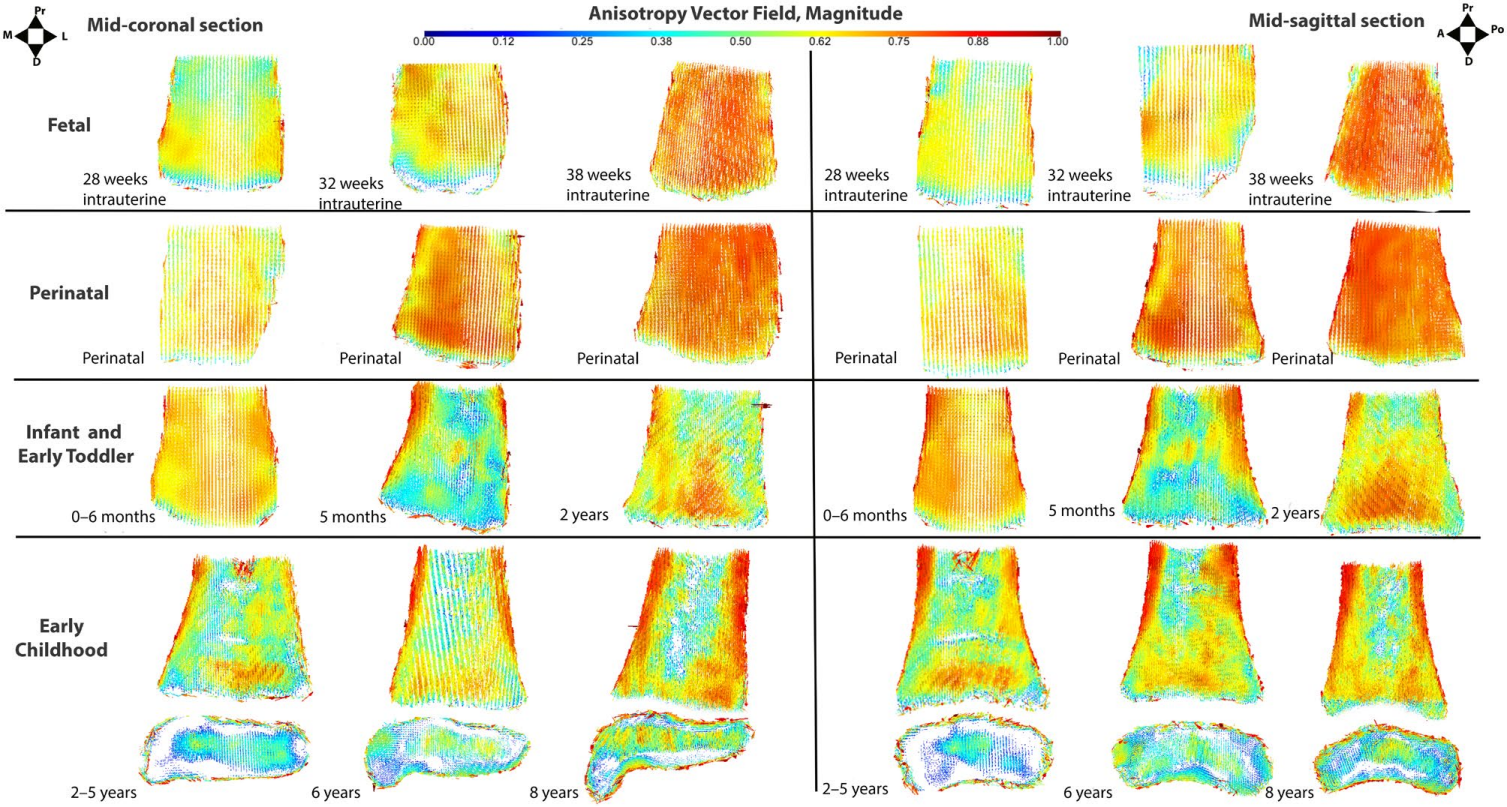
Mid‐coronal (left) and mid‐sagittal (right) anisotropy vector fields, magnitude. For each view, left column = youngest, center column = median, and right column = oldest individual in group. A = anterior, D = distal, L = lateral, M = medial, Po = posterior and Pr = proximal. Estimated age‐at‐death ranges reported, except for documented individuals. Anisotropy increases between the fetal and perinatal period, indicated by the increased presence of red depicting high DA values (> 0.75). Postnatally, the tibia appears to have greater heterogeneity, illustrated by the greater variety of colors.

**FIGURE 6:**
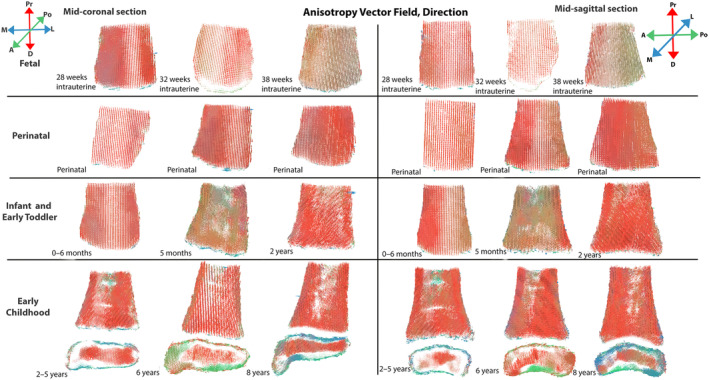
Mid‐coronal (left) and mid‐sagittal (right) anisotropy vector fields, direction. For each view, left column = youngest, center column = median, and right column = oldest individual in group. A = anterior, D = distal, L = lateral, M = medial, Po = posterior and Pr = proximal. Estimated age‐at‐death ranges reported, except for documented individuals. Metaphyseal trabeculae appear consistently aligned in the superoinferior direction between 28 weeks intrauterine and 8 postnatal years, while the distal epiphysis appears more complex, with the medial malleolus demonstrating trabeculae aligned in multiple directions.

**FIGURE 7:**
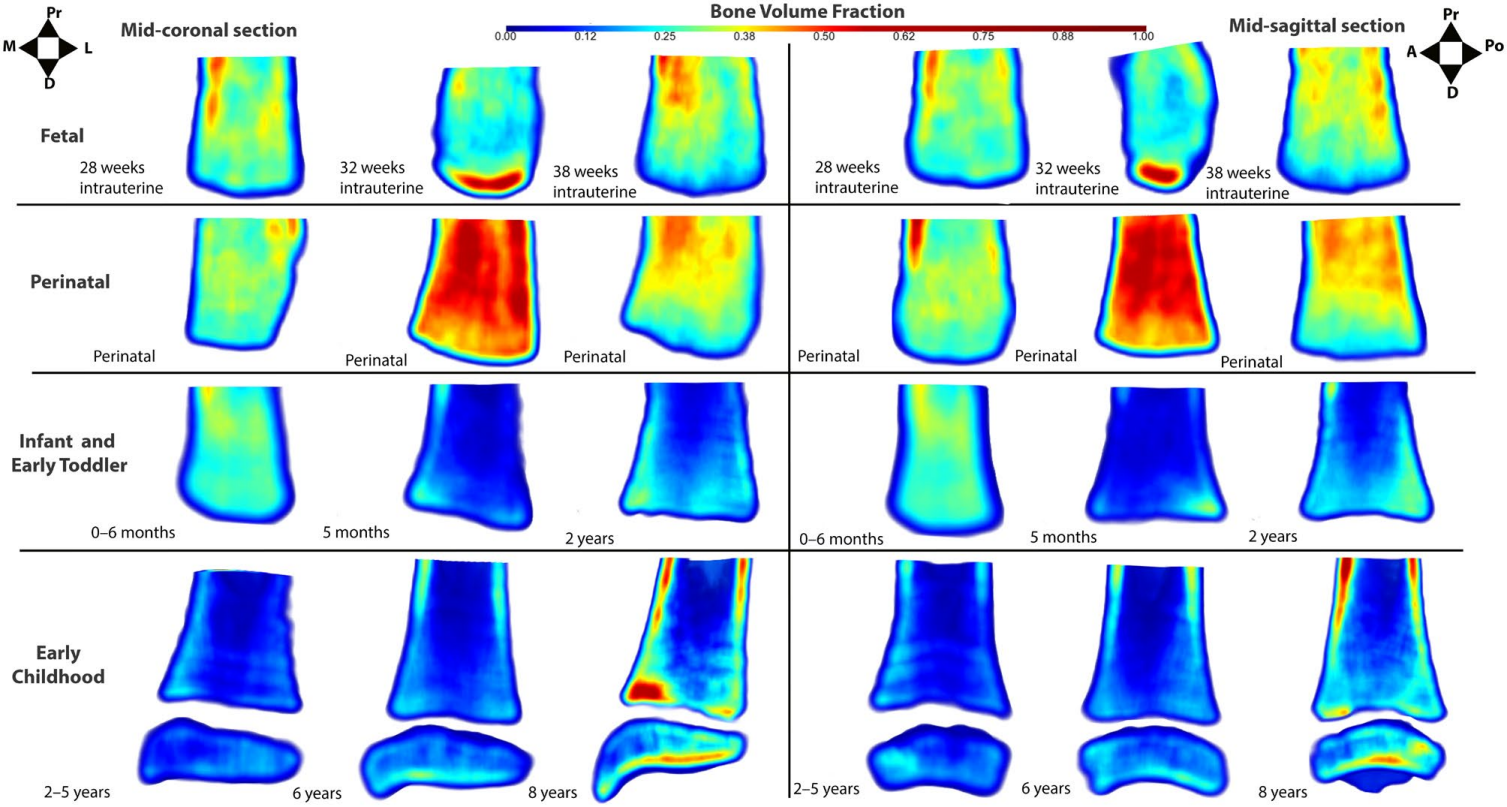
Mid‐coronal (left) and mid‐sagittal (right) bone volume fraction maps. For each view, left column = youngest, center column = median, and right column = oldest individual in group. A = anterior, D = distal, L = lateral, M = medial, Po = posterior and Pr = proximal estimated age‐at‐death ranges reported, except for documented individuals. Bone volume fraction appears greatest during the fetal and perinatal period, before decreasing within the infant and early toddler group. From 2 years elevated BV/TV trajectories are apparent extending down the peripheries of the metaphysis.

Anisotropy appears to increase throughout the fetal period with the presence of red (indicating high anisotropy values) increasing from 28 weeks through to 38 weeks (Figure 5), while trabeculae are aligned in the superoinferior direction (Figure 6). Bone volume fraction appears to increase in a distal to proximal direction in the fetal tibial metaphysis (Figure 7).

### 3.9 Perinatal

Whole bone mapping suggests a lack of consistent organization within the perinatal tibia, but with trabeculae predominately aligned in the supero‐inferior direction (Figures 5, 6). Perinatal BV/TV maps demonstrate considerable variation in BV/TV between individuals (Figure 7). Some bones appear to have homogenous high BV/TV values (Figure 7) while others have homogenous low BV/TV values (Figures S8 and S9). Some perinatal individuals appear to have a decrease in BV/TV from the proximal to distal direction (Figure 7).

### 3.10 Infant and Early Toddler

**FIGURE 8:**
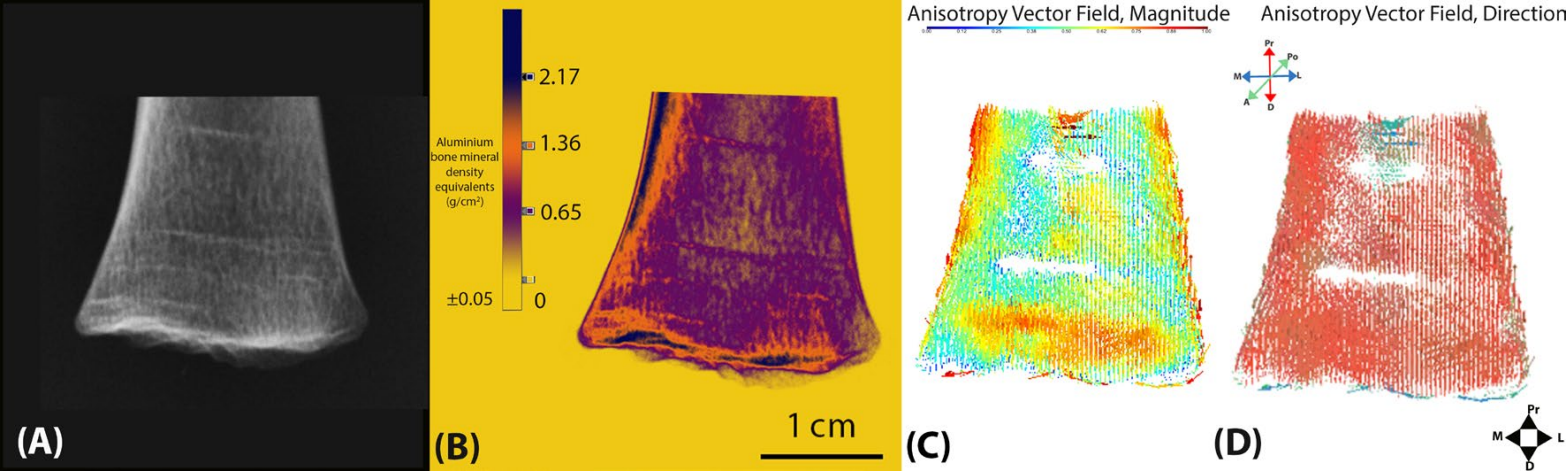
Identification of Harris lines in a left distal tibia (A) raw radiograph, (B) radiographic color map from Reid, Davies, and Cunningham (2023), (C) anisotropy vector field, magnitude, and (D) anisotropy vector field, direction. Sparse areas in vector fields coincide with Harris lines in radiographs.

Whole bone mapping indicates the tibia becomes more organized within the infant and early toddler group (Figures 5, 6, 7). In the youngest individual of the group, there does not appear to be any specific pattern in the magnitude of anisotropy (Figure 5). In the most mature individual of the group at 2 years of age, moderate anisotropic bands appear from the medial, lateral, anterior, and posterior boundaries of the tibia and extend distally (Figure 5). This begins to appear after 1 year of age. The direction of trabeculae appears uniform in the vertical direction, except for transversely aligned sparse areas in some individuals (Figure 8D,E). Sparse areas in vector fields may appear when trabeculae are aligned in multiple different orientations (Reznikov et al. 2022), and coincide with where Harris lines were identified radiographically (Figure 8A,B).

Bone volume fraction maps suggest BV/TV is low and relatively homogenous in the infant and early toddler distal tibia (Figure 7). The greater presence of dark blue within this group indicates BV/TV decreases in comparison to the perinatal group (Figure 7). At the extreme ends of the group, BV/TV patterns differ from this. The youngest individual appears to have high BV/TV that is slightly greater in BV/TV in the proximal aspect of the bone in comparison to the distal area (Figure 7). In the oldest individual in the group, there appears to be greater heterogeneity in BV/TV, with the highest BV/TV extending distally from the peripheries of the tibia and into the distal‐most region of the bone (Figure 7).

### 3.11 Early Childhood

Within the early childhood vector fields, anisotropic bands are apparent extending down from the peripheries of the metaphysis toward the most distal aspect of the metaphysis (Figure 5J–L). These trajectories become more distinctive with maturation in this group. Trabeculae appears to be orientated in a uniform vertical direction in the metaphysis, except for sparse areas in some tibiae that coincide with Harris lines (Figure 6).

The direction of trabeculae in the early childhood distal epiphysis appears more complex (Figure 6). Vertically aligned and anisotropic trabeculae are observed within the distal epiphysis (Figure 6). The shoulder of the medial malleolus becomes increasingly complex, from being horizontally orientated in the mediolateral direction (Figure 6), to a combination of anteroposteriorly and mediolaterally orientated (Figure 6). The tip of the medial malleolus is complexly organized with trabeculae in several directions in more mature individuals of this group, from approximately 6 years of age (Figure 6). The medial malleolus appears more isotropic than the rest of the distal tibia in more mature individuals (Figure 5).

## 4 Discussion

Microcomputed tomography of the distal tibia between 28 intrauterine weeks and 8 postnatal years demonstrated dynamic changes in the trabecular architecture throughout ontogeny. Our hypotheses were mostly retained. The trabecular organization of the pre‐loading distal tibia is dictated by endochondral ossification and gestational overproduction (Acquaah et al. 2015). Constructive regression was observed during the first year of life (Acquaah et al. 2015). After 1 year of life, structural heterogeneity of the distal tibiae increased and became refined in line with the acquisition and maturation of the bipedal gait (Acquaah et al. 2015; Saers et al. 2022a, 2022b). However, by 8 years of age, the distal tibia did not exhibit high BV/TV within the medial and lateral regions as observed within adults (Du et al. 2019; Sode et al. 2010), suggesting that refinement of the trabecular architecture is not complete by 8 years.

### 4.1 Fetal and Perinatal Period: Gestational Overproduction and Ossification Patterns

During the fetal period, VOI analysis of the tibia indicates high BV/TV and Tb.N, with thin, closely packed trabeculae, consistent with other regions of the fetal skeleton (Table S1) (Acquaah et al. 2015; Nuzzo et al. 2003; Salle et al. 2002). This generic arrangement of trabeculae throughout the skeleton is postulated to present a large surface area for osteoclastic activity to release mineral content necessary for postnatal growth and development. Given the prevalence of this trabecular arrangement throughout the skeleton, it is hypothesized that this is a genetically controlled trabecular blueprint.

As a result of gestational overproduction, BV/TV is at its highest during the perinatal period (Figure 4 and Table 3). This is driven by an increase in trabecular thickness, while Tb.N decreases. These two trabecular parameters appear to behave differently in other skeletal sites in the fetal and perinatal skeletons. Trabecular thickness decreases, while Tb.N remains constant in the proximal femur (Salle et al. 2002). In comparison, Tb.Th appears to increase then decrease in vertebrae, while Tb.N behaves inversely to thickness (Acquaah et al. 2015). Nuzzo et al. (2003), however, states Tb.Th remains constant within the lumbar vertebrae. It may be that our small fetal sample size does not reflect the complexities in Tb.N and Tb.Th changes, as our results agree initially with Acquaah et al. (2015). Conversely, it may be that an increase in BV/TV during the fetal period is driven by a variety of trabecular changes throughout the skeleton.

Structural heterogeneity analysis suggests that the fetal and perinatal distal tibia lacks a mature organization (Figures 5, 6, 7 and Tables S1 and S2) but instead reflects the ossification pattern of the bone, in agreement with our previous radiographic results (Reid, Davies, and Cunningham 2023). Although not statistically significant, there is a trend for decreasing BV/TV, Tb.Th, and Tb.Sp, while Tb.N increases with distance from the primary ossification center within the mid‐shaft of the fetal and perinatal tibia (Tables S1 and S2). This gradient along the metaphyses of bones in the fetal and perinatal tibia is postulated to be due to the progression of ossification. The bone closest to the primary ossification center of the tibia, located in the mid‐shaft of the bone, hypothetically will have the oldest bone which therefore has more time for modeling and thus will be greater in BV/TV and Tb.Th than the most distal aspect of the bone. Gradients of trabecular architecture have previously been observed within the neonatal ilium and are proposed to be related to ‘metaphyseal drivers’ (Cunningham and Black 2010). A similar pattern has also been observed during the early development of the proximal femur (Ryan and Krovitz 2006). Before 4 months of age, the superior aspect of the proximal femur also had a lower BV/TV and Tb.Th, with higher Tb.N than a VOI placed more distally within the proximal femur, closer to the primary ossification center (Ryan and Krovitz 2006).

### 4.2 Infant and Early Childhood: Constructive Regression and Early Refinement

Results indicate that the BV/TV decrease occurs during the first year of life and begins to increase again after 1 year of age (Figure 4). This is consistent with developmental patterns in other weight‐bearing bones (Acquaah et al. 2015; Figus, Stephens, et al. 2023; Gosman and Ketcham 2009; Milovanovic et al. 2017; Ryan and Krovitz 2006; Ryan, Raichlen, and Gosman 2017; Saers, Ryan, and Stock 2019). The decrease in BV/TV in the distal tibia is driven by a decrease in Tb.N and an increase in Tb.Sp, while Tb.Th does not change in comparison to the perinatal stage. A decrease in Tb.N has also been observed in other regions of the skeleton (Acquaah et al. 2015; Gosman and Ketcham 2009; Maclean 2017; Milovanovic et al. 2017; Ryan and Krovitz 2006; Saers, Ryan, and Stock 2019). An increase in Tb.Th has been observed in the femur (Milovanovic et al. 2017; Ryan and Krovitz 2006), the calcaneus (Saers, Ryan, and Stock 2019), and the ischium (Maclean 2017). Our statistical approach may have averaged out differences in Tb.Th during this stage, as there is a trend for increasing Tb.Th between the oldest and youngest individuals of this group (Figure 4).

It has been postulated that this decrease in BV/TV is related to (re)modeling to accommodate newly applied biomechanical forces associated with the attainment of motor milestones (Acquaah et al. 2015; Milovanovic et al. 2017). Underloaded trabeculae may be resorbed at this stage, reflected by the decrease in Tb.N, while the remaining trabeculae increase in separation, possibly aligning themselves to specific loads.

There are indications of the onset of re‐organization of the distal tibia during the first two years of life, which signals an adaptation to bipedal loading. While the trabecular organization still appears to be linked to ossification patterns (with higher Tb.Sp and Tb.Th and lower Tb.N closer to the primary ossification center, in comparison to the distal‐most aspect), there is an inverse BV/TV pattern in comparison to the fetal and perinatal tibia (Figure 7 and Tables S1–S3). In the fetal and perinatal tibia, BV/TV was highest most proximally within the metaphysis, but in the infant and early toddler group, there was a trend for lower BV/TV proximally and within the center of the metaphysis (VOI B1 and C1) in comparison to the distal‐most aspect of the metaphysis (SI Tables S1–S3). This indicates that changes to bone laid down during the fetal and perinatal period may have occurred.

Whole bone mapping also indicates that the tibia becomes more organized within the infant and early toddler group, with maps after 1 year of age exhibiting anisotropic trabecular trajectories extending down the medial, lateral, anterior, and posterior aspects of the tibia, and converging in the distal‐most aspect of the metaphysis (Figure 5). This appears to be associated with the ‘longitudinal trajectories’ identified within radiographic color maps (Reid, Davies, and Cunningham 2023). This organization is consistent with the trabecular architecture in the inner region of the adult distal tibia displaying lower BV/TV and Tb.N with higher Tb.Sp than the outer region of the bone (Sode et al. 2010). The whole bone mapping indicates this structure appears after 1 year, which may be linked to the acquisition of the bipedal gait during this stage (Figure S9). No observations based upon the attainment and maturation of motor milestones such as crawling and cruising were made, due to the small sample sizes documenting these changes, rather than interpretation focused on post‐loading once the bipedal gait had developed. The early organization of the distal tibia prior to 1 year of age, is therefore consistent with the ‘pre‐locomotor phase’ of the model proposed by Saers et al. (2022a, 2022b) whereby gestational overproduction occurs with an ossification‐dictated organization of bone followed by bone resorption during constructive regression (Saers et al. 2022b).

Increased inter‐individual variation in DA during the attainment of the bipedal gait has previously been observed (Raichlen et al. 2015), however, in our study, no distinct differences in the standard deviations of DA were observed between different developmental groups (Table 3). This may be due to our small sample within the infant and early toddler group and different methodological approaches.

### 4.3 Early Childhood: Refinement

Within the early childhood group, the distal tibia exhibits a substantial increase in structural heterogeneity. The volume of interest results demonstrates the typical trabecular organization of individuals within this group, while mapping illustrates how these bones become increasingly more adapted to the maturing bipedal gait. The tibia therefore does not instantly develop to its adult state. This is consistent with ‘refinement’ observed within other regions of the skeleton, with trabeculae becoming adapted to their different local functional requirements (Acquaah et al. 2015; Milovanovic et al. 2017; Ryan, Raichlen, and Gosman 2017; Ryan and Krovitz 2006; Saers, Ryan, and Stock 2019). This is also aligned with the continuation of the ‘neuromaturation phase’ described by Saers et al. (2022b), whereby a re‐organization of trabeculae occurs between the onset of locomotion and the achievement of adult gait.

The statistically significant differences in the VOI results indicate that the center of the tibial metaphysis has a lower BV/TV, Tb.N, and a higher Tb.Sp than the peripheries of the metaphysis (Table 6 and Table S4), in agreement with the results of Sode et al. (2010). This relates to the anisotropic bands observed in the whole bone maps extending down the medial, lateral, anterior, and posterior boundaries for the metaphysis and converging at the most‐distal aspect of the metaphysis. These are also consistent with the structures identified radiographically and termed as the ‘longitudinal trajectories’ (Reid, Davies, and Cunningham 2023). These anisotropic bands likely facilitate compressive bodyweight forces through the tibia and into the talus via the talocrural joint. These anisotropic bands become increasingly distinct within the whole bone maps, indicating these develop as gait matures. Du et al. (2019) and Sode et al. (2010) observed high BV/TV and Tb.Th within the posterior and medial region of the tibia to accommodate shearing and compressive forces. However, no statistically significant differences in Tb.Th and BV.TV were observed between these regions and the anterior and lateral aspects of the distal metaphysis (Table 6). It could be that these differences arise after 8 years of age, as other regions of the ankle joint have demonstrated trabecular changes after 8 years, suggesting refinement is not complete at 8 years (Figus, Sorrentino, et al. 2023; Goliath et al. 2022).

The distal epiphysis appears more complex than the metaphysis. In agreement with Takechi et al. (1982), thick trabeculae were observed in the distal epiphysis (Table S4), superior to its articulation with the trochlea of the talus, consistent with the radiographically identified ‘distal articular’ trajectory (Reid, Davies, and Cunningham 2023). After 4 years of age, whole bone maps indicate that the distal epiphysis has high BV/TV at its articulation with the trochlea of the talus (Figure 7). These trabeculae are likely involved in the transmission of compressive body weight forces, which pass down through the anisotropic bands within the metaphysis and into the distal epiphysis. The distal epiphysis is more isotropic than the remainder of the distal tibia, indicated by the statistically significant differences between multiple epiphyseal VOIs versus metaphyseal VOIs (Table 6 and Table S4). The anisotropy vector fields indicate that the medial malleolus is isotropic (Figure 5). This is reflected in the directionality of the anisotropy vector fields, with trabeculae aligned in several directions, including the mediolateral direction (Figure 6). These multi‐directional trabeculae become clear around 6 years of age within the vector fields (Figures S14 and S15). Overall, this agrees with Takechi et al. (1982) who stated the trabeculae nearest the talar facet within the adult medial malleolus were transversely orientated to facilitate tensile forces associated with the collateral ligaments and with the radiographic study (Reid, Davies, and Cunningham 2023).

### 4.4 Methodological Considerations

The grouping of the VOI results into different age cohorts had implications upon the μCT statistical comparisons. From the age versus trabecular parameters scatterplots and whole bone maps, it was clear that after 1 year of age, the trend of decreasing BV/TV starts to reverse within the distal tibia and talus as they start to adapt to locomotive forces. As such, the VOI results average out these changes between 0 and 1 year and do not clearly illustrate how structural heterogeneity gradually develops with maturity. This criticism is not restricted to just to the infant and early toddler group, but the entire VOI analysis using developmental groups. As such, the VOI results demonstrate the typical trabecular structure of the developmental group. The whole bone maps and age versus trabecular parameter scatterplots were therefore useful accompaniments to visualize the gradual development of the distal tibia and talus.

The impact of the methodology on the trabecular analysis must be considered. The quantification of trabecular parameters using the volume of interest approach can be impacted by the size and placement of the VOIs (Benjavongkulchai, Davies, and Cunningham 2022; Kivell et al. 2011; Lazenby et al. 2011). Although no statistically significant differences occurred in trabecular parameters between 3 repeats of placement of VOIs A1–3, it is acknowledged, however, that inter‐observer error in the placement of VOIs may exist. It should also be noted that providing a mean trabecular parameter from pooled data from multiple VOIs was used to give a general overview of changes throughout ontogeny and does not provide insight into structural variation within the distal tibia. This approach has been used previously by Goliath et al. (2022) and Raichlen et al. (2015) to provide an overview of changes within the proximal tibia. To alleviate some of the limitations of the VOI approach, including the difficulty in identifying homologous areas at different ages, whole bone mapping was also utilized for analysis. There was agreement between the whole bone maps and the statistical results of the VOI approach.

Most scans were conducted as part of a separate project therefore no refinement of scan resolution was possible. It is possible that the resolution did not capture small trabeculae. However, Bouxsein et al. (2010) recommend that trabeculae should be represented by a minimum of 3–4 voxels utilized as guidance for resolution. Colombo et al. (2021) calculated the minimum suitable resolution for the ontogenetic study of the proximal humerus using Tb.Th data from the literature whereby $\text{resolution}=\frac{\mathrm{Tb}.\mathrm{Th}}{3}$. Mean trabecular thickness in the proximal juvenile tibia between 0.1 and 9.8 years was documented by Gosman and Ketcham (2009) as 67–190 μm, suggesting a minimum resolution of 22.33–63.33 μm for the juvenile tibia. The lower value of these ranges indicates the minimum resolution for the youngest individuals within the sample, while the upper value indicates the minimum resolution for the older individuals. Therefore, the scanning resolution employed in this study appears adequate based on these recommendations.

The scaling of VOIs within the distal tibia was initially conducted based on the recommendations of Colombo et al. (2021) that at least 3% juvenile long bone length was required for scaling a VOI. It has been argued that VOIs should be scaled to a percentage of diaphyseal bone length because the growth of long bones is isometric (Saers et al. 2022b; Stern et al. 2015). The initial 3% bone length was adapted to use a measure of anteroposterior width as a scaling measurement to ensure that VOIs could fit within a cross‐section of the distal tibia. The correlation between the diaphyseal length of the tibia and the maximum anteroposterior width of the talus was assessed via Spearman Rank order correlation. The relationship between these pairs of measurements was also assessed using Huxley's allometric equation: logy=logb+klogx, where *y* = mass of a body part, *x* = total body mass, and *k* = allometric coefficient (Niklas, Hammond, and Friedman 2014). This method consists of fitting a straight line to logarithmic transformations of measurements and the intercept and slope of these lines are then interpreted (Niklas, Hammond, and Friedman 2014). The allometric coefficient (*k*, also denoted by α) is utilized for analysis, where values of 1 demonstrate perfect isometry, +1 demonstrates a hyperallometric relationship, and −1 demonstrates a hypoallometric relationship (Shingleton, Mirth, and Bates 2008). Diaphyseal length demonstrated a slightly hypoallometric relationship with the AP width of the metaphysis (*α* = 0.94), and a strong correlation (*r*_*s*_ = 0.897, *p* < 0.001). This indicates that using the AP width of the distal tibia may be an appropriate substitute for scaling based on diaphyseal length. The anteroposterior width of long bones has previously been employed in ontogenetic studies of the humerus and tibia (Goliath et al. 2022; Perchalski et al. 2018).

To overcome some of the limitations associated with the use of a VOI strategy, whole bone mapping was adopted following Reznikov et al. (2022). A custom sampling method was used to accommodate for the changing size of the tibia during ontogeny, as using a global sampling method resulted in the under‐sampling of the younger individuals. Empty areas in the anisotropy vector fields were observed within the fetal and perinatal tibial maps which may be a limitation of the custom sampling approach or may be due to trabeculae in multiple different directions canceling each other out (Reznikov et al. 2022). The selected ‘custom’ approach was based upon the VOI results and the recommendation from Dragonfly and Reznikov et al. (2022) and ensured that a consistent sampling approach for mapping was used throughout the project. To date, no guidelines exist for the whole bone mapping approach to sampling for juvenile individuals, therefore this should be explored further in future research.

The incorrect quantification of BV/TV at the trabecular edges of the tibiae by Dragonfly is a limitation of this study. Given that subchondral bone demonstrates adaptation to joint loading, failure to capture the distal‐most trabeculae within the tibia potentially omits visualization of locomotor signals within the tibia (Goliath et al. 2022). The anisotropy vector fields of some tibial individuals within the infant and early toddler group and early childhood group have sparse areas (Figures 5 and 6). In areas of vector fields where vectors are arranged in several different directions, the vectors may cancel each other out and result in blank areas (Reznikov et al. 2022). When compared to the radiographs of the same individuals and the previous radiographic color maps, these sparse areas coincide with Harris lines (Reid, Davies, and Cunningham 2023). Despite the limitations of the whole bone mapping used in this study, overall, whole bone mapping presented a useful tool for visualizing entire bone trabecular architecture, in accompaniment to VOI results. In particular, the anisotropy vector field directionality maps presented additional information that could not be gleaned from more traditional DA measurements. Previous measures of DA, such as the VOI results, provide information as to whether trabeculae were organized or disorganized, but provide no information as to which direction the trabeculae were ‘organized’. The direction maps were enlightening to depict the complex arrangement of trabeculae in the distal tibial epiphysis. Whole bone mapping therefore complemented the VOI approach and provided insight when it was questionable whether spherical VOIs are truly representative of the complexities of trabecular structure.

Due to the paucity of juvenile skeletal material, this study is limited by a small sample size, with the fetal infant, and early childhood groups being under‐represented. Similarly, our sample is comprised of archaeological and historical anatomical individuals, with most tibiae having an estimated age at death and unknown cause of death, pathology, and socio‐economic status. It is therefore not possible to assess how these factors may impact our results, and how applicable they are to modern populations.

## 5 Conclusions

Trabecular analysis of the distal tibiae between 28 intrauterine weeks and 8 postnatal years, via a volume of interest approach and whole bone mapping, revealed ontogenetic patterns previously reported in other regions of the trabecular skeleton (Figus et al. 2022; Figus, Sorrentino, et al. 2023; Figus, Stephens, et al. 2023; Gosman and Ketcham 2009; Milovanovic et al. 2017; Ryan and Krovitz 2006; Ryan, Raichlen, and Gosman 2017; Saers, Ryan, and Stock 2019). The fetal tibia demonstrated trabecular architecture that is proposed to be a genetically controlled generic trabecular template. There was an accumulation of bone mass during the fetal period, resulting in high BV/TV during the fetal and perinatal stage. Within these developmental stages, the distal tibia lacks a mature organization and instead, its internal structure appears related to the ossification pattern of the tibia. During the first year of life, constructive regression occurs which may be in response to the biomechanical changes associated with the acquisition of bipedal gait, in addition to growth demands. After 1 year of age, the distal tibia exhibits increased structural heterogeneity associated with the adaptation of trabecular bone to facilitate the dispersion of bipedal stresses. Overall, this study provides insight into the trabecular development of the distal tibia.

## Author Contributions

**Rebecca A. G. Reid:** conceptualization (lead), formal analysis (lead), investigation (lead), methodology (lead), project administration (lead), visualization (lead), writing – original draft (lead), writing – review and editing (lead). **Catriona Davies:** conceptualization (equal), project administration (equal), supervision (equal), writing – original draft (equal), writing – review and editing (equal). **Craig Cunningham:** conceptualization (equal), project administration (equal), supervision (equal), writing – original draft (equal), writing – review and editing (equal).

## Supporting information


Data S1.

Video S1.

**Video S2** Image.

Video S3.

**Video S4.** Image.

## Data Availability

The data that support the findings of this study are available upon reasonable request by writing to the Center Manager of the Center for Anatomy and Human Identification.
