# Supplementary material for: Trabecular Bone Ontogeny of the Human Distal Tibia
Source: Am J Biol Anthropol. 2024 Dec 8;186(1):e25043. doi: 10.1002/ajpa.25043 (PMC11775436; doi:10.1002/ajpa.25043)
Supplement: Supplementary file 1 — Data S1. [file AJPA-186-e25043-s004.pdf]

Supplementary Material

Whole Bone Maps

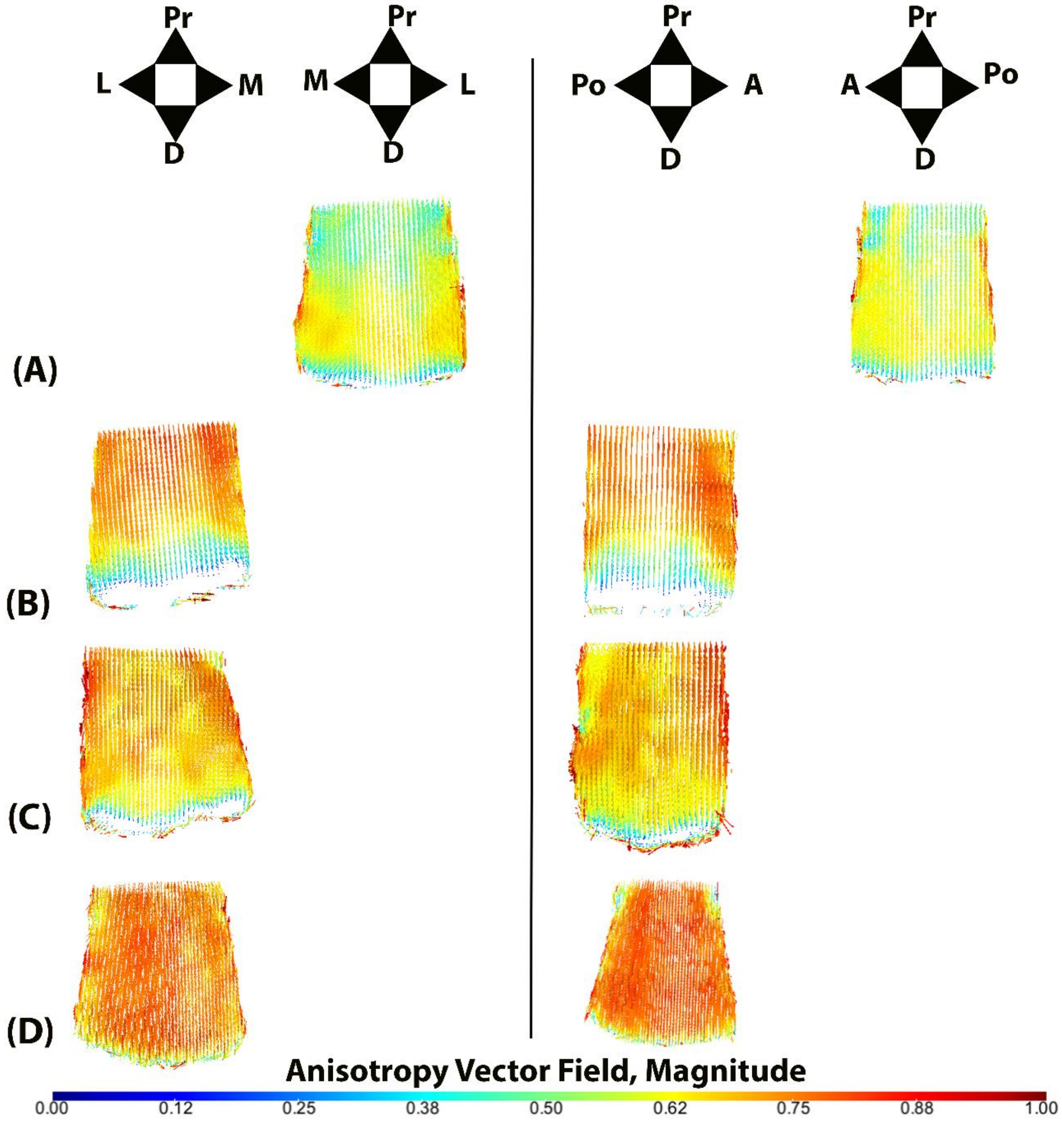

Figure 1: Fetal Anisotropy Vector Field, Magnitude Mapping. 28 weeks intrauterine, B) 28 weeks intrauterine, C) 32 weeks intrauterine, D) 38 weeks intrauterine. Left column =mid-coronal view; right column= mid-sagittal view. Pr=Proximal; D=Distal; L=Lateral; M=Medial; Po=Posterior; A=Anterior.

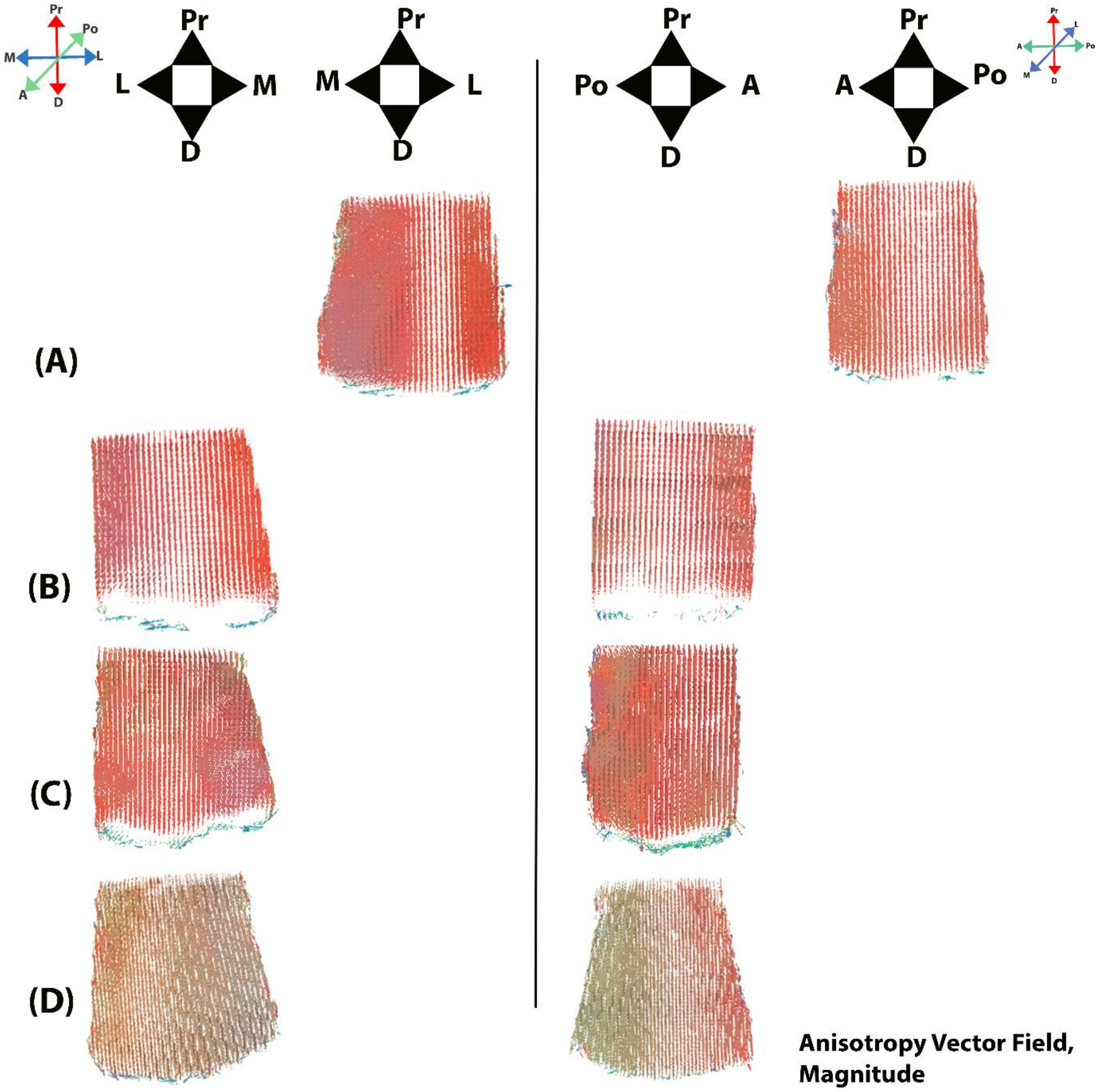

Figure 2: Fetal Anisotropy Vector Field, Direction Mapping. A) 28 weeks intrauterine, B) 28 weeks intrauterine; C) 32 weeks intrauterine; D) 38 weeks intrauterine. Coloured arrows indicate directionality of trabeculae. Red=superoinferior, green=mediolateral, blue=anteroposterior direction.

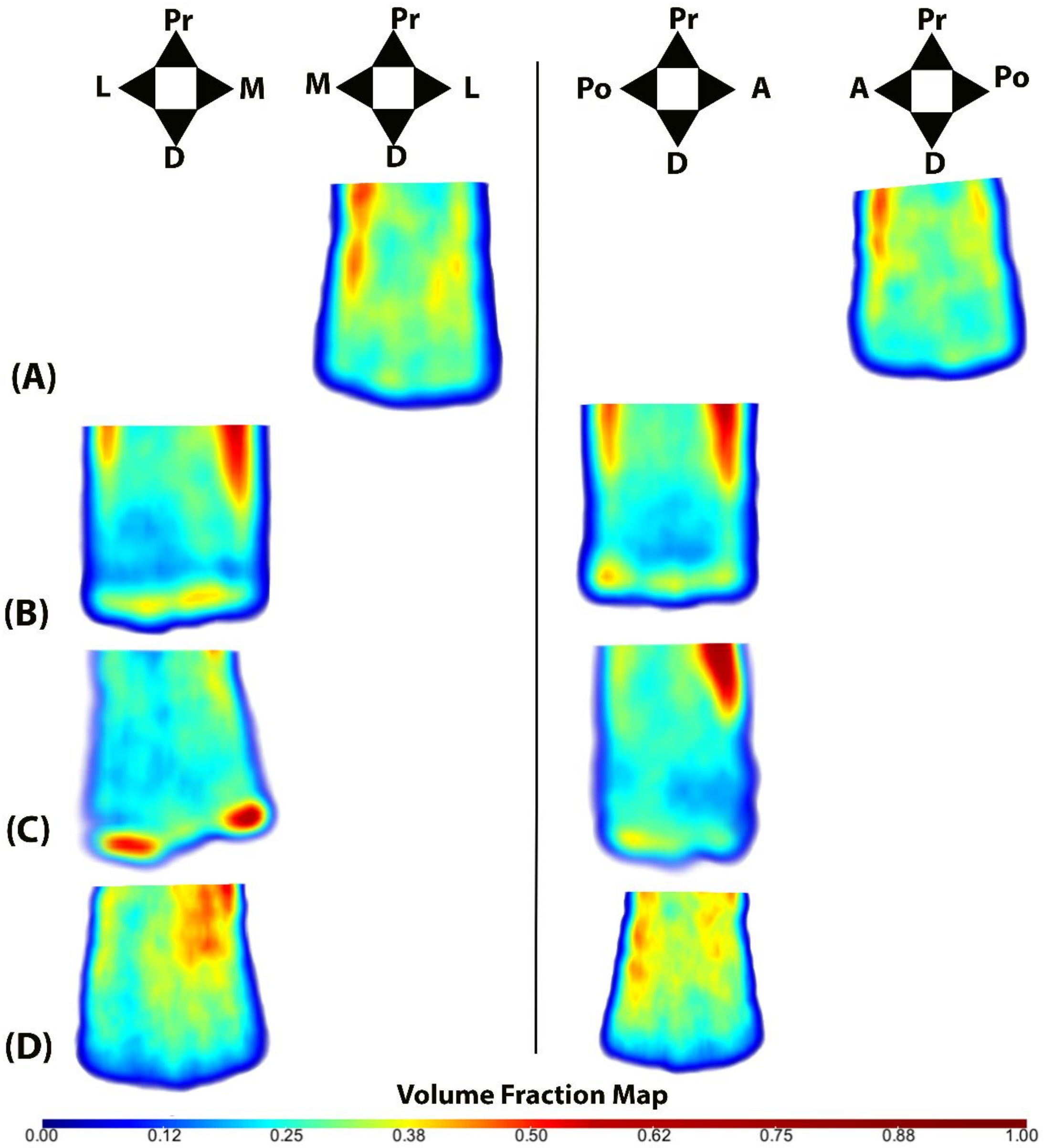

Figure 3: Fetal Bone Volume Fraction Mapping. A) 28 weeks intrauterine, B) 28 weeks intrauterine, C) 32 weeks intrauterine, D) 38 weeks intrauterine.

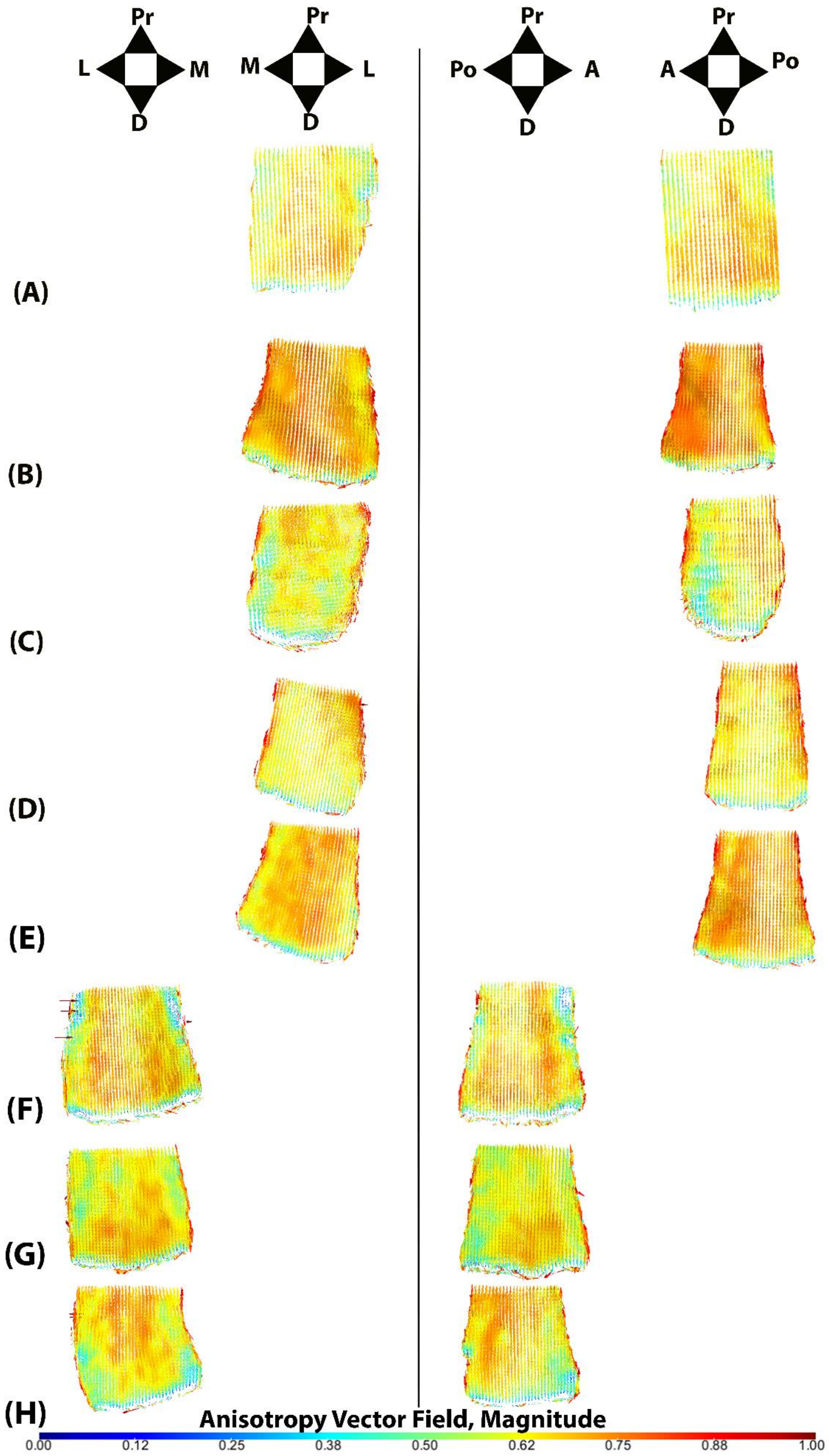

Figure 4: Perinatal Anisotropy Vector Field, Magnitude. A–H) Perinatal.

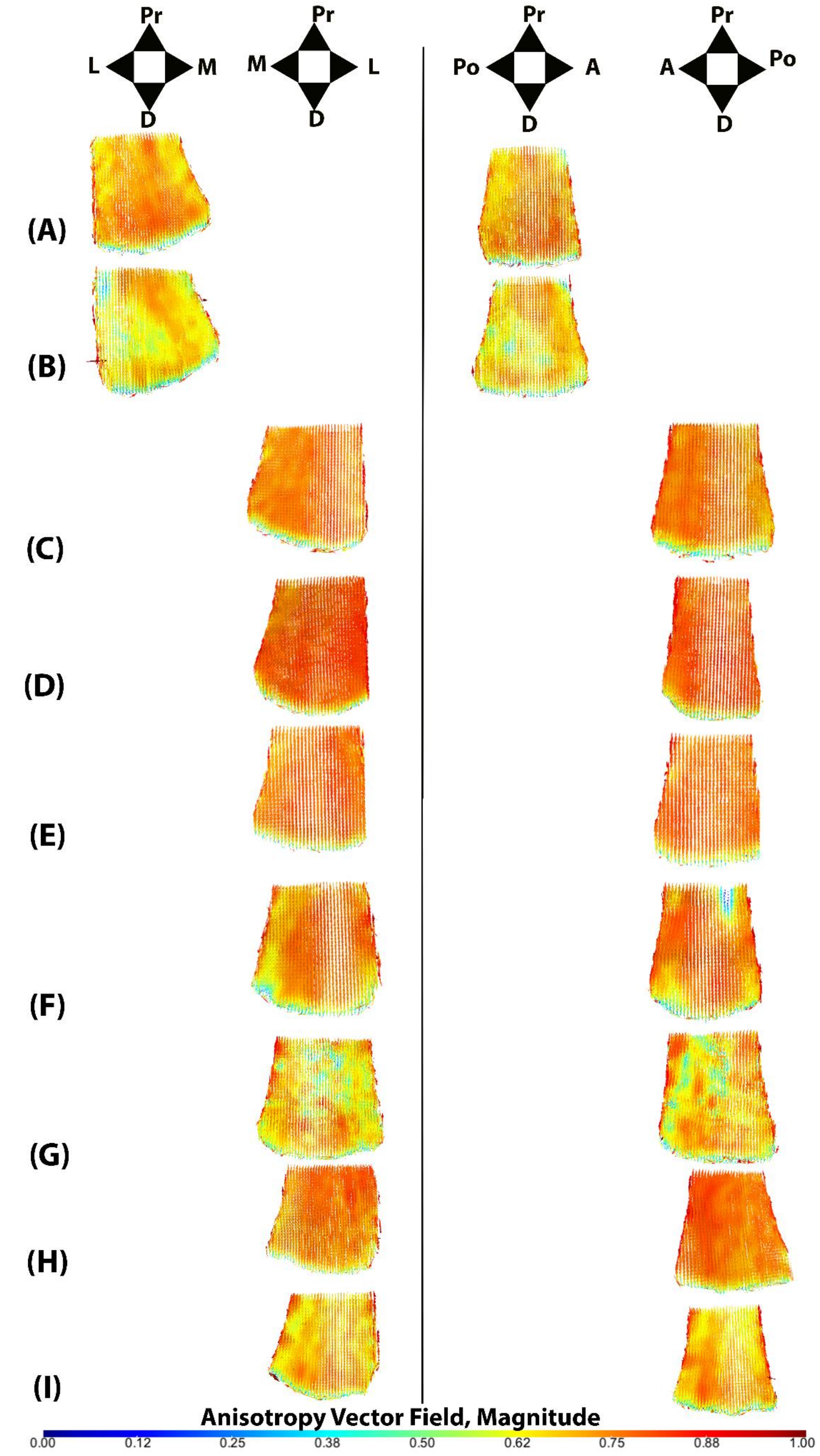

Figure 5: Perinatal Anisotropy Vector Field, Magnitude A–I) Perinatal.

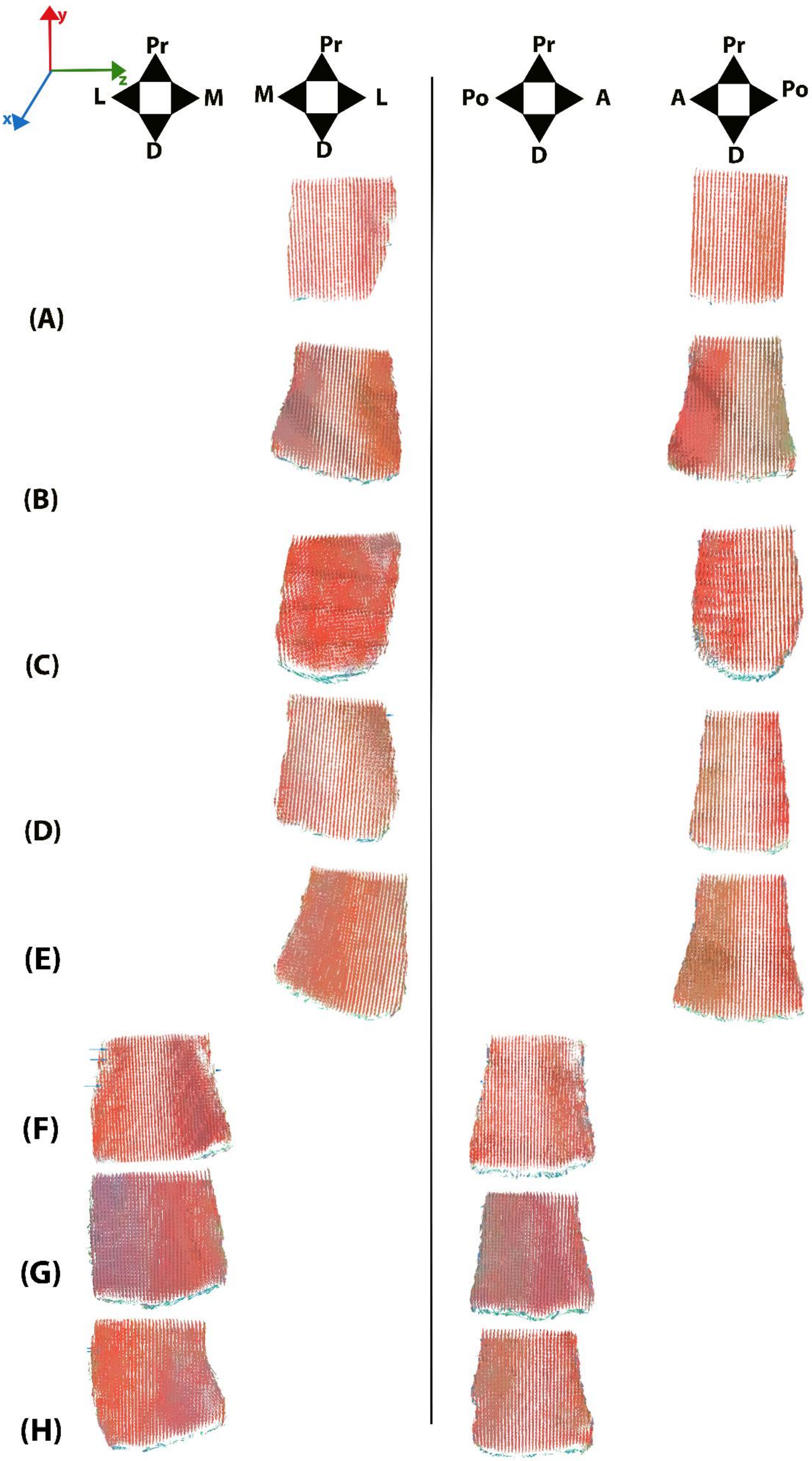

Figure 6: Perinatal Anisotropy Vector Field, Direction. A–H) Perinatal.

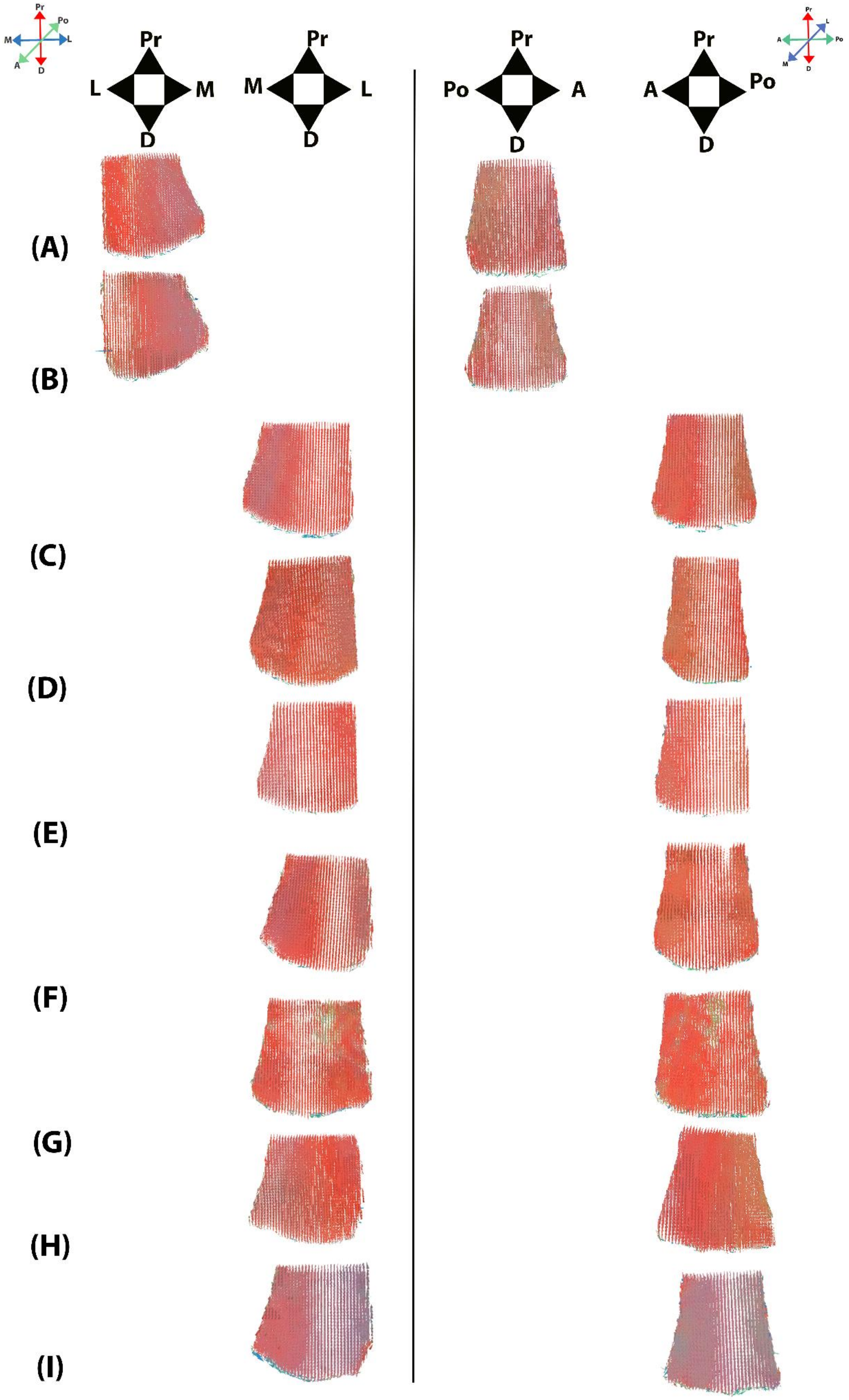

Figure 7: Perinatal Anisotropy Vector Field, Direction. A–I) Perinatal

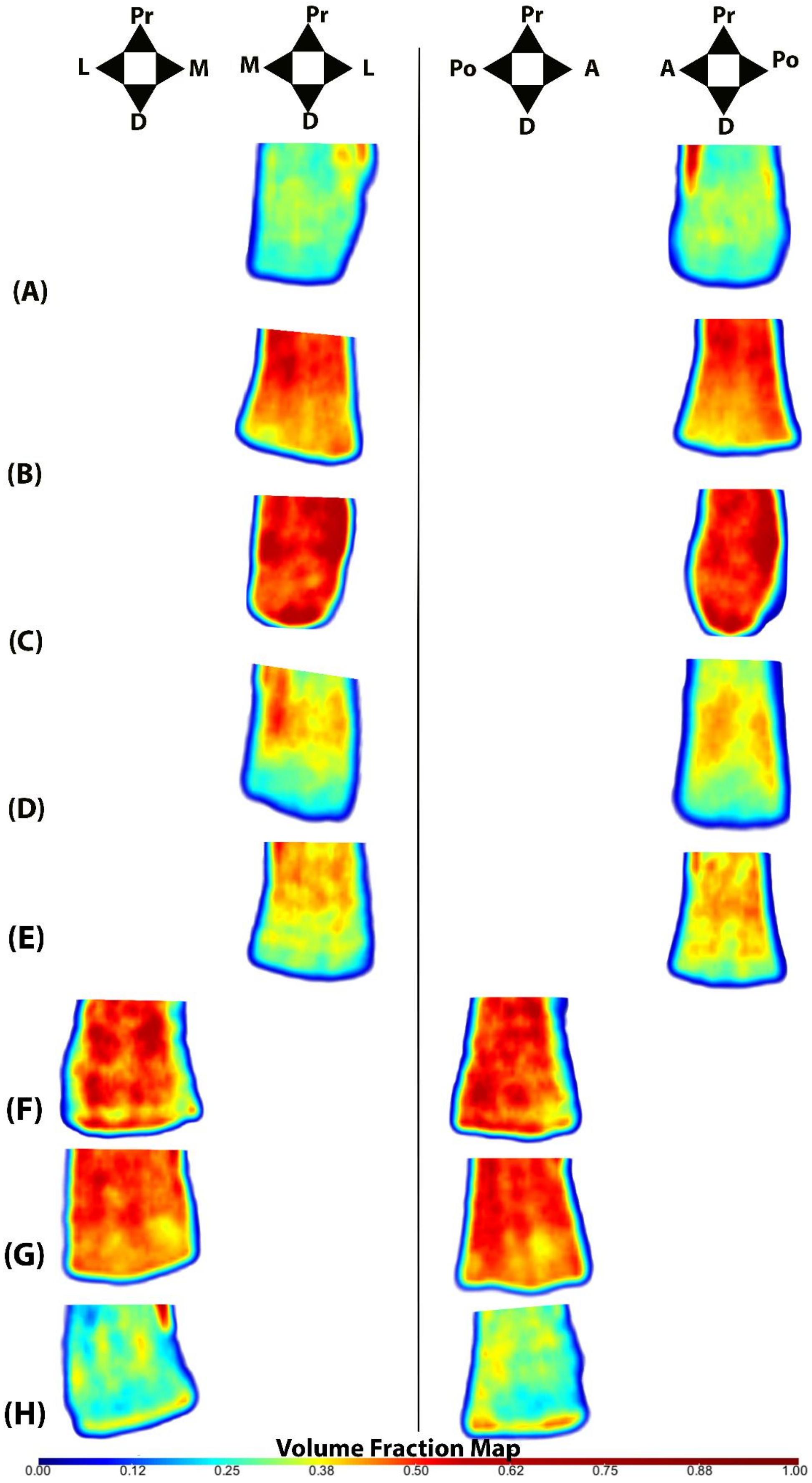

Figure 8: Perinatal Bone Volume Fraction Mapping. A–H) Perinatal

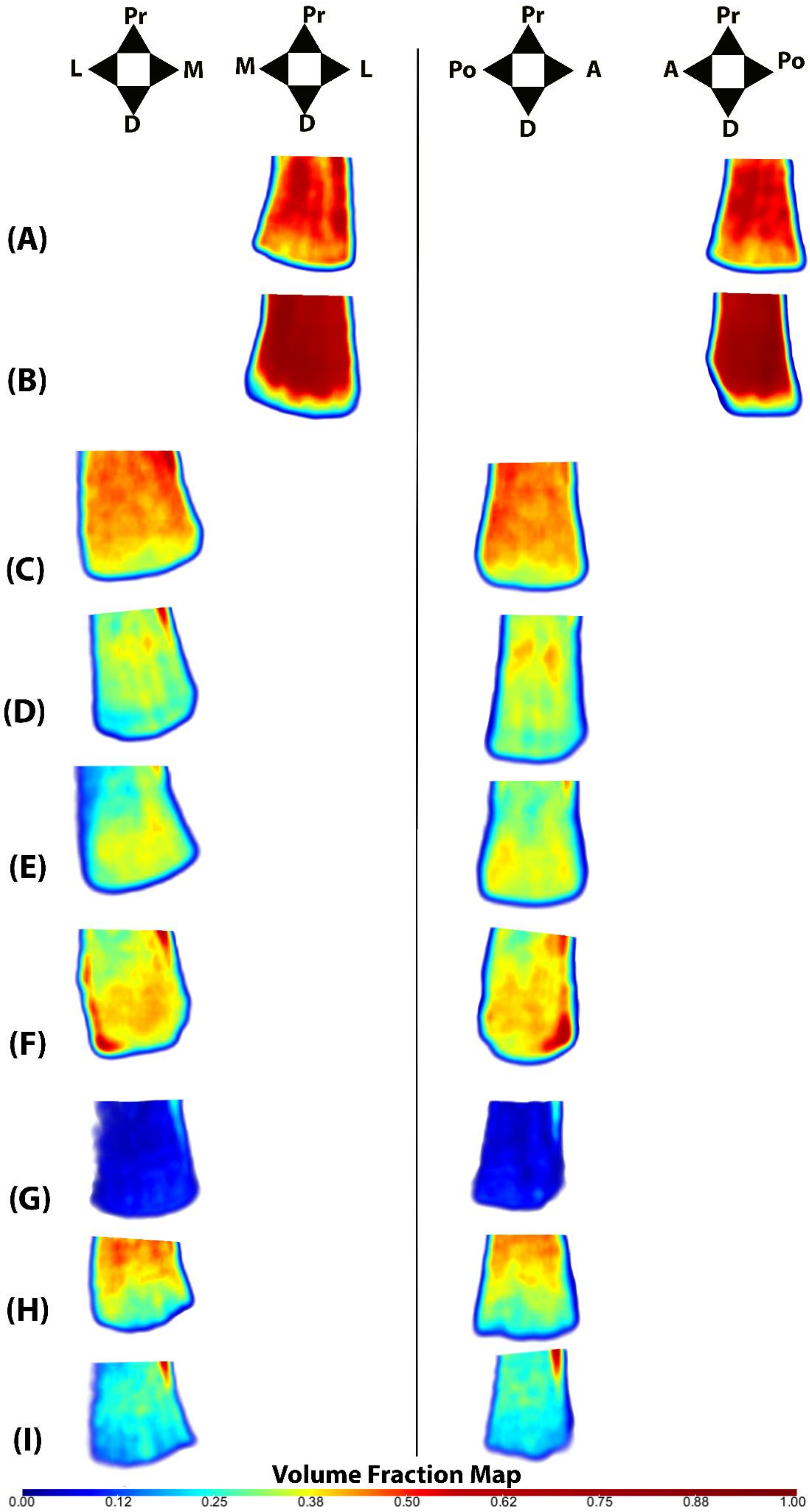

Figure 9: Perinatal Bone Volume Fraction Mapping. A–I) Perinatal

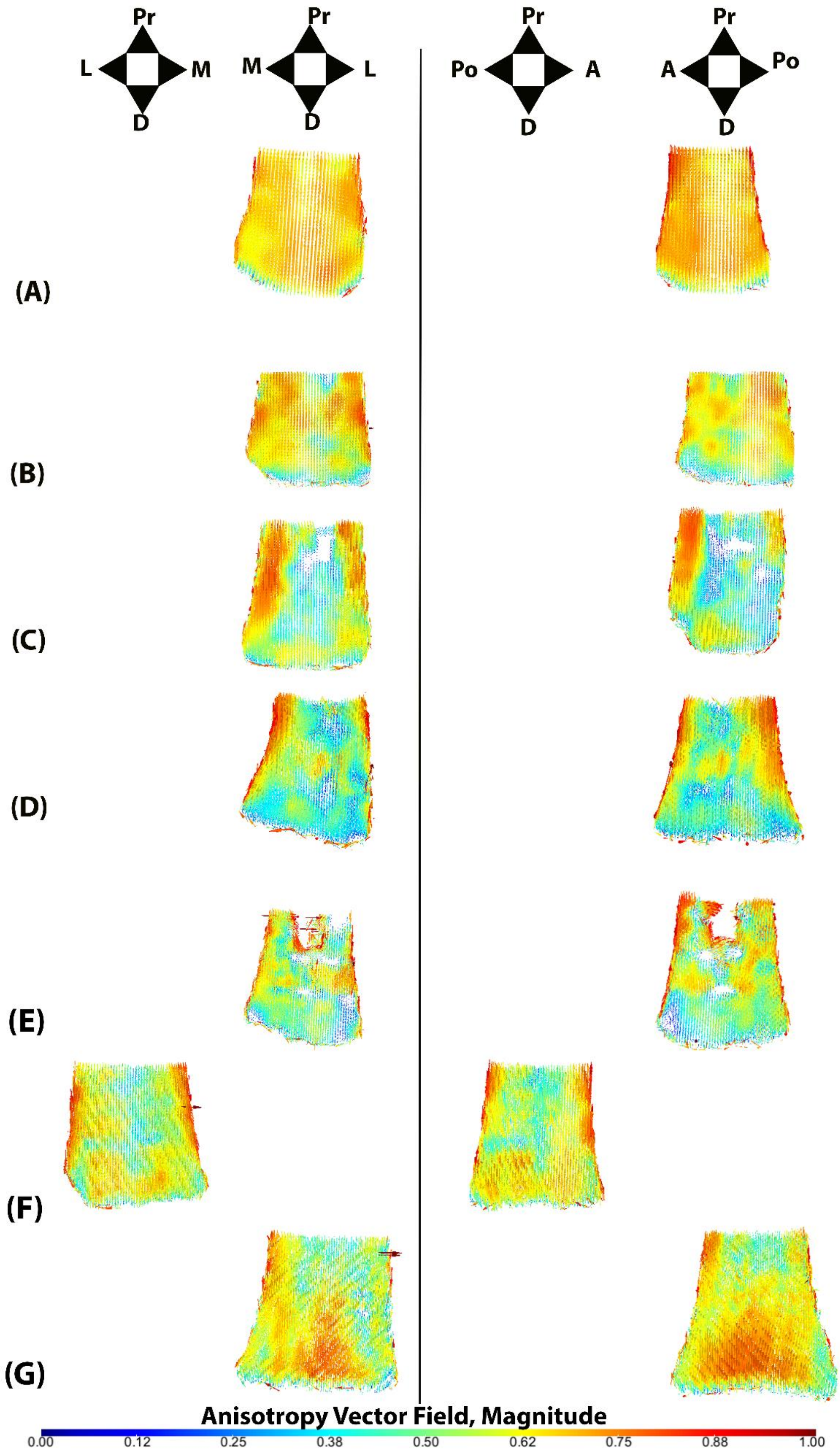

Figure 10: Infant and Early Toddler Anisotropy Vector Field, Magnitude. A) 0–6 months, B) 4–6 months, C) 5 months, D) 5 months, E) 1 year, F) 1–2 years, G) 2 years.

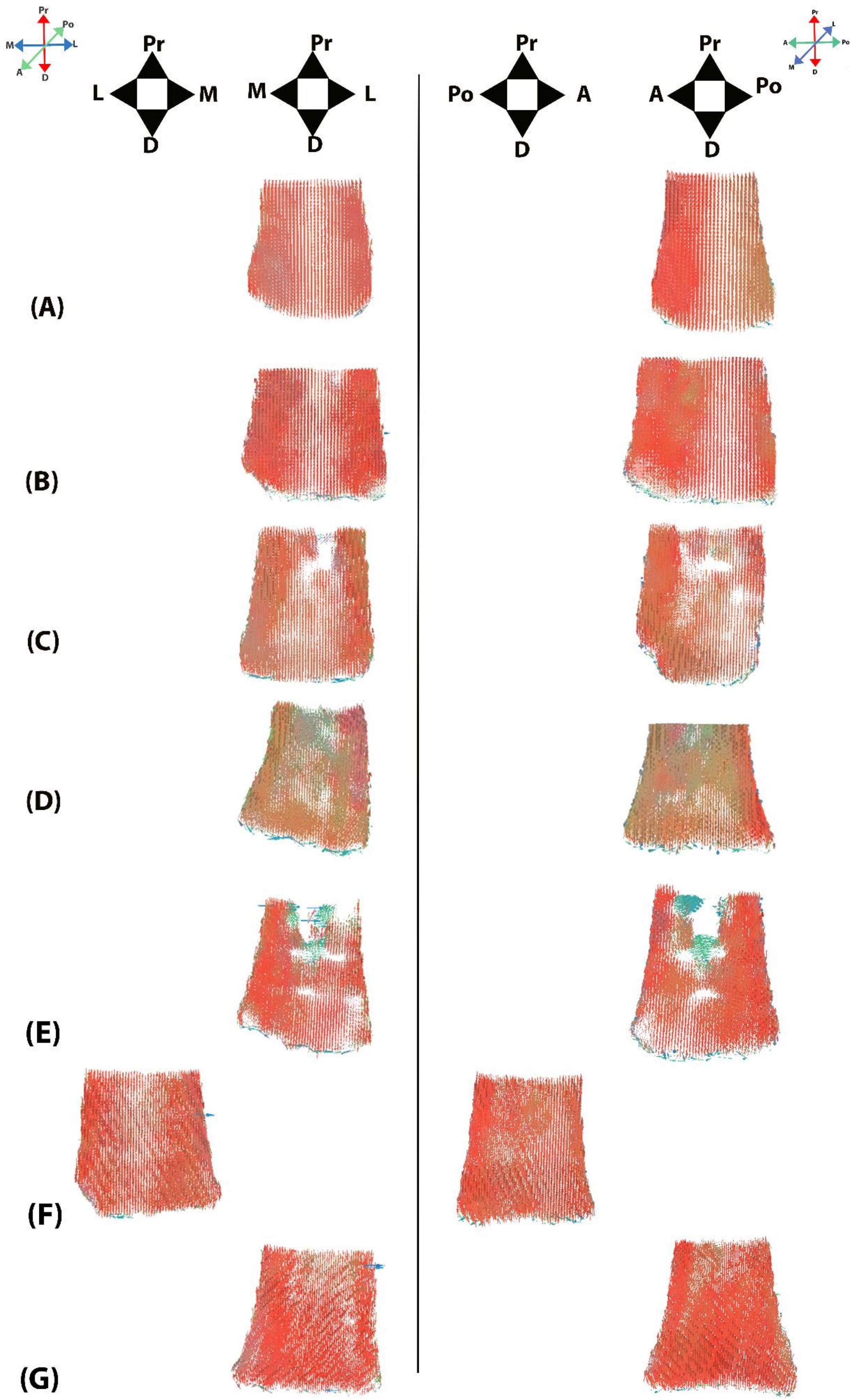

Figure 11: Infant and Early Toddler Anisotropy Vector Field, Direction. A) 0–6 months, B) 4–6 months, C) 5 months, D) 5 months, E) 1 year, F) 1–2 years, G) 2 years.

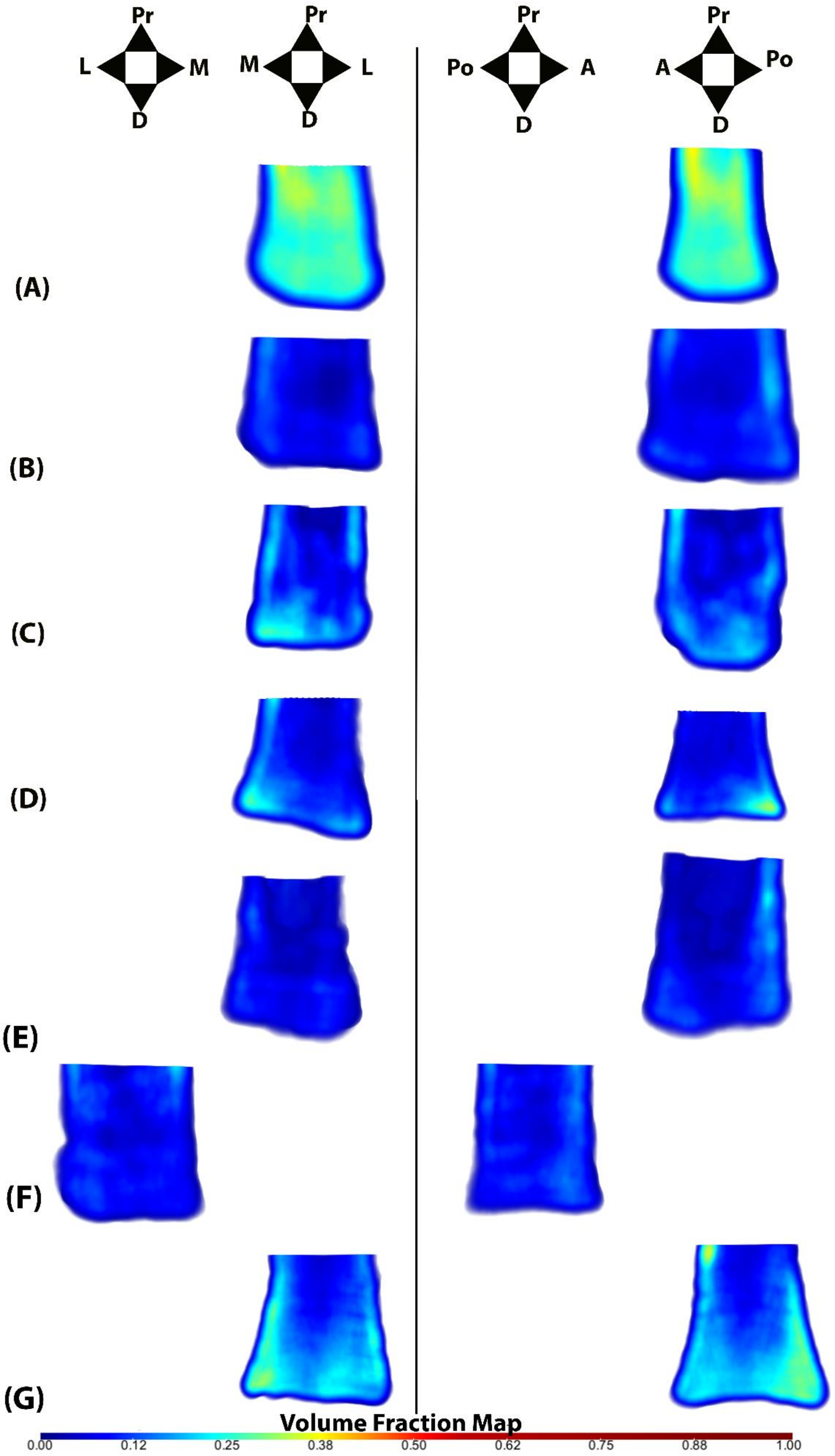

Figure 12: Infant and Early Toddler Bone Volume Fraction Map. A) 0–6 months, B) 4–6 months, C) 5 months, D) 5 months, E) 1 year, F) 1–2 years, G) 2 years.

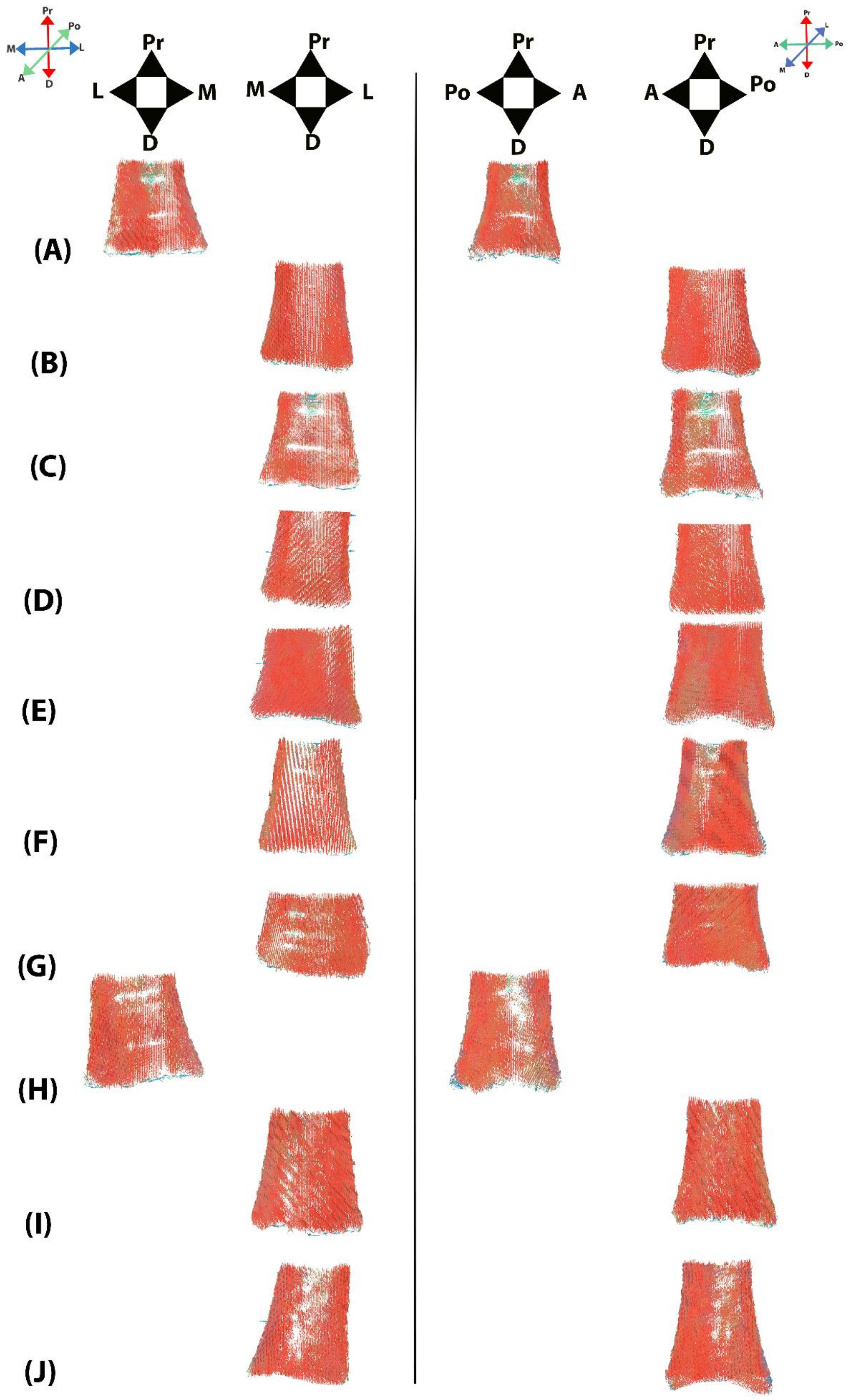

Figure 13: Early Childhood Anisotropy Vector Field, Direction. A) 2–5 years, B) 3 years 4 months, C) 3–6 years, D) 4 years, E) 4–5 years, F) 6 years, G) 6–10 years, H) 6–8 years, I) 7 years, J) 8 years.

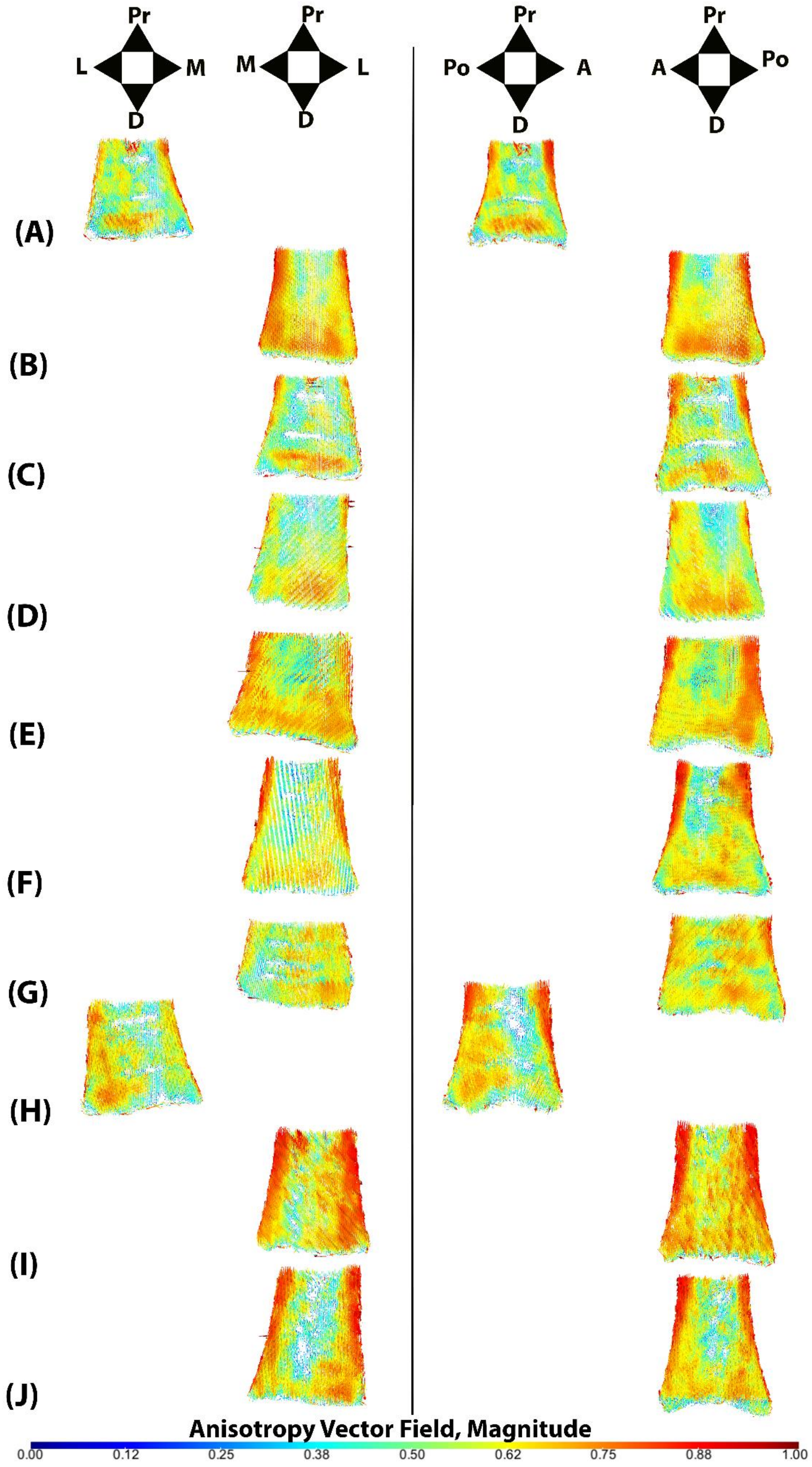

Figure 14: Early Childhood Anisotropy Vector Field, Magnitude. A) 2–5 years, B) 3 years 4 months, C) 3–6 years, D) 4 years, E) 4–5 years, F) 6 years, G) 6–10 years, H) 6–8 years, I) 7 years, J) 8 years.

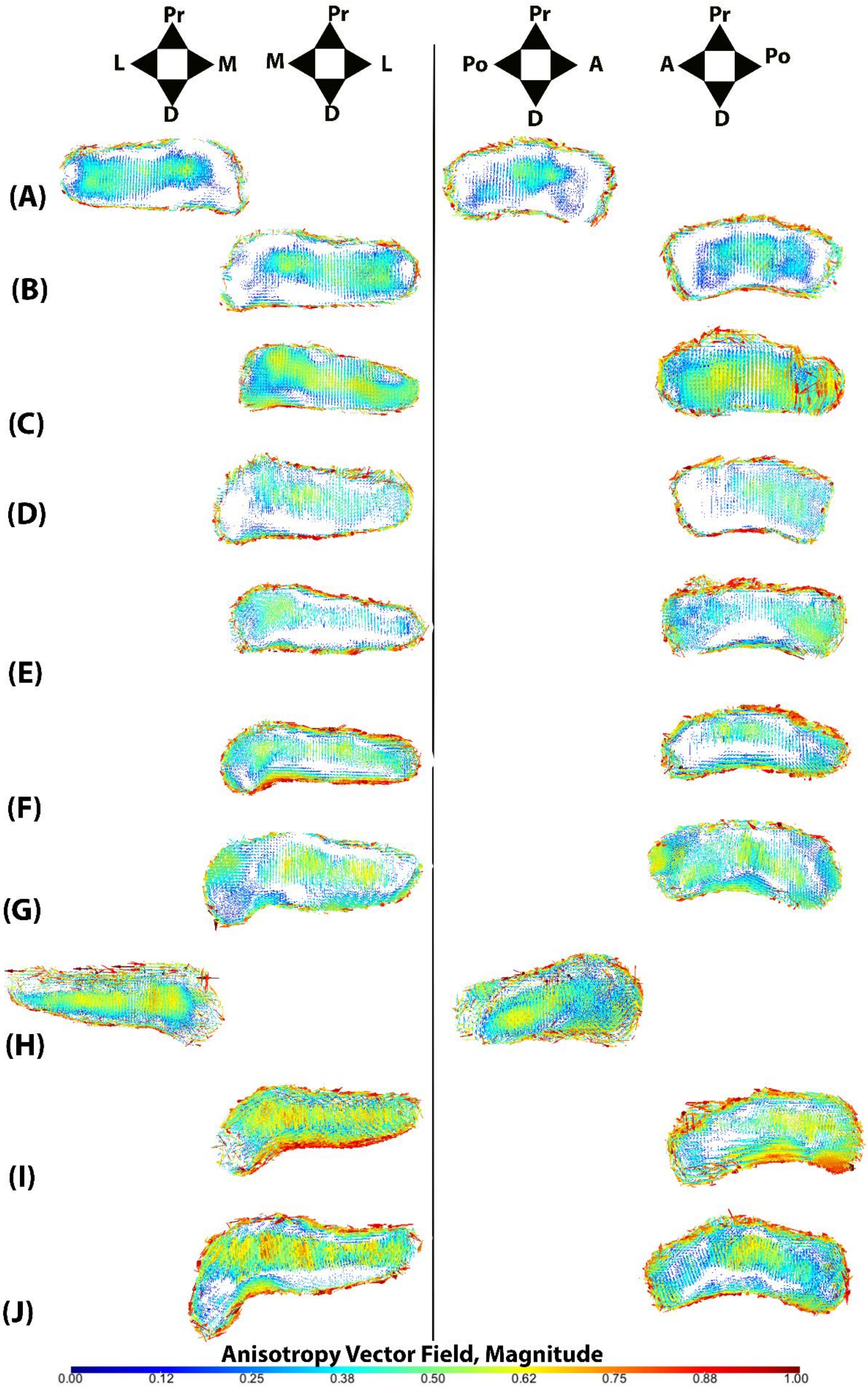

Figure 15: Early Childhood Anisotropy Vector Field, Magnitude. A) 2–5 years, B) 3 years C) 3–5 years, D) 3–6 years, E) 4 years, F) 4–5 years, G) 6 years, H) 6–8 years, I) 7 years, J) 8 years.

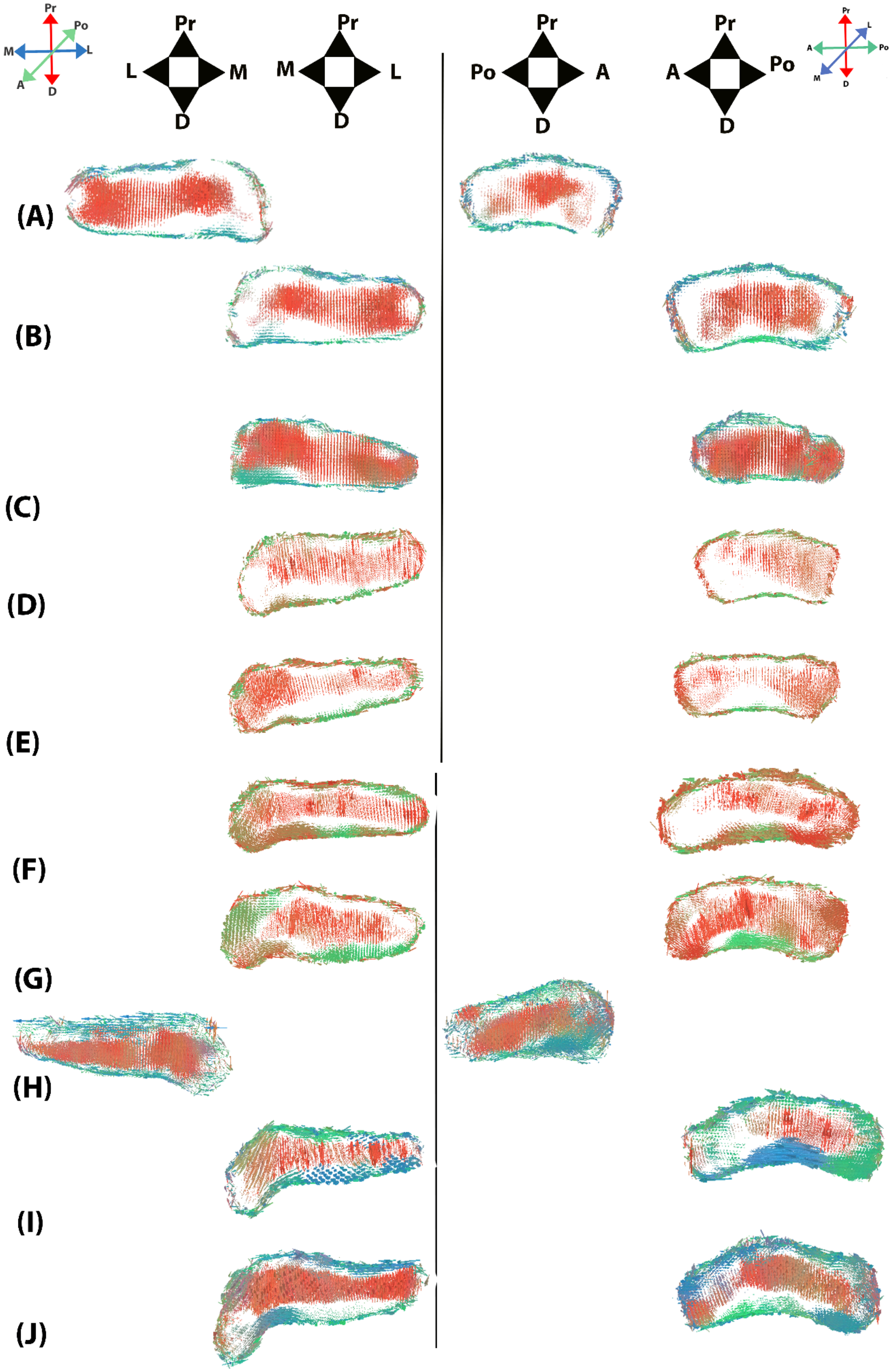

Figure 16: Early Childhood Anisotropy Vector Field, Direction. A) 2–5 years, B) 3 years C) 3–5 years, D) 3–6 years, E) 4 years, F) 4–5 years, G) 6 years, H) 6–8 years, I) 7 years, J) 8 years.

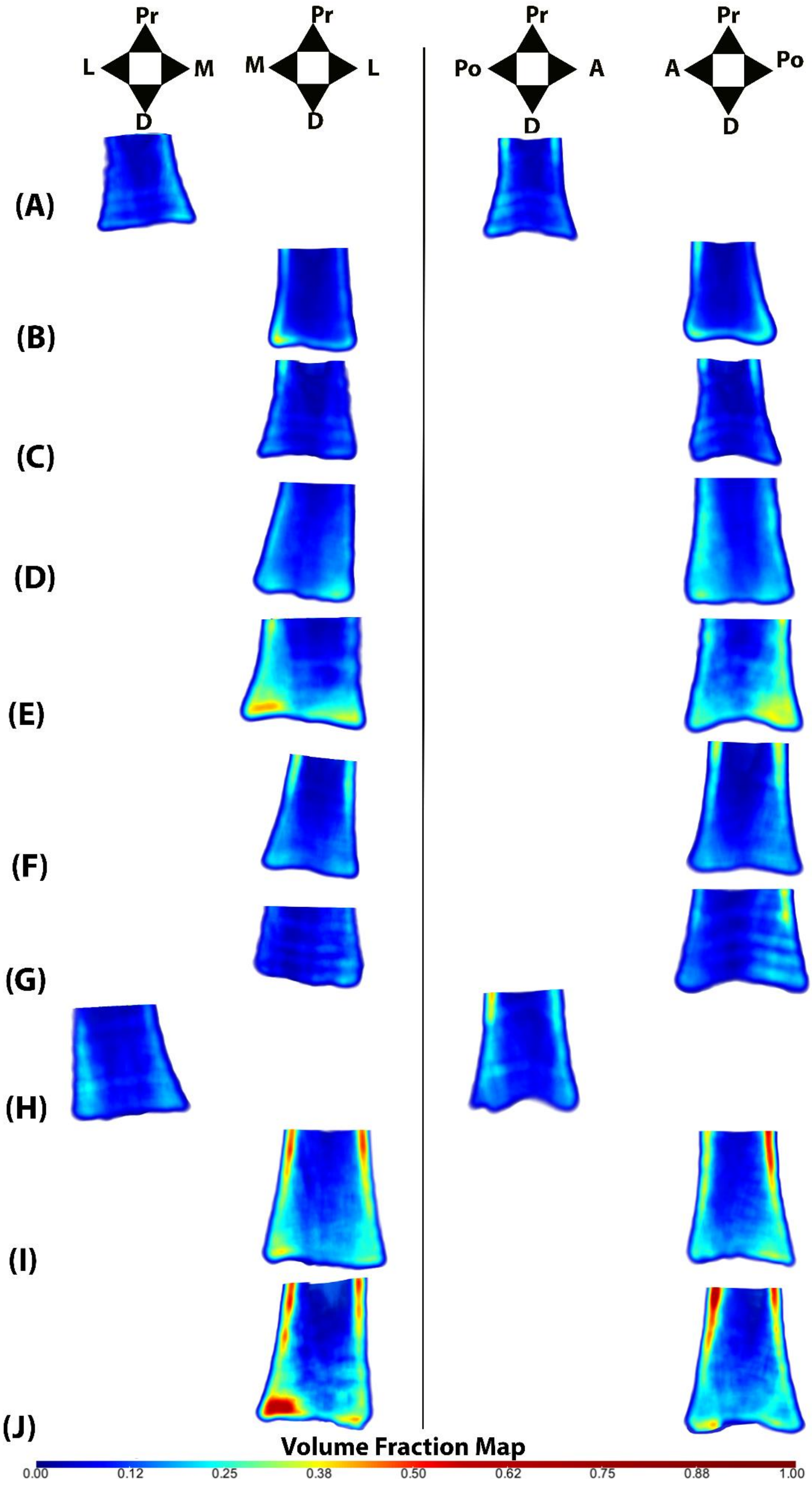

Figure 17: Early Childhood Distal Metaphyseal Bone Volume Fraction Maps. A) 2–5 years, B) 3 years 4 months, C) 3–6 years, D) 4 years, E) 4–5 years, F) 6 years, G) 6–10 years, H) 6–8 years, I) 7 years, J) 8 years.

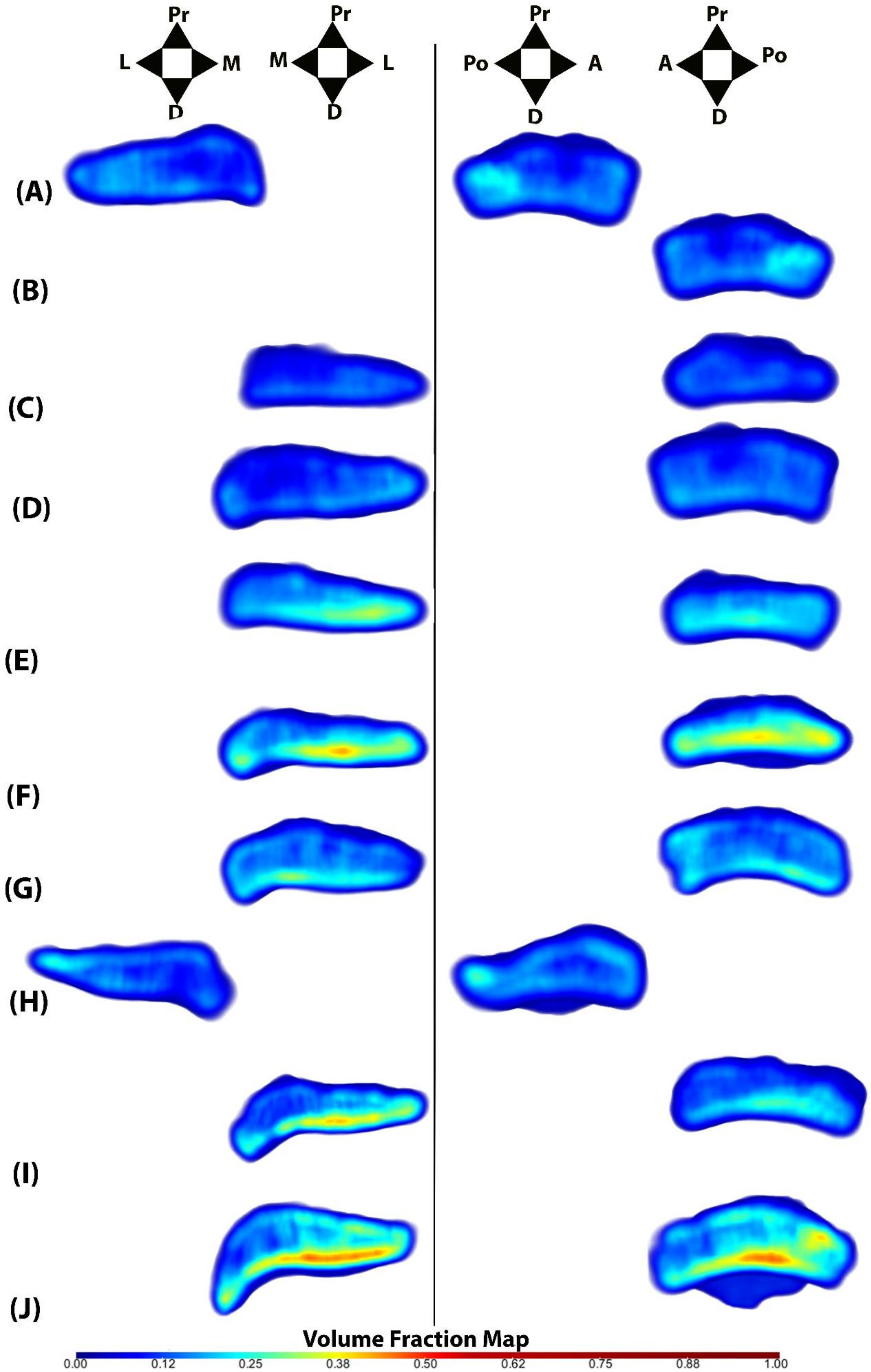

Figure 18: Early Childhood Distal Epiphysis Bone Volume Fraction Map. A) 2–5 years, B) 3 years C) 3–5 years, D) 3–6 years, E) 4 years, F) 4–5 years, G) 6 years, H) 6–8 years, I) 7 years, J) 8 years.

Volume of Interest Results

Table 1: Fetal volume of interest (VOI) descriptive statistics.

VOI	Statistic	DA	BV/TV	Tb.Sp (mm)	Tb.Th (mm)	Tb.N (mm^{-1})
A1 (N=4)	Mean	0.8625	0.27	0.1775	0.085	4.16
	Std. Deviation	0.03775	0.02449	0.04992	0.01915	1.2642
	Median	0.87	0.265	0.175	0.09	4.005
	Mean Rank	58.13	34.88	42.38	34.75	51.75
	Minimum	0.81	0.25	0.13	0.06	2.97
	Maximum	0.9	0.3	0.23	0.1	5.66
A2 (N=4)	Mean	0.77	0.3125	0.15	0.0825	4.645
	Std. Deviation	0.1472	0.04787	0.04761	0.015	1.34071
	Median	0.835	0.3	0.15	0.08	4.52
	Mean Rank	29.88	58.25	25.38	31.75	64.5
	Minimum	0.55	0.27	0.1	0.07	3.32
	Maximum	0.86	0.38	0.2	0.1	6.22
A3 (N=4)	Mean	0.85	0.265	0.17	0.08	4.31
	Std. Deviation	0.0469	0.05802	0.05888	0.01826	1.45407
	Median	0.83	0.27	0.17	0.08	4.06
	Mean Rank	43.13	36.5	39.63	28.75	53.25
	Minimum	0.82	0.19	0.11	0.06	3.03
	Maximum	0.92	0.33	0.23	0.1	6.09
A4 (N=4)	Mean	0.81	0.265	0.165	0.0775	4.4275
	Std. Deviation	0.04967	0.03317	0.03697	0.02062	1.05206
	Median	0.815	0.26	0.165	0.075	4.26
	Mean Rank	27	32.38	30.63	25.75	62
	Minimum	0.75	0.23	0.12	0.06	3.39
	Maximum	0.86	0.31	0.21	0.1	5.8
A5 (N=4)	Mean	0.825	0.255	0.1775	0.0825	4.1925
	Std. Deviation	0.08505	0.03786	0.045	0.01708	1.03339
	Median	0.855	0.24	0.17	0.085	4.245
	Mean Rank	44.88	25.25	41.13	31.5	56.75
	Minimum	0.7	0.23	0.14	0.06	3.15
	Maximum	0.89	0.31	0.23	0.1	5.13
A6 (N=4)	Mean	0.7775	0.2725	0.1525	0.0775	4.815
	Std. Deviation	0.06185	0.03862	0.05315	0.02062	1.41465
	Median	0.77	0.28	0.14	0.075	4.98
	Mean Rank	18.13	38.5	27.75	25.75	65.63
	Minimum	0.71	0.22	0.11	0.06	3.22
	Maximum	0.86	0.31	0.22	0.1	6.08
A7 (N=4)	Mean	0.81	0.315	0.1525	0.08	4.6775

	Std. Deviation	0.07958	0.07937	0.05123	0.01826	1.18258
	Median	0.825	0.305	0.145	0.08	4.795
	Mean Rank	34.38	53.13	26.75	28.75	66
	Minimum	0.7	0.24	0.1	0.06	3.25
	Maximum	0.89	0.41	0.22	0.1	5.87
A8 (N=4)	Mean	0.8325	0.2625	0.1625	0.0775	4.5125
	Std. Deviation	0.0556	0.02872	0.04787	0.015	1.2976
	Median	0.83	0.275	0.16	0.08	4.31
	Mean Rank	42.25	31.5	32.88	25.5	62.5
	Minimum	0.78	0.22	0.11	0.06	3.28
	Maximum	0.89	0.28	0.22	0.09	6.15
A9 (N=3)	Mean	0.83	0.2933	0.16	0.08	4.38
	Std. Deviation	0.04583	0.00577	0.04359	0.02646	1.09986
	Median	0.84	0.29	0.14	0.07	5.01
	Mean Rank	36.67	51.83	30	29	60
	Minimum	0.78	0.29	0.13	0.06	3.11
	Maximum	0.87	0.3	0.21	0.11	5.02
B1 (N=4)	Mean	0.855	0.275	0.205	0.1025	3.6725
	Std. Deviation	0.02646	0.05916	0.04796	0.02363	0.9365
	Median	0.86	0.28	0.195	0.11	3.69
	Mean Rank	50.75	39.63	53.38	55	41.38
	Minimum	0.82	0.21	0.16	0.07	2.66
	Maximum	0.88	0.33	0.27	0.12	4.65
B2 (N=4)	Mean	0.8575	0.305	0.19	0.1	3.745
	Std. Deviation	0.0263	0.05	0.02449	0.0216	0.82883
	Median	0.865	0.3	0.185	0.105	3.675
	Mean Rank	53.25	54.13	46.25	52.25	43.75
	Minimum	0.82	0.25	0.17	0.07	2.94
	Maximum	0.88	0.37	0.22	0.12	4.69
B3 (N=4)	Mean	0.86	0.28	0.19	0.0975	3.6875
	Std. Deviation	0.0469	0.03162	0.03559	0.02217	0.84622
	Median	0.855	0.275	0.185	0.1	3.57
	Mean Rank	51.88	41.75	46.88	49	40.13
	Minimum	0.81	0.25	0.16	0.07	2.93
	Maximum	0.92	0.32	0.23	0.12	4.68
B4 (N=4)	Mean	0.8625	0.29	0.1875	0.095	3.695
	Std. Deviation	0.01258	0.03266	0.02754	0.0238	0.60677
	Median	0.86	0.29	0.185	0.095	3.685
	Mean Rank	55.25	48.25	44.5	46.25	46.13
	Minimum	0.85	0.25	0.16	0.07	3.16

	Maximum	0.88	0.33	0.22	0.12	4.25
B5 (N=3)	Mean	0.855	0.25	0.21	0.0975	3.4525
	Std. Deviation	0.03317	0.00816	0.04082	0.02217	0.80942
	Median	0.85	0.25	0.21	0.1	3.37
	Mean Rank	48.63	20.75	59.5	49	33.75
	Minimum	0.82	0.24	0.17	0.07	2.67
	Maximum	0.9	0.26	0.25	0.12	4.4
B6 (N=4)	Mean	0.845	0.2725	0.1925	0.0975	3.6725
	Std. Deviation	0.03697	0.06238	0.02754	0.02217	0.64526
	Median	0.85	0.285	0.195	0.1	3.68
	Mean Rank	45.88	41.63	49.38	49	44.5
	Minimum	0.8	0.19	0.16	0.07	3.07
	Maximum	0.88	0.33	0.22	0.12	4.26
B7 (N=4)	Mean	0.86	0.2775	0.19	0.095	3.72
	Std. Deviation	0.02582	0.02986	0.03559	0.0238	0.86344
	Median	0.86	0.28	0.2	0.095	3.455
	Mean Rank	54.5	40.25	49.88	46.25	42.63
	Minimum	0.83	0.24	0.14	0.07	3.07
	Maximum	0.89	0.31	0.22	0.12	4.9
B8 (N=4)	Mean	0.8675	0.2525	0.205	0.095	3.5125
	Std. Deviation	0.01708	0.04992	0.03317	0.0238	0.72233
	Median	0.865	0.245	0.195	0.095	3.445
	Mean Rank	60.13	26.13	56.88	46.25	36.5
	Minimum	0.85	0.2	0.18	0.07	2.81
	Maximum	0.89	0.32	0.25	0.12	4.35
B9 (N=3)	Mean	0.85	0.31	0.1933	0.1033	3.4933
	Std. Deviation	0.02646	0.04583	0.00577	0.02517	0.38837
	Median	0.86	0.3	0.19	0.1	3.61
	Mean Rank	46.5	58.5	49.33	54.83	38.33
	Minimum	0.82	0.27	0.19	0.08	3.06
	Maximum	0.87	0.36	0.2	0.13	3.81
C1 (N=4)	Mean	0.8675	0.29	0.2125	0.1075	3.1675
	Std. Deviation	0.02872	0.05598	0.04031	0.02217	0.54082
	Median	0.875	0.3	0.2	0.11	3.255
	Mean Rank	61.38	48.63	59.63	59.88	26
	Minimum	0.83	0.22	0.18	0.08	2.5
	Maximum	0.89	0.34	0.27	0.13	3.66
C2 (N=4)	Mean	0.835	0.3475	0.1975	0.1225	3.2275
	Std. Deviation	0.03109	0.05909	0.02363	0.02754	0.48507
	Median	0.845	0.335	0.19	0.125	3.26

	Mean Rank	36.13	73	52.63	70.38	27
	Minimum	0.79	0.29	0.18	0.09	2.71
	Maximum	0.86	0.43	0.23	0.15	3.68
C3 (N=4)	Mean	0.8275	0.345	0.2075	0.12	3.25
	Std. Deviation	0.09287	0.05745	0.025	0.0216	0.53759
	Median	0.85	0.36	0.205	0.125	3.255
	Mean Rank	46.88	70.63	60.13	71.5	28.38
	Minimum	0.7	0.27	0.18	0.09	2.63
	Maximum	0.91	0.39	0.24	0.14	3.86
C4 (N=4)	Mean	0.83	0.3425	0.2025	0.1175	3.2475
	Std. Deviation	0.05354	0.04193	0.02872	0.02217	0.57558
	Median	0.825	0.335	0.2	0.12	3.165
	Mean Rank	39.38	74.25	55.25	68.75	29.25
	Minimum	0.78	0.3	0.17	0.09	2.64
	Maximum	0.89	0.4	0.24	0.14	4.02
C5 (N=4)	Mean	0.8625	0.2975	0.2175	0.1125	3.26
	Std. Deviation	0.035	0.04272	0.04113	0.02363	0.62005
	Median	59.63	51.63	63.5	64.88	28.25
	Mean Rank	0.88	0.295	0.21	0.12	3.15
	Minimum	0.81	0.25	0.18	0.08	2.66
	Maximum	0.88	0.35	0.27	0.13	4.08
Total	Mean	0.8392	0.2887	0.1858	0.0951	3.8647
	N	90	90	90	90	90
	Std. Deviation	0.05603	0.04941	0.04034	0.02352	0.97682
	Median	0.85	0.29	0.19	0.09	3.635
	Minimum	0.55	0.19	0.1	0.06	2.5
	Maximum	0.92	0.43	0.27	0.15	6.22

Table 2: Perinatal volume of interest (VOI) descriptive statistics.

VOI	Statistic	DA	BV/TV	Tb.Sp (mm)	Tb.Th (mm)	Tb.N (mm^{-1})
A1 (N=17)	Mean	0.88	0.35	0.19	0.11	3.45
	Std. Deviation	0.05	0.10	0.05	0.03	0.72
	Median	0.88	0.34	0.19	0.11	3.27
	Mean Rank	245.56	160.91	182.32	158.74	216.53
	Minimum	0.79	0.10	0.13	0.07	2.22
	Maximum	0.94	0.50	0.36	0.18	5.06
A2 (N=16)	Mean	0.84	0.36	0.18	0.11	3.63
	Std. Deviation	0.08	0.11	0.05	0.03	0.90
	Median	0.89	0.36	0.17	0.11	3.47
	Mean Rank	199.94	170.38	160.75	142.16	229.94
	Minimum	0.67	0.11	0.11	0.06	2.43
	Maximum	0.93	0.57	0.33	0.18	5.79
A3 (N=17)	Mean	0.85	0.36	0.18	0.11	3.67
	Std. Deviation	0.06	0.11	0.06	0.03	0.83
	Median	0.86	0.38	0.17	0.11	3.65
	Mean Rank	196.65	173.47	150.47	134.74	241.71
	Minimum	0.72	0.08	0.12	0.06	2.23

	Maximum	0.93	0.53	0.38	0.19	5.41
A4 (N=17)	Mean	0.82	0.35	0.18	0.11	3.74
	Std. Deviation	0.08	0.12	0.05	0.04	0.90
	Median	0.84	0.35	0.17	0.10	3.81
	Mean Rank	159.94	163.50	149.38	132.26	244.24
	Minimum	0.66	0.10	0.11	0.06	2.58
	Maximum	0.92	0.59	0.32	0.20	5.95
A5 (N=15)	Mean	0.86	0.33	0.19	0.10	3.63
	Std. Deviation	0.06	0.11	0.07	0.02	0.86
	Median	0.86	0.36	0.17	0.11	3.52
	Mean Rank	206.50	148.50	161.50	119.59	245.16
	Minimum	0.72	0.08	0.12	0.06	2.10
	Maximum	0.93	0.48	0.39	0.14	5.68
A6 (N=17)	Mean	0.81	0.36	0.18	0.11	3.65
	Std. Deviation	0.07	0.13	0.05	0.04	1.25
	Median	0.82	0.35	0.17	0.10	3.71
	Mean Rank	130.50	174.75	150.38	137.94	230.47
	Minimum	0.64	0.10	0.11	0.06	0.52
	Maximum	0.89	0.62	0.29	0.23	6.04
A7 (N=15)	Mean	0.84	0.33	0.18	0.10	3.81
	Std. Deviation	0.07	0.11	0.05	0.03	0.91
	Median	0.86	0.32	0.16	0.10	3.75
	Mean Rank	173.67	144.73	146.97	113.73	253.77
	Minimum	0.67	0.09	0.12	0.06	2.39
	Maximum	0.93	0.52	0.34	0.18	5.64
A8 (N=16)	Mean	0.83	0.37	0.17	0.11	3.82
	Std. Deviation	0.13	0.13	0.05	0.03	0.93
	Median	0.87	0.40	0.16	0.10	3.79
	Mean Rank	199.63	185.16	133.91	131.09	251.50
	Minimum	0.36	0.11	0.11	0.06	2.62
	Maximum	0.94	0.57	0.31	0.19	5.81
A9 (N=14)	Mean	0.82	0.35	0.18	0.11	3.67
	Std. Deviation	0.14	0.13	0.06	0.04	0.93
	Median	0.86	0.37	0.16	0.10	3.64
	Mean Rank	184.82	173.21	144.86	138.00	232.64
	Minimum	0.40	0.09	0.10	0.06	2.52
	Maximum	0.93	0.55	0.32	0.21	5.65
B1 (N=17)	Mean	0.85	0.39	0.20	0.14	3.13
	Std. Deviation	0.07	0.15	0.08	0.04	0.72
	Median	0.86	0.37	0.19	0.13	2.90
	Mean Rank	198.41	195.53	192.53	214.47	168.29
	Minimum	0.63	0.08	0.13	0.08	1.74
	Maximum	0.93	0.83	0.49	0.27	4.82
B2 (N=16)	Mean	0.84	0.40	0.20	0.13	3.13
	Std. Deviation	0.07	0.13	0.06	0.04	0.56
	Median	0.84	0.41	0.19	0.14	3.07
	Mean Rank	182.34	215.59	206.06	216.13	169.19
	Minimum	0.73	0.09	0.15	0.07	2.16
	Maximum	0.95	0.63	0.39	0.20	4.23
B3 (N=17)	Mean	0.87	0.37	0.21	0.13	3.19
	Std. Deviation	0.05	0.12	0.08	0.03	0.72
	Median	0.88	0.36	0.19	0.13	3.13
	Mean Rank	217.15	186.71	201.56	200.44	177.94
	Minimum	0.78	0.07	0.13	0.07	1.88
	Maximum	0.92	0.59	0.47	0.20	4.92
B4 (N=17)	Mean	0.84	0.39	0.20	0.13	3.18
	Std. Deviation	0.07	0.13	0.07	0.04	0.71
	Median	0.86	0.37	0.19	0.12	2.97
	Mean Rank	179.76	195.18	200.65	203.41	175.53
	Minimum	0.66	0.09	0.14	0.07	2.07
	Maximum	0.93	0.68	0.41	0.21	4.87
B5 (N=16)	Mean	0.84	0.39	0.22	0.14	3.07
	Std. Deviation	0.07	0.16	0.11	0.05	0.78
	Median	0.86	0.39	0.19	0.13	2.92
	Mean Rank	183.84	192.34	195.94	214.63	161.84
	Minimum	0.66	0.05	0.15	0.07	1.41
	Maximum	0.93	0.74	0.62	0.26	4.57

B6 (N=17)	Mean	0.82	0.39	0.20	0.14	3.08
	Std. Deviation	0.07	0.13	0.05	0.04	0.61
	Median	0.83	0.39	0.20	0.13	3.22
	Mean Rank	142.44	197.71	214.21	215.88	160.94
	Minimum	0.68	0.10	0.15	0.07	2.19
	Maximum	0.92	0.64	0.36	0.25	4.41
B7 (N=16)	Mean	0.86	0.37	0.20	0.12	3.21
	Std. Deviation	0.05	0.11	0.07	0.03	0.65
	Median	0.86	0.35	0.19	0.13	3.26
	Mean Rank	208.56	186.00	196.03	195.50	184.94
	Minimum	0.77	0.09	0.14	0.07	2.00
	Maximum	0.94	0.51	0.43	0.20	4.60
B8 (N=17)	Mean	0.86	0.37	0.20	0.13	3.20
	Std. Deviation	0.07	0.13	0.06	0.04	0.64
	Median	0.86	0.37	0.20	0.13	2.94
	Mean Rank	212.88	184.24	200.38	194.82	181.32
	Minimum	0.69	0.09	0.14	0.08	2.09
	Maximum	0.94	0.66	0.41	0.21	4.49
B9 (N=14)	Mean	0.85	0.37	0.21	0.13	3.13
	Std. Deviation	0.06	0.12	0.07	0.03	0.65
	Median	0.86	0.39	0.20	0.14	3.00
	Mean Rank	188.36	186.86	203.75	208.64	168.39
	Minimum	0.75	0.10	0.15	0.07	1.99
	Maximum	0.92	0.56	0.41	0.18	4.37
C1 (N=17)	Mean	0.85	0.41	0.22	0.15	2.88
	Std. Deviation	0.07	0.16	0.10	0.04	0.56
	Median	0.87	0.41	0.19	0.14	2.84
	Mean Rank	204.50	218.97	223.88	245.53	129.44
	Minimum	0.66	0.05	0.13	0.09	1.56
	Maximum	0.93	0.83	0.56	0.26	4.06
C2 (N=17)	Mean	0.83	0.44	0.21	0.15	2.91
	Std. Deviation	0.08	0.15	0.08	0.04	0.51
	Median	0.83	0.44	0.19	0.15	2.87
	Mean Rank	161.94	243.00	215.79	260.47	133.50
	Minimum	0.70	0.08	0.13	0.09	1.83
	Maximum	0.93	0.76	0.48	0.24	3.87
C3 (N=17)	Mean	0.87	0.40	0.22	0.14	2.90
	Std. Deviation	0.06	0.15	0.09	0.04	0.55
	Median	0.87	0.37	0.20	0.14	2.82
	Mean Rank	218.18	201.32	231.79	240.50	132.44
	Minimum	0.72	0.07	0.13	0.09	1.73
	Maximum	0.97	0.77	0.53	0.24	4.27
C4 (N=17)	Mean	0.83	0.41	0.21	0.15	2.96
	Std. Deviation	0.06	0.13	0.06	0.04	0.53
	Median	0.84	0.41	0.19	0.14	3.00
	Mean Rank	157.09	225.82	218.41	252.91	142.53
	Minimum	0.74	0.11	0.14	0.08	2.10
	Maximum	0.91	0.66	0.39	0.21	4.12
C5 (N=17)	Mean	0.83	0.40	0.22	0.15	2.88
	N	17.00	17.00	17.00	17.00	17.00
	Std. Deviation	0.10	0.17	0.09	0.06	0.68
	Median	0.85	0.41	0.21	0.14	2.70
	Mean Rank	179.94	200.74	237.32	240.21	124.21
	Minimum	0.54	0.07	0.14	0.08	1.69
	Maximum	0.94	0.79	0.52	0.31	4.78
Total	Mean	0.84	0.38	0.20	0.13	3.29
	Std. Deviation	0.08	0.13	0.07	0.04	0.81
	Median	0.86	0.38	0.18	0.12	3.18
	Minimum	0.36	0.05	0.10	0.06	0.52
	Maximum	0.97	0.83	0.62	0.31	6.04

Table 3: Infant and early toddler volume of interest (VOI) descriptive statistics.

VOI	Statistic	DA	BV/TV	Tb.Sp (mm)	Tb.Th (mm)	Tb.N (mm^{-1})
A1 (N=7)	Mean	0.87	0.14	0.38	0.11	2.20
	Std. Deviation	0.05	0.07	0.11	0.01	0.55

	Median	0.86	0.15	0.40	0.11	2.00
	Mean Rank	102.07	67.14	68.36	45.43	93.57
	Minimum	0.77	0.07	0.21	0.09	1.63
	Maximum	0.93	0.26	0.52	0.12	3.24
A2 (N=7)	Mean	0.81	0.20	0.33	0.11	2.45
	Std. Deviation	0.09	0.08	0.11	0.02	0.69
	Median	0.85	0.19	0.34	0.12	2.30
	Mean Rank	73.71	101.50	48.00	74.93	106.79
	Minimum	0.64	0.10	0.19	0.09	1.66
	Maximum	0.89	0.30	0.49	0.13	3.66
A3 (N=7)	Mean	0.82	0.17	0.35	0.11	2.29
	Std. Deviation	0.08	0.06	0.08	0.01	0.56
	Median	0.81	0.15	0.34	0.12	2.24
	Mean Rank	77.21	88.14	54.36	59.79	100.79
	Minimum	0.70	0.10	0.20	0.09	1.81
	Maximum	0.91	0.25	0.43	0.12	3.48
A4 (N=7)	Mean	0.78	0.19	0.31	0.11	2.55
	Std. Deviation	0.11	0.07	0.08	0.02	0.56
	Median	0.82	0.22	0.30	0.10	2.47
	Mean Rank	58.79	98.14	42.64	49.07	118.21
	Minimum	0.59	0.11	0.22	0.09	1.91
	Maximum	0.89	0.30	0.42	0.13	3.38
A5 (N=6)	Mean	0.83	0.17	0.37	0.11	2.25
	Std. Deviation	0.06	0.07	0.12	0.01	0.69
	Median	0.84	0.15	0.37	0.11	2.12
	Mean Rank	75.25	85.50	66.25	51.25	91.50
	Minimum	0.74	0.10	0.20	0.10	1.50
	Maximum	0.92	0.28	0.53	0.12	3.44
A6 (N=7)	Mean	0.77	0.19	0.32	0.11	2.55
	Std. Deviation	0.14	0.08	0.10	0.02	0.60
	Median	0.83	0.22	0.29	0.11	2.46
	Mean Rank	59.43	97.43	45.36	52.21	115.14
	Minimum	0.48	0.10	0.22	0.08	1.77
	Maximum	0.89	0.31	0.46	0.13	3.31
A7 (N=7)	Mean	0.80	0.18	0.32	0.10	2.54
	Std. Deviation	0.06	0.06	0.09	0.01	0.59
	Median	0.77	0.18	0.28	0.10	2.63
	Mean Rank	53.86	94.36	47.00	38.14	116.43
	Minimum	0.73	0.09	0.19	0.08	1.84
	Maximum	0.89	0.25	0.45	0.12	3.62
A8 (N=6)	Mean	0.81	0.18	0.34	0.11	2.41
	Std. Deviation	0.08	0.05	0.09	0.01	0.71
	Median	0.80	0.18	0.33	0.11	2.29
	Mean Rank	60.42	100.58	50.42	46.00	105.75
	Minimum	0.72	0.13	0.18	0.08	1.83
	Maximum	0.92	0.28	0.44	0.11	3.79
A9 (N=7)	Mean	0.85	0.18	0.33	0.11	2.43
	Std. Deviation	0.04	0.07	0.09	0.01	0.66
	Median	0.87	0.18	0.34	0.11	2.24
	Mean Rank	90.71	96.86	49.57	54.07	107.86
	Minimum	0.79	0.12	0.18	0.08	1.87
	Maximum	0.90	0.28	0.44	0.12	3.77
B1 (N=7)	Mean	0.77	0.12	0.50	0.12	1.80
	Std. Deviation	0.18	0.07	0.19	0.01	0.58
	Median	0.84	0.11	0.48	0.12	1.71
	Mean Rank	74.50	53.79	99.07	91.57	58.71
	Minimum	0.45	0.05	0.24	0.10	1.06
	Maximum	0.91	0.25	0.87	0.14	2.93
B2 (N=7)	Mean	0.83	0.18	0.41	0.13	1.94
	Std. Deviation	0.07	0.08	0.11	0.02	0.52
	Median	0.85	0.15	0.43	0.13	1.90
	Mean Rank	81.07	94.14	80.36	103.71	71.36
	Minimum	0.73	0.12	0.21	0.10	1.36
	Maximum	0.91	0.32	0.57	0.16	3.02
B3 (N=7)	Mean	0.85	0.15	0.43	0.12	1.95
	Std. Deviation	0.06	0.07	0.12	0.01	0.57
	Median	0.87	0.12	0.46	0.13	1.83

	Mean Rank	94.00	70.50	87.07	92.50	70.50
	Minimum	0.73	0.08	0.22	0.10	1.35
	Maximum	0.91	0.27	0.61	0.14	3.14
B4 (N=7)	Mean	0.83	0.16	0.42	0.12	1.99
	Std. Deviation	0.07	0.06	0.13	0.01	0.58
	Median	0.86	0.15	0.38	0.12	2.03
	Mean Rank	81.79	80.93	79.50	99.07	75.57
	Minimum	0.69	0.10	0.23	0.11	1.27
	Maximum	0.90	0.26	0.64	0.15	3.07
B5 (N=6)	Mean	0.81	0.15	0.46	0.12	1.86
	Std. Deviation	0.07	0.08	0.13	0.01	0.56
	Median	0.81	0.11	0.50	0.13	1.67
	Mean Rank	58.83	63.25	98.75	98.50	58.42
	Minimum	0.70	0.08	0.23	0.11	1.43
	Maximum	0.90	0.29	0.59	0.14	2.93
B6 (N=7)	Mean	0.84	0.16	0.42	0.12	1.94
	Std. Deviation	0.07	0.06	0.12	0.02	0.49
	Median	0.86	0.18	0.38	0.12	1.91
	Mean Rank	82.79	80.00	81.50	93.50	74.71
	Minimum	0.72	0.10	0.26	0.10	1.31
	Maximum	0.89	0.24	0.62	0.16	2.86
B7 (N=7)	Mean	0.86	0.16	0.41	0.12	2.01
	Std. Deviation	0.01	0.06	0.12	0.02	0.55
	Median	0.87	0.15	0.39	0.12	1.97
	Mean Rank	95.21	80.57	75.79	89.43	80.64
	Minimum	0.84	0.09	0.24	0.10	1.28
	Maximum	0.88	0.25	0.62	0.14	3.08
B8 (N=7)	Mean	0.84	0.16	0.44	0.12	1.92
	Std. Deviation	0.05	0.06	0.12	0.01	0.61
	Median	0.85	0.14	0.47	0.12	1.70
	Mean Rank	78.75	83.08	90.00	94.92	63.75
	Minimum	0.78	0.11	0.22	0.10	1.45
	Maximum	0.90	0.28	0.57	0.14	3.12
B9 (N=7)	Mean	0.86	0.16	0.44	0.12	1.92
	Std. Deviation	0.03	0.07	0.13	0.02	0.62
	Median	0.87	0.13	0.42	0.12	1.81
	Mean Rank	97.14	82.64	86.79	96.00	67.71
	Minimum	0.83	0.09	0.21	0.11	1.34
	Maximum	0.91	0.31	0.60	0.15	3.18
C1 (N=7)	Mean	0.83	0.09	0.71	0.11	1.49
	Std. Deviation	0.06	0.07	0.42	0.02	0.63
	Median	0.85	0.06	0.55	0.11	1.59
	Mean Rank	76.29	28.36	124.07	60.86	37.21
	Minimum	0.72	0.01	0.27	0.08	0.58
	Maximum	0.89	0.24	1.60	0.13	2.63
C2 (N=7)	Mean	0.86	0.17	0.47	0.14	1.72
	Std. Deviation	0.04	0.07	0.12	0.03	0.45
	Median	0.87	0.15	0.49	0.13	1.67
	Mean Rank	96.00	85.71	101.50	125.93	49.64
	Minimum	0.80	0.11	0.24	0.12	1.28
	Maximum	0.90	0.30	0.59	0.20	2.62
C3 (N=7)	Mean	0.84	0.12	0.55	0.12	1.61
	Std. Deviation	0.04	0.08	0.19	0.02	0.56
	Median	0.87	0.12	0.50	0.12	1.53
	Mean Rank	76.71	53.71	114.43	92.93	41.64
	Minimum	0.77	0.06	0.25	0.10	0.91
	Maximum	0.87	0.27	0.88	0.15	2.72
C4 (N=7)	Mean	0.86	0.14	0.47	0.12	1.78
	Std. Deviation	0.05	0.07	0.13	0.02	0.47
	Median	0.87	0.12	0.47	0.12	1.73
	Mean Rank	99.36	67.57	99.43	90.50	57.43
	Minimum	0.78	0.09	0.27	0.10	1.14
	Maximum	0.92	0.27	0.70	0.15	2.67
C5 (N=6)	Mean	0.79	0.13	0.57	0.13	1.58
	Std. Deviation	0.10	0.07	0.18	0.02	0.56
	Median	0.83	0.10	0.60	0.13	1.44
	Mean Rank	52.00	50.17	120.00	105.75	37.00

	Minimum	0.60	0.07	0.27	0.10	1.06
	Maximum	0.85	0.26	0.79	0.14	2.66
Total	Mean	0.83	0.16	0.42	0.12	2.05
	Std. Deviation	0.08	0.07	0.17	0.02	0.63
	Median	0.85	0.14	0.42	0.12	1.91
	Minimum	0.45	0.01	0.18	0.08	0.58
	Maximum	0.93	0.32	1.60	0.20	3.79

Table 4: Early childhood volume of interest (VOI) descriptive statistics.

VOI	Statistic	DA	BV/TV	Tb.Sp (mm)	Tb.Th (mm)	Tb.N (mm^{-1})
A1 (N=10)	Mean	0.89	0.14	0.53	0.13	1.56
	Std. Deviation	0.05	0.06	0.11	0.02	0.28
	Median	0.91	0.13	0.54	0.13	1.46
	Mean Rank	254.95	94.60	144.30	72.45	196.45
	Minimum	0.78	0.06	0.37	0.10	1.21
	Maximum	0.93	0.24	0.71	0.18	2.01
A2 (N=10)	Mean	0.87	0.21	0.46	0.15	1.70
	Std. Deviation	0.04	0.07	0.10	0.02	0.33
	Median	0.89	0.20	0.48	0.15	1.59
	Mean Rank	225.00	198.75	92.00	136.45	234.95
	Minimum	0.81	0.12	0.29	0.11	1.39
	Maximum	0.94	0.34	0.57	0.19	2.35
A3 (N=10)	Mean	0.86	0.19	0.47	0.15	1.67
	Std. Deviation	0.05	0.05	0.08	0.02	0.25
	Median	0.87	0.18	0.48	0.15	1.59
	Mean Rank	210.50	184.05	92.00	129.60	237.10
	Minimum	0.79	0.11	0.34	0.11	1.38
	Maximum	0.93	0.25	0.58	0.17	2.08
A4 (N=10)	Mean	0.80	0.26	0.40	0.15	1.85
	Std. Deviation	0.11	0.13	0.11	0.03	0.31
	Median	0.84	0.23	0.40	0.15	1.84
	Mean Rank	156.80	231.15	56.15	137.25	273.50
	Minimum	0.55	0.09	0.26	0.12	1.32
	Maximum	0.93	0.51	0.63	0.24	2.41
A5 (N=10)	Mean	0.91	0.20	0.43	0.15	1.78
	Std. Deviation	0.03	0.06	0.07	0.02	0.26
	Median	0.93	0.19	0.45	0.14	1.68
	Mean Rank	281.30	191.70	59.00	127.05	268.50
	Minimum	0.87	0.13	0.30	0.11	1.53
	Maximum	0.95	0.30	0.50	0.19	2.30
A6 (N=10)	Mean	0.78	0.26	0.40	0.16	1.86
	Std. Deviation	0.18	0.12	0.10	0.03	0.34
	Median	0.86	0.20	0.39	0.15	1.75
	Mean Rank	164.75	227.80	56.40	163.60	271.20
	Minimum	0.31	0.14	0.22	0.12	1.45
	Maximum	0.91	0.50	0.55	0.22	2.47
A7 (N=10)	Mean	0.81	0.24	0.40	0.15	1.85
	Std. Deviation	0.12	0.08	0.08	0.02	0.29
	Median	0.86	0.25	0.40	0.15	1.81
	Mean Rank	166.60	236.55	51.65	137.45	279.15
	Minimum	0.55	0.12	0.27	0.12	1.48
	Maximum	0.89	0.36	0.53	0.20	2.47
A8 (N=10)	Mean	0.89	0.21	0.43	0.15	1.78
	Std. Deviation	0.06	0.05	0.06	0.02	0.25
	Median	0.91	0.20	0.43	0.15	1.72
	Mean Rank	241.55	208.50	60.70	134.75	267.30
	Minimum	0.79	0.12	0.34	0.11	1.50
	Maximum	0.95	0.29	0.51	0.18	2.19
A9 (N=9)	Mean	0.83	0.20	0.44	0.14	1.77
	Std. Deviation	0.18	0.06	0.09	0.01	0.32
	Median	0.89	0.18	0.46	0.15	1.67
	Mean Rank	218.67	188.83	73.06	118.44	256.22
	Minimum	0.36	0.14	0.32	0.12	1.40
	Maximum	0.91	0.29	0.55	0.16	2.30
B1 (N=10)	Mean	0.81	0.10	0.71	0.15	1.21

	Std. Deviation	0.07	0.03	0.09	0.03	0.17
	Median	0.84	0.10	0.74	0.15	1.14
	Mean Rank	149.15	37.15	270.20	127.85	78.75
	Minimum	0.67	0.05	0.54	0.08	1.04
	Maximum	0.90	0.15	0.81	0.20	1.53
B2 (N=10)	Mean	0.85	0.18	0.56	0.17	1.42
	Std. Deviation	0.07	0.05	0.09	0.04	0.22
	Median	0.87	0.17	0.57	0.17	1.34
	Mean Rank	204.30	160.10	168.95	183.80	155.80
	Minimum	0.70	0.11	0.45	0.11	1.13
	Maximum	0.92	0.25	0.71	0.23	1.77
B3 (N=10)	Mean	0.83	0.17	0.58	0.16	1.38
	Std. Deviation	0.06	0.03	0.09	0.03	0.20
	Median	0.86	0.17	0.59	0.16	1.36
	Mean Rank	174.75	149.00	186.55	179.50	142.95
	Minimum	0.69	0.12	0.44	0.11	1.13
	Maximum	0.90	0.22	0.72	0.20	1.70
B4 (N=10)	Mean	0.84	0.18	0.53	0.16	1.49
	Std. Deviation	0.07	0.06	0.07	0.03	0.16
	Median	0.86	0.16	0.54	0.16	1.43
	Mean Rank	186.45	163.85	143.05	154.95	188.65
	Minimum	0.71	0.10	0.43	0.12	1.27
	Maximum	0.92	0.29	0.68	0.22	1.72
B5 (N=10)	Mean	0.86	0.16	0.56	0.15	1.42
	Std. Deviation	0.06	0.04	0.06	0.03	0.17
	Median	0.88	0.16	0.58	0.14	1.41
	Mean Rank	208.75	130.45	176.65	144.75	160.10
	Minimum	0.74	0.09	0.45	0.09	1.17
	Maximum	0.93	0.22	0.63	0.22	1.69
B6 (N=10)	Mean	0.85	0.20	0.51	0.16	1.51
	Std. Deviation	0.08	0.06	0.07	0.03	0.18
	Median	0.88	0.18	0.53	0.17	1.44
	Mean Rank	201.60	189.75	127.65	176.95	191.95
	Minimum	0.72	0.14	0.37	0.13	1.33
	Maximum	0.95	0.31	0.58	0.22	1.87
B7 (N=10)	Mean	0.85	0.20	0.52	0.16	1.50
	Std. Deviation	0.07	0.05	0.07	0.02	0.15
	Median	0.87	0.19	0.53	0.16	1.47
	Mean Rank	198.83	185.11	131.61	157.28	200.44
	Minimum	0.71	0.13	0.40	0.13	1.34
	Maximum	0.95	0.27	0.61	0.20	1.79
B8 (N=9)	Mean	0.88	0.17	0.55	0.15	1.44
	Std. Deviation	0.05	0.03	0.06	0.02	0.16
	Median	0.91	0.18	0.54	0.15	1.38
	Mean Rank	240.50	174.25	161.80	162.00	165.50
	Minimum	0.79	0.10	0.46	0.12	1.23
	Maximum	0.93	0.23	0.64	0.18	1.71
B9 (N=9)	Mean	0.83	0.18	0.54	0.16	1.45
	Std. Deviation	0.14	0.03	0.05	0.02	0.16
	Median	0.86	0.17	0.56	0.16	1.43
	Mean Rank	200.72	164.11	154.28	173.17	171.00
	Minimum	0.48	0.14	0.43	0.13	1.29
	Maximum	0.91	0.23	0.60	0.19	1.76
C1 (N=10)	Mean	0.84	0.07	0.85	0.14	1.06
	Std. Deviation	0.08	0.03	0.16	0.03	0.21
	Median	0.87	0.07	0.90	0.14	0.98
	Mean Rank	197.50	12.25	296.25	107.10	46.30
	Minimum	0.68	0.03	0.61	0.08	0.80
	Maximum	0.93	0.11	1.05	0.18	1.36
C2 (N=10)	Mean	0.86	0.15	0.67	0.17	1.23
	Std. Deviation	0.04	0.05	0.13	0.04	0.22
	Median	0.87	0.16	0.67	0.17	1.18
	Mean Rank	201.40	126.00	235.25	178.10	95.20
	Minimum	0.79	0.06	0.51	0.11	0.96
	Maximum	0.90	0.22	0.88	0.24	1.55
C3 (N=10)	Mean	0.85	0.14	0.69	0.16	1.22
	Std. Deviation	0.04	0.04	0.13	0.03	0.21

	Median	0.86	0.14	0.69	0.17	1.23
	Mean Rank	185.95	96.00	247.30	181.75	86.85
	Minimum	0.78	0.10	0.49	0.11	0.88
	Maximum	0.91	0.21	0.94	0.20	1.56
C4 (N=10)	Mean	0.83	0.16	0.63	0.16	1.29
	Std. Deviation	0.06	0.04	0.09	0.03	0.18
	Median	0.85	0.15	0.67	0.16	1.21
	Mean Rank	171.95	128.40	224.25	174.05	107.75
	Minimum	0.69	0.12	0.47	0.12	1.08
	Maximum	0.92	0.22	0.72	0.24	1.58
C5 (N=10)	Mean	0.86	0.15	0.66	0.16	1.26
	Std. Deviation	0.06	0.03	0.09	0.03	0.19
	Median	0.88	0.14	0.70	0.16	1.21
	Mean Rank	211.40	101.25	243.25	178.50	95.70
	Minimum	0.73	0.11	0.49	0.12	1.04
	Maximum	0.93	0.21	0.77	0.21	1.58
E1 (N=10)	Mean	0.71	0.19	0.68	0.19	1.16
	Std. Deviation	0.16	0.06	0.08	0.05	0.14
	Median	0.79	0.17	0.70	0.18	1.17
	Mean Rank	88.85	166.70	258.65	234.95	61.60
	Minimum	0.41	0.11	0.55	0.13	0.96
	Maximum	0.84	0.30	0.78	0.28	1.45
E2 (N=10)	Mean	0.56	0.20	0.62	0.18	1.28
	Std. Deviation	0.18	0.05	0.11	0.03	0.20
	Median	0.59	0.19	0.62	0.17	1.27
	Mean Rank	36.85	193.40	210.80	208.00	109.45
	Minimum	0.20	0.15	0.48	0.13	0.92
	Maximum	0.76	0.30	0.84	0.22	1.54
E3 (N=10)	Mean	0.62	0.23	0.58	0.19	1.35
	Std. Deviation	0.15	0.09	0.11	0.07	0.24
	Median	0.63	0.20	0.59	0.16	1.39
	Mean Rank	52.30	202.15	183.80	198.90	137.85
	Minimum	0.42	0.15	0.38	0.14	0.90
	Maximum	0.80	0.41	0.76	0.35	1.63
E4 (N=10)	Mean	0.71	0.14	0.68	0.15	1.23
	Std. Deviation	0.10	0.04	0.12	0.03	0.18
	Median	0.71	0.13	0.65	0.15	1.29
	Mean Rank	84.30	94.55	250.15	142.25	89.05
	Minimum	0.57	0.11	0.53	0.12	0.92
	Maximum	0.88	0.21	0.89	0.21	1.48
E5 (N=10)	Mean	0.71	0.23	0.57	0.19	1.34
	Std. Deviation	0.13	0.08	0.07	0.05	0.16
	Median	0.72	0.19	0.59	0.17	1.33
	Mean Rank	92.25	212.65	181.20	227.70	123.50
	Minimum	0.51	0.15	0.41	0.13	1.13
	Maximum	0.87	0.34	0.63	0.27	1.67
E6 (N=10)	Mean	0.70	0.18	0.58	0.17	1.27
	Std. Deviation	0.07	0.05	0.18	0.04	0.22
	Median	0.72	0.20	0.63	0.16	1.25
	Mean Rank	66.25	163.80	198.00	182.70	102.85
	Minimum	0.53	0.12	0.16	0.14	1.02
	Maximum	0.79	0.25	0.78	0.25	1.66
E7 (N=10)	Mean	0.77	0.18	0.65	0.18	1.26
	Std. Deviation	0.08	0.04	0.14	0.04	0.22
	Median	0.79	0.17	0.61	0.18	1.30
	Mean Rank	110.80	164.45	223.80	208.80	103.50
	Minimum	0.65	0.13	0.49	0.14	0.86
	Maximum	0.86	0.27	0.96	0.24	1.62
E8 (N=10)	Mean	0.68	0.24	0.53	0.18	1.45
	Std. Deviation	0.12	0.08	0.08	0.05	0.21
	Median	0.71	0.22	0.52	0.16	1.51
	Mean Rank	69.00	224.05	137.90	191.15	175.25
	Minimum	0.46	0.14	0.43	0.13	1.07
	Maximum	0.82	0.36	0.66	0.28	1.70
E9 (N=10)	Mean	0.72	0.21	0.55	0.18	1.39
	Std. Deviation	0.16	0.05	0.09	0.03	0.18
	Median	0.78	0.21	0.56	0.16	1.38

	Mean Rank	104.61	211.06	150.17	201.39	167.17
	Minimum	0.35	0.14	0.45	0.13	1.07
	Maximum	0.86	0.34	0.72	0.23	1.60
E10 (N=9)	Mean	0.64	0.20	0.48	0.16	1.46
	Std. Deviation	0.06	0.05	0.14	0.03	0.12
	Median	0.62	0.19	0.52	0.15	1.49
	Mean Rank	43.80	192.60	134.25	162.50	168.05
	Minimum	0.57	0.13	0.13	0.12	1.25
	Maximum	0.77	0.29	0.61	0.21	1.64
Total	Mean	0.80	0.19	0.56	0.16	1.45
	Std. Deviation	0.13	0.07	0.14	0.04	0.30
	Median	0.84	0.17	0.55	0.15	1.42
	Minimum	0.20	0.03	0.13	0.08	0.80
	Maximum	0.95	0.51	1.05	0.35	2.47
