# Supplementary figures and images for: Trabecular Bone Ontogeny of the Human Distal Tibia
Source: Am J Biol Anthropol. 2024 Dec 8;186(1):e25043. doi: 10.1002/ajpa.25043 (PMC11775436; doi:10.1002/ajpa.25043)

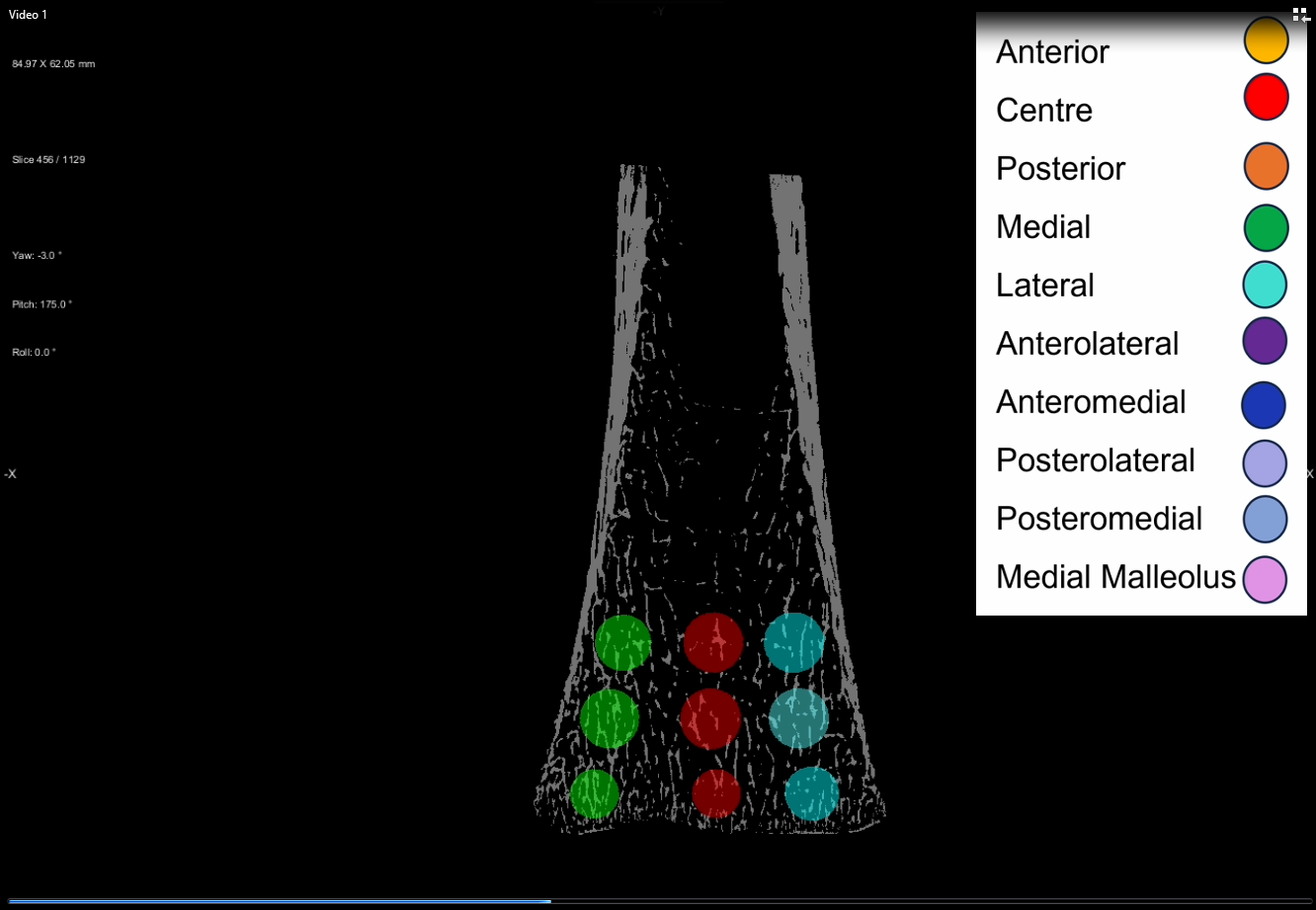

Supplement: Supplementary file 3 — Video S2 Image. [file AJPA-186-e25043-s001.tif]

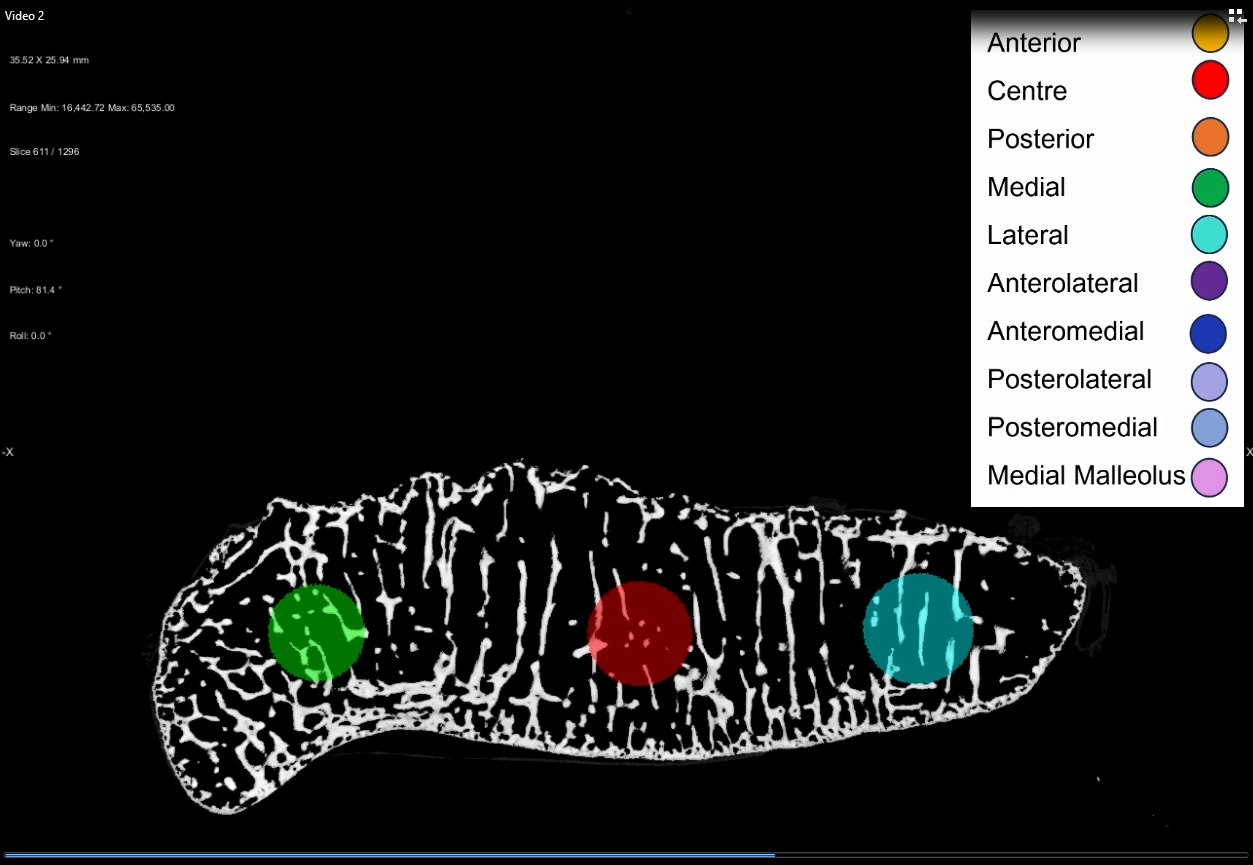

Supplement: Supplementary file 5 — Video S4. Image. [file AJPA-186-e25043-s005.tif]
